# Paclitaxel/Luteolin Coloaded Dual‐Functional Liposomes for Esophageal Cancer Therapy

**DOI:** 10.1002/advs.202411930

**Published:** 2025-04-23

**Authors:** Congyong Sun, Fei Xie, Huiyun Zhang, Lulu Feng, Yuting Wang, Chaofan Huang, Zhizhen Cui, Chao Luo, Li Zhang, Qilong Wang

**Affiliations:** ^1^ The Comprehensive Cancer Center Department of Central Laboratory The Affiliated Huai'an No.1 People's Hospital Nanjing Medical University Huai'an Jiangsu 223300 China; ^2^ Department of Pharmaceutical Engineering School of Chemistry and Chemical Engineering Yancheng Institute of Technology Yancheng Jiangsu 224003 China; ^3^ Department of Acute Infectious Disease Control and Prevention Huai'an Center for Disease Control and Prevention Huai'an Jiangsu 223003 China

**Keywords:** combinational chemotherapy, DNA aptamer, esophageal squamous cell carcinoma, pH‐sensitive liposome, targeted delivery, tumor microenvironment (TME)

## Abstract

Combination therapy integrating chemotherapeutic agents with natural bioactive ingredients represents an attractive strategy for esophageal squamous cell carcinoma (ESCC) treatment, yet achieving tumor‐specific co‐delivery remains a critical challenge. Herein, we report that the combination of luteolin (LUT) and paclitaxel (PTX) exerts a remarkable synergy in ESCC treatment, while concurrently alleviating PTX‐induced hepatotoxicity; EA2 aptamer has been identified for its exceptional specificity and strong affinity toward Catenin Alpha 1 protein (CTNNA1) in ESCC cells. Leveraging this specificity, nanosized EA2‐modified pH‐sensitive liposomes (EA2‐PSL‐PTX/LUT) are successfully developed with effective co‐loading, controlled release, and good biostability. EA2‐PSL‐PTX/LUT exhibits stimuli‐triggered release in the acidic tumor microenvironment and facilitates specific cellular uptake and endosomal escape in ESCC cells. In vivo imaging confirms precise tumor localization, deep tumor penetration, and prolonged retention of the nanocarrier. In vitro and in vivo findings validate that the nanocarrier potentiates synergistic inhibitions of PTX and LUT. Notably, EA2‐PSL‐PTX/LUT significantly activates the tumor microenvironment by promoting dendritic cell maturation and T cell infiltration. And the immunosuppressive microenvironment has been remodeled by decreasing myeloid‐derived suppressor cells and regulatory T cell accumulation. This study provides a strategy for precise delivery of combinational chemotherapeutic drugs for ESCC targeted therapy.

## Graphical abstract

The authors develop a pH‐sensitive aptamer‐modified liposome (EA2‐PSL‐PTX/LUT) to illustrate a strategy for the precise delivery of combinational chemotherapeutic drugs for esophageal squamous cell carcinoma (ESCC) targeted therapy. EA2‐PSL‐PTX/LUT provides efficient co‐loading, tumor targeting, and controlled release of luteolin and paclitaxel, exhibits excellent ESCC cancer suppression efficiency with alleviated hepatotoxicity, and reeducates the immunosuppressive microenvironment into an immune‐activated state.

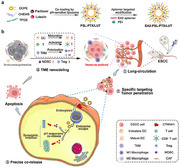

## 1 Introduction

Esophageal cancer is a major gastrointestinal malignancy, ranking 11th in global incidence and 7th in mortality among malignant tumors.^[^ ^1^ ^]^ Notably, China has the highest incidence rate, contributing to 9.9% of cancer‐related fatalities.^[^ ^2^ ^]^ More than 90% of esophageal cancer cases in China are esophageal squamous cell carcinoma (ESCC), which is characterized by insidious progression, high malignancy, strong invasiveness, and poor prognosis. While early diagnosis and treatment^[^ ^3^ ^]^ have led to a decline in ESCC incidence, the overall five‐year survival rate remains low at 20.9–30.3%.^[^ ^4^ ^]^ Chemotherapy is essential for treating advanced and metastatic ESCC,^[^ ^5^ ^]^ and paclitaxel (PTX; Figure S1a, Supporting Information) is the key therapeutic agent. PTX can bind to β‐tubulin, inhibit microtubule depolymerization during cell division and mitosis, and thereby inducing cell cycle arrest and apoptosis.^[^ ^6^ ^]^ However, effective clinical practice remains hindered. The low solubility of PTX leads to inadequate membrane transport, rapid blood clearance, and low bioavailability.^[^ ^7^ ^]^ Additionally, owing to its limited targeting to tumors, higher doses are required, resulting in nonspecific binding and intolerable cytotoxicity. Repeated chemotherapy can also provoke host responses that create a tumor‐friendly microenvironment, promoting tumor metastasis and multidrug resistance.^[^ ^8^ ^]^ Furthermore, PTX and its metabolites can also cause hepatotoxicity.^[^ ^9^ ^]^ These challenges reduce the overall efficacy and contribute to ESCC treatment failure. Therefore, it is critical to address the limitations of single‐agent chemotherapy.

Combination therapy has emerged as an attractive approach.^[^ ^10^ ^]^ Notably, combination chemotherapy that integrates chemotherapeutic drugs with bioactive ingredients shows great potential for treating cancer.^[^ ^11^ ^]^ Advanced high‐throughput screening technologies have revealed several bioactive compounds from traditional Chinese medicine, such as jesridonin,^[^ ^12^ ^]^ cinobufagin,^[^ ^13^ ^]^ and schizandrin A,^[^ ^14^ ^]^ that synergistically inhibit ESCC along with PTX, offering promising prospects for clinical application. These compounds can reduce dosage and toxicity while improving efficacy by modulating the tumor microenvironment, inhibiting angiogenesis, mitigating multidrug resistance, inducing apoptosis, and activating antitumor immune responses.^[^ ^15^ ^]^

Luteolin (LUT; Figure S1b, Supporting Information), a natural flavonoid possesses antioxidant properties,^[^ ^16^ ^]^ affects the polarization function of macrophages in the tumor microenvironment (TME),^[^ ^17^ ^]^ and exhibits antiproliferative effects against various cancer cells.^[^ ^18^, ^19^ ^]^ Recent studies have demonstrated that combining LUT with PTX synergistically inhibits ESCC by suppressing epithelial–mesenchymal transition, which reduces cell migration and induces apoptosis.^[^ ^20^ ^]^ More importantly, LUT exhibits potent hepatoprotective effects against liver injury in mice.^[^ ^21^ ^]^ Given the hepatotoxicity associated with PTX chemotherapy, the utilization of hepatoprotective agents, such as LUT, is particularly appealing. In our preliminary studies, LUT counteracted PTX‐induced hepatocyte damage and proliferation inhibition and improved liver function parameters in AML12 cells in vitro. As such, the combination of PTX and LUT presents a promising strategy for enhancing the anti‐ESCC efficacy and remodeling the tumor microenvironment while mitigating hepatotoxicity. However, there is an urgent need for therapeutics to codeliver PTX and LUT at specific ratios to target cancer cells.

Various nanotechnology‐based drug delivery systems (NDDS), such as nanoparticles,^[^ ^22^ ^]^ micelles,^[^ ^23^ ^]^ nanoemulsions,^[^ ^24^ ^]^ and liposomes,^[^ ^25^ ^]^ have significantly improved the efficiency of combination chemotherapy. These nanocarriers enable effective co‐loading of chemotherapeutic agents, enhancing their synergistic effects by synchronizing exposure in tumor cells. They also offer several advantages, such as good biocompatibility, enhanced drug solubility, prolonged in vivo circulation time, increased bioavailability via the enhanced permeability and retention (EPR) mechanism, and promote accumulation in tumors.^[^ ^26^, ^27^ ^]^ Substantial progress has been made in the design of stimulus‐responsive NDDS based on TME (e.g., pH, redox state, and enzymes) to achieve controlled and sustained drug release in tumor tissues and cells. pH‐sensitive liposomes (PSL), a specific types of liposomal NDDS predominantly incorporating 1,2‐dioleoyl‐sn‐glycero‐3‐phosphoethanolamine (DOPE) and the weakly amphiphilic acid cholesterol hemisuccinate (CHEMS),^[^ ^28^ ^]^ are designed to respond to acidic environments and release encapsulated drugs. By leveraging this unique feature, PSL can achieve precise, targeted, and controlled delivery of anticancer agents directly to tumor sites, maximizing treatment efficacy while minimizing systemic toxicity. However, despite the exceptional performance of PSL, only 0.7% of intravenously administered NDDS successfully reaches the tumor site in clinical practice.^[^ ^29^ ^]^ This limited targeting is primarily due to passive targeting efficiency, which can result in suboptimal tumor accumulation, limited intratumoral passive diffusion, and insufficient tissue selectivity at the tumor sites.^[^ ^30^ ^]^ Additionally, the dense nature of tumor tissue also poses challenges for PSL penetration, necessitating overcoming barriers, such as the vascular endothelium^[^ ^31^ ^]^ and extracellular matrix,^[^ ^32^ ^]^ to enter the tumor interstitium.

Actively targeted modified liposomes can overcome the limitations of nonspecific targeting. Various ligands based on the overexpression of cancer cell surface molecules can be incorporated into liposomes, thereby facilitating targeted cellular uptake. One innovative approach involves the use of the cell‐systematic evolution of ligands by exponential enrichment (Cell‐SELEX) technology,^[^ ^33^ ^]^ which allows for the identification of single‐stranded RNA or DNA (aptamers) with high affinity for tumor cells. Aptamers offer several advantages, including low molecular weight, minimal immunogenicity, ease of synthesis and modification, high specificity, remarkable affinity, and efficient tumor penetration.^[^ ^34^ ^]^ Hence, they are increasingly being acknowledged as a premier strategy for targeted nanoparticle drug delivery. We have previously reported that an engineered EA1 aptamer‐modified nano‐system significantly enhanced drug and gene delivery to tumors, exhibiting excellent ESCC suppression efficiency.^[^ ^35^ ^]^

**Scheme 1:**
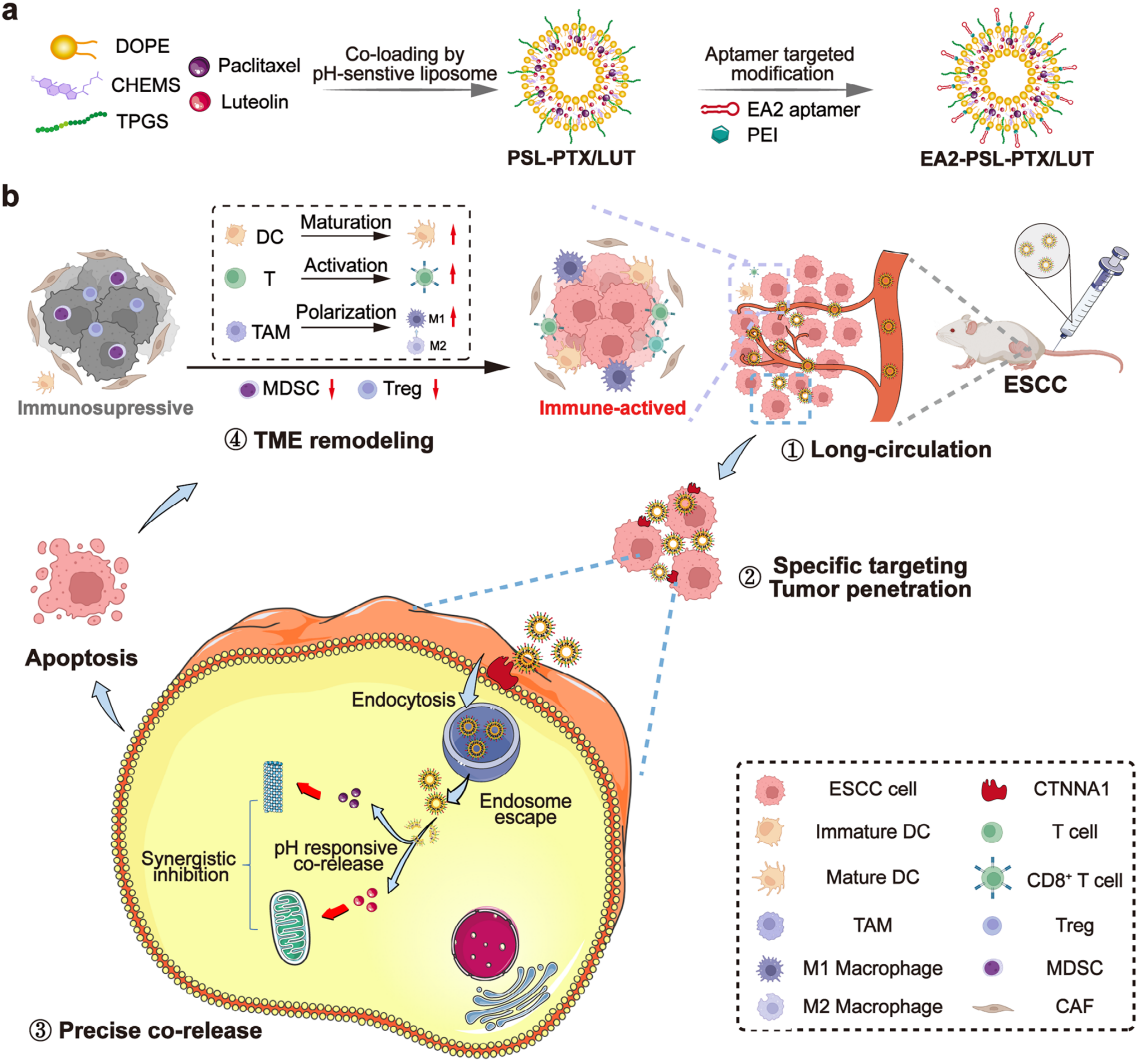
a) Schematic of a targeted co‐delivery system for PTX and LUT via EA2 aptamer‐modified PSL. b) Our hypothesis posits that precise co‐delivery of PTX and LUT via nano‐sized, PSL, functionalized with EA2 aptamers, could facilitate efficient co‐loading, targeted delivery, and controllable co‐release of the PTX/LUT within ESCC tumor/cells, achieving reduced hepatotoxicity, enhanced synergistic chemotherapy, and remodeled the immunosuppressive microenvironment for ESCC treatment.

In this study, we aimed to develop a nanosized liposomal codelivery system (EA2‐PSL‐PTX/LUT) with a core–shell structure (**Scheme** **1**) to realize efficient coloading, active targeting, and controllable corelease of PTX and LUT in ESCC tumors/cells, achieve reduced toxicity and enhanced efficacy, and remodel the immunosuppressive microenvironment in treating ESCC. This system was constructed using a lipid material comprising DOPE, CHEMS, and D‐alpha‐tocopheryl polyethylene glycol succinate (TPGS) for efficient coloading of PTX and LUT within the PSL core, while the shell was modified with the EA2 aptamer to specifically target ESCC cells. EA2‐PSL‐PTX/LUT achieved high coloading efficiency, responsive controlled release, and good biostability. Furthermore, enhanced intracellular uptake and endosomal escape ability were validated in ESCC cells. Specific tumor targeting, prolonged retention, and deep tumor penetration of EA2‐PSL‐PTX/LUT were confirmed in tumor‐bearing mice. Both in vivo and in vitro experiments demonstrated the synergistic efficacy and safety of EA2‐PSL‐PTX/LUT for the treatment of ESCC. Additionally, based on the human peripheral blood mononuclear cell (PBMC)‐engrafted tumor‐bearing mice model, EA2‐PSL‐PTX/LUT was confirmed to remarkably rebuild the immunosuppressive microenvironment of ESCC tumors by reeducating the TME into an immune‐activated state, decreasing immunosuppressive‐related cell infiltration, and softening the stromal barrier. Overall, this study presents an innovative strategy for precise codelivery of chemotherapeutic agents to improve targeted therapy outcomes for ESCC.

## 2 Results and Discussion

### 2.1 LUT and PTX Exert a Remarkable Synergistic Inhibition on ESCC with Alleviated Hepatotoxicity

**Figure 1:**
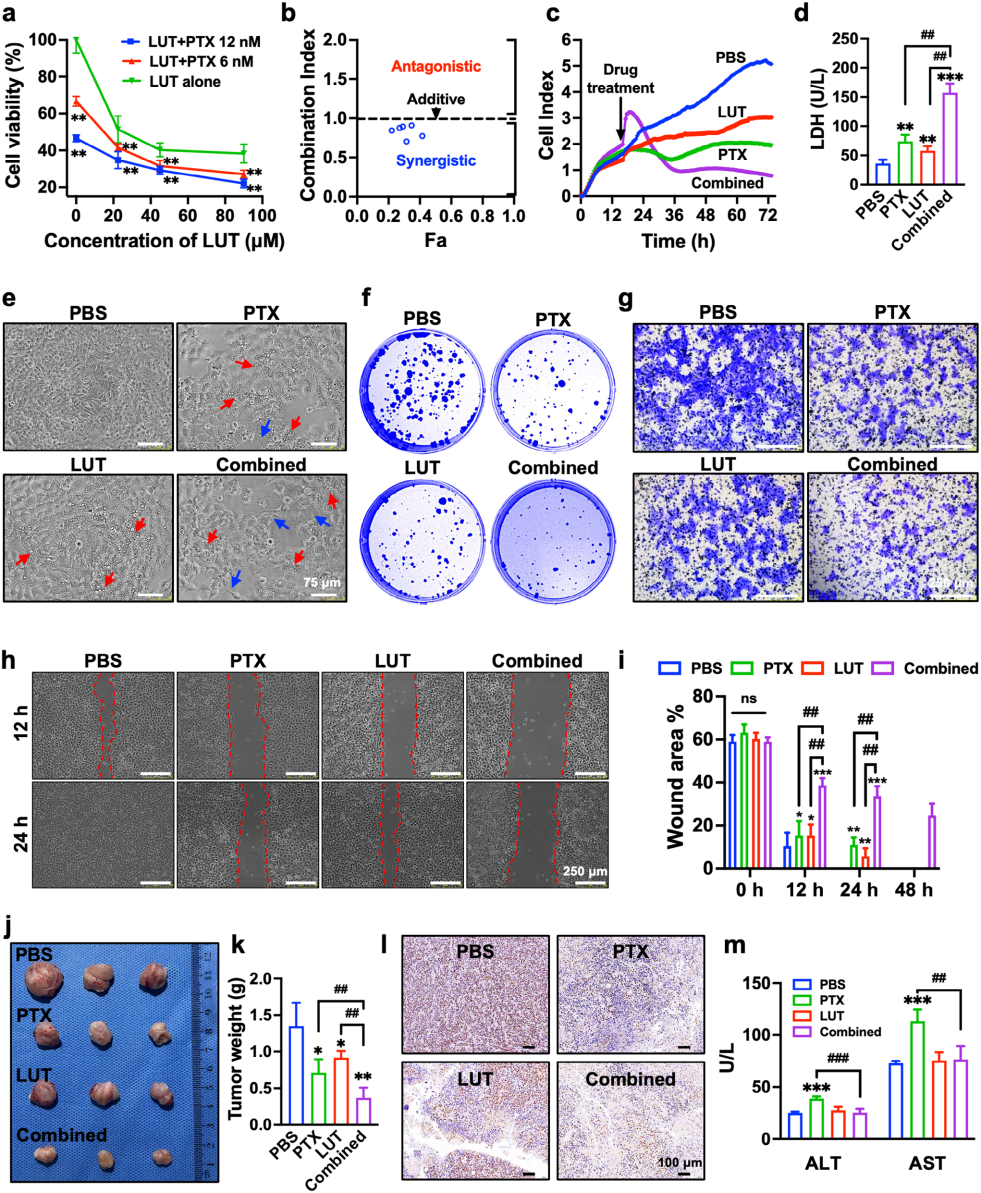
LUT and PTX exerts a remarkable synergistic inhibition on ESCC with alleviated hepatotoxicity in vitro and in vivo. a) Cell viabilities of KYSE‐150 cells treated with PTX (6 and 12 nM) or in combination with LUT (22.5, 45, and 90 µM) for 48 h using CCK‐8 assay. b) CI values of PTX combined with LUT using the CompuSyn software. c) Effects of combined PTX and LUT on proliferative ability of KYSE‐150 cells evaluated by RTCA assay, treatments include PBS, PTX (12 nM), LUT (22.5 µM), and Combined (PTX, 12 nM and LUT, 22.5 µM). d) LDH leakage of KYSE‐150 cells after the treatment of combined PTX and LUT for 24 h. e) Representative morphological changes of KYSE‐150 cells after the treatments for 24 h. Red arrows: vacuolation degeneration, blue arrows: pseudopod. Scale bars, 75 µm. f) Effects of combined PTX and LUT on colony formation ability of KYSE‐150 cells. g) Effects of combined PTX and LUT on cell invasion of KYSE‐150 cells using Transwell assays. Scale bars, 250 µm. h‐i) Migration abilities of treated KYSE‐150 cells by wound‐healing assay. Scale bars, 250 µm. j‐k) Tumor photographs and tumor weights of isolated tumor tissues after various treatments, including PBS, PTX (5 mg kg^−1^), LUT (50 mg kg^−1^), and Combined (PTX, 5 mg kg^−1^ and LUT, 50 mg kg^−1^). l) Representative micrographs of Ki67 staining of tumor tissues. Scale bars, 100 µm. m) Serum hepatic function indicators (ALT and AST) after different treatments. For in vitro assay, all data expressed as mean ± SD (*n* = 5); for in vivo anti‐tumor study, all data expressed as mean ± SD (*n* = 3). Statistical significance between different groups was obtained by one‐way ANOVA using the Tukey's post‐test (a, d, i, k, m). ^*^ *p* < 0.05, ^**^ *p* < 0.01, ^***^ *p* < 0.001, significant as compared to the PBS group. ^##^ *p* < 0.01, ^###^ *p* < 0.001, significant as compared to PTX or LUT group.

In preliminary experiments, we used the CCK‐8 assay to assess the effects of PTX and LUT on the proliferation of KYSE‐150 cells. The drugs exhibited a time‐ and dose‐dependent inhibition of cell proliferation. The IC_50_ values for PTX and LUT at 48 h were 12.82 nM and 44.25 µM (Figure S1c‐d, Supporting Information), respectively. A combinatorial drug experiment was designed using the CompuSyn software to evaluate the synergistic effects of PTX and LUT at dosages ranging from 1/2 IC_50_ to 2 IC_50_. Individual or combined administration of PTX and LUT resulted in substantial inhibition of cell growth (**Figure** **1a**), with the PTX+LUT combination group demonstrating a more pronounced inhibitory effect than the individual drug groups. Subsequently, the CompuSyn software was used to calculate the combination index (CI) for LUT and PTX. The CI curve (Fa‐CI plot) is illustrated in Figure 1b. All CI values were below 1, indicating robust synergistic cytotoxicity in KYSE‐150 cells. With a minimum CI of 0.704, the actual inhibition rate reached 68.52% at a concentration of 12 nM PTX and 22.5 µM LUT, which were selected for subsequent experiments.

Next, the cytotoxic effects of PTX and/or LUT were evaluated using real‐time cell analysis (RTCA), lactate dehydrogenase (LDH) leakage, cell morphology, and colony formation assays. The cell index values obtained from the RTCA were monitored for 72 h (Figure 1c). Compared to treatment with PTX or LUT alone, the combined treatment resulted in a marked decrease in the cell index. LDH is a crucial enzyme in intracellular oxidative phosphorylation, and the death or apoptosis of cancer cells can lead to elevated LDH release. We observed a significant elevation in LDH levels (Figure 1d) following the combination treatment. Additionally, KYSE‐150 cells exhibited a polygonal shape with well‐defined cell borders and a homogeneous distribution (Figure 1e; Figure S1e, Supporting Information). Conversely, after treatment with PTX and/or LUT for 48 h, striking alterations in cell morphology were observed. The cells exhibited a substantial decrease in number and appeared shrunken and rounded, with enlarged cytoplasmic vacuoles and extended pseudopods. They displayed a more disorganized arrangement, which was particularly pronounced in the combined treatment group. Consistent with the RTCA findings, colonies subjected to the combination treatment displayed reduced size and fewer numbers than those treated with either drug individually (Figure 1f; Figure S1f, Supporting Information), and the colony formation rate was markedly diminished following the combination treatment (Figure S1g, Supporting Information). Furthermore, Transwell and wound healing assays were employed to assess the synergistic effects on cell migration and invasion. Compared to treatment with either drug alone, the combined treatment notably augmented the inhibition of cell migration, as evidenced by the reduced number of invaded cells (Figure 1g; Figure S1h, Supporting Information) and larger scratched wound area (Figure 1h‐i; Figure S1i, Supporting Information).

The cytotoxic effects of PTX and LUT, administered alone or in combination, were further assessed in nude mice bearing KYSE‐150 tumors. The body weight of mice in each group gradually increased throughout the treatment period (Figure S2a, Supporting Information), with no notable differences observed. Notably, both PTX and LUT showed significant antitumor effects. Moreover, compared with the PBS control group or the groups treated with PTX or LUT alone, the cotreatment group displayed more pronounced inhibition of cell growth, as evidenced by the tumor growth profile (Figure S2b, Supporting Information), tumor size (Figure 1j), and tumor weight (Figure 1k). Hematoxylin and eosin (HE) staining, terminal deoxynucleotidyl transferase dUTP nick‐end labeling (TUNEL) assay, and Ki67 staining revealed that the combination treatment markedly augmented apoptosis and suppressed the proliferation rates. Specifically, HE staining (Figure S2c, Supporting Information) revealed a dense cluster of tumor cell nuclei with a relatively uniform, spherical morphology in the PBS‐treated group. Conversely, treatment with PTX or LUT alone induced morphological alterations, such as condensed nuclei, reduced cytoplasmic and nuclear staining, cytoplasmic vacuolization, and cell shrinkage, particularly in the cotreatment group. Compared to the PTX or LUT alone groups, the cotreatment group exhibited a decrease in the brown‐stained area in the Ki67 assay (Figure 1l), whereas in the TUNEL assay (Figure S2d, Supporting Information), an increase in the red fluorescence signal was observed. Those findings indicated that the cotreatment synergistically promoted apoptosis and inhibited cell proliferation in vivo. Moreover, hepatic function indexes (alanine aminotransferase (ALT), aspartate aminotransferase (AST), and LDH), as depicted in Figure 1m and Figure S2e, Supporting Information) exhibited marked elevations in the PTX‐treated group but were mitigated in the cotreatment group. These observations highlight the hepatoprotective properties of LUT against PTX‐induced hepatotoxicity.

Subsequently, AML12 cells were exposed to varying concentrations of PTX. The IC_50_ value was determined to be 150 nM, which was used for PTX‐induced hepatic damage (Figure S3a, Supporting Information). Compared with the control group, the PTX treatment group exhibited pronounced cytotoxicity, as evidenced by decreased cell viability and increased LDH leakage. Conversely, pretreatment with different concentrations of LUT for 12 h led to a dose‐dependent increase in cell viability (Figure S3b, Supporting Information) and a distinct reduction in LDH leakage (Figure S3c, Supporting Information), indicating a significant protective effect of LUT against PTX‐induced hepatocytotoxicity. Additionally, LUT significantly attenuated the elevation of ALT, AST, alkaline phosphatase (AKP), and malondialdehyde (MDA) levels induced by PTX, while increasing superoxide dismutase (SOD) levels (Figure S3d‐h, Supporting Information). These results highlighted the efficacy of LUT in improving liver function indicators in AML12 cells, mitigating oxidative stress, and reducing lipid peroxidation. Cell morphology assessment (Figure S3i, Supporting Information) revealed a decrease in cell number and cell shrinkage caused by PTX. However, LUT treatment significantly mitigated these changes in cell morphology. The 5‐ethynyl‐2′‐deoxyuridine (EdU) assay (Figure S3j‐k, Supporting Information) further confirmed that LUT counteracted the PTX‐induced reduction in cell proliferation. We further conducted Western blotting analysis of key proteins involved in the Nrf2/ARE antioxidant pathway in PTX‐treated AML12 cells. Results demonstrated that LUT pretreatment significantly upregulates Nrf2, HO‐1, and NQO1 expression while reducing Keap1 activation (Figure S3l‐m, Supporting Information). Our findings suggest that LUT has chemo‐preventive potential against PTX‐induced hepatotoxicity plausibly through the attenuation of oxidative stress.

Consistent with the in vitro results, PTX‐induced liver injury was also observed in mice (Figure S4a, Supporting Information). Notably, LUT treatment substantially ameliorated the loss of liver function in a dose‐dependent manner, as evidenced by the reduced serum ALT and AST levels (Figure S4b‐c, Supporting Information), decreased MDA levels (Figure S4d, Supporting Information), and elevated hepatic SOD levels (Figure S4e, Supporting Information). These results underscore the ability of LUT to enhance the antioxidant capacity of the liver tissue and suppress lipid peroxidation. The hepatic coefficient (Figure S4f, Supporting Information) further indicated that PTX induced hepatomegaly in mice, which was alleviated by LUT. Consistent with these biochemical findings, HE staining (Figure S4g, Supporting Information) revealed that PTX triggered significant inflammation, loss of cell boundaries, cytoplasmic vacuolization, and necrosis in the liver, whereas LUT ameliorated these pathological changes. Collectively, the in vitro and in vivo data clearly demonstrate that LUT and PTX exert a remarkable synergistic inhibition on ESCC with alleviated hepatotoxicity.

### 2.2 Aptamer EA2 Exhibits High Binding Affinity and Specificity for CTNNA1 in ESCC Cells

**Figure 2:**
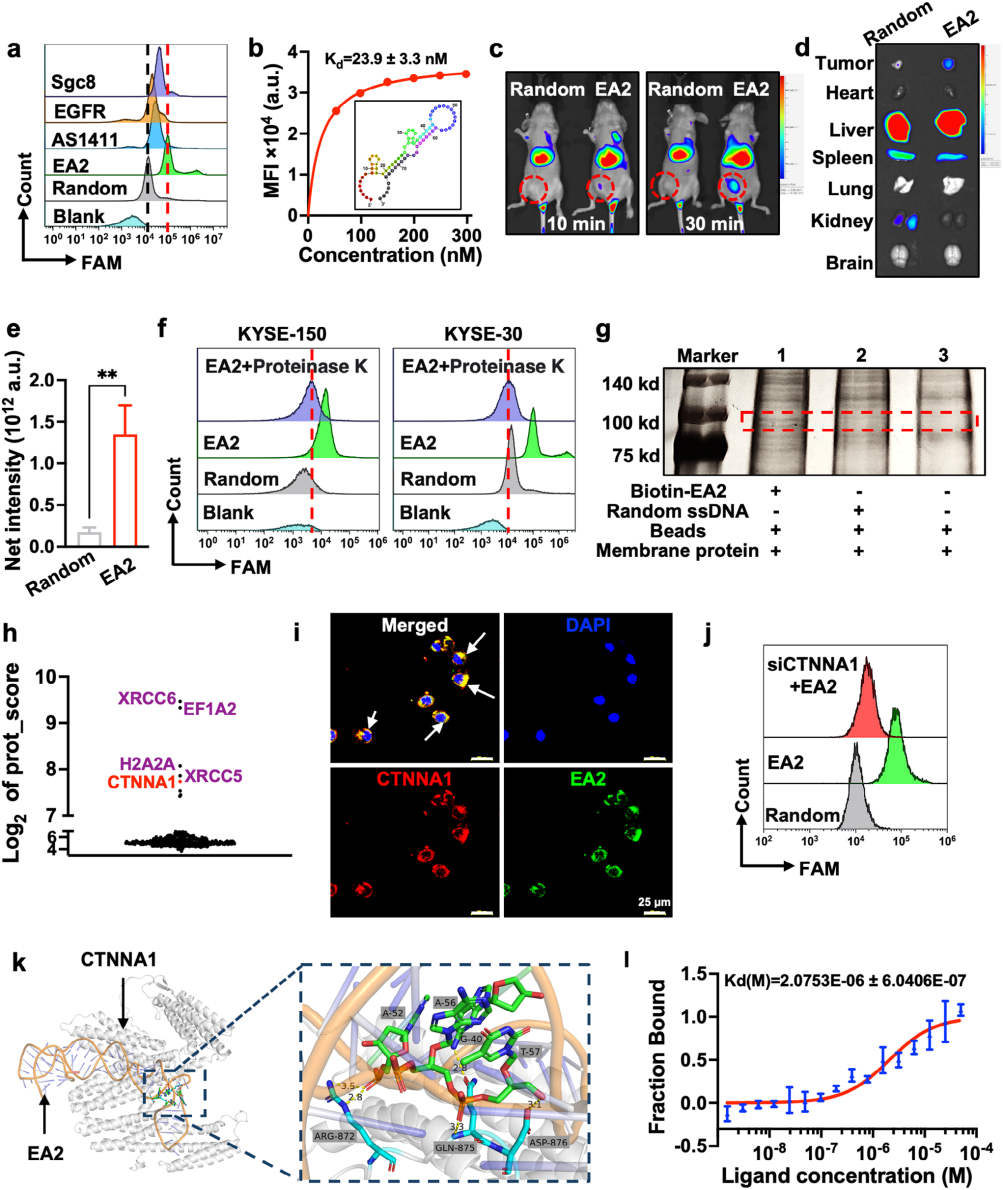
Specificity, binding target, and in vivo targeting characterization of the ESCC‐specific aptamer EA2. a) Comparison of the binding specificity of FAM‐labeled aptamer EA2, AS411, EGFR, and Sgc8 to target KYSE‐150 cells. b) Determination of the dissociation constant (*K*_d_) of EA2 to KYSE‐150 cells; predicted secondary structure of EA2 by mFold was embedded. c‐d) In vivo targeting specificity and biodistribution of Cy5‐labeled random ssDNA (5 nmol) and EA2 (5 nmol) in ESCC subcutaneous tumor xenograft mice model. e) Fluorescence intensity in tumors. f) Determination of the target type using proteinase K treatments. g) Differential band analysis by SDS‐PAGE after ssDNA pull‐down assays. KYSE‐150 cell membrane proteins were incubated with biotin‐EA2‐labeled beads (lane 1), random ssDNA‐labeled beads (lane 2), and beads only (lane 3). SDS‐PAGE was used to analyze the captured proteins, and the gel was silver‐stained. The red dashed box indicates the specific band. h) CTNNA1 was identified as the binding target of EA2 by differential analysis of protein abundance and scores. i) Confocal imaging and colocalization analysis of FAM‐labeled EA2 and CTNNA1 antibody in KYSE‐150 cells. Blue: cell nuclei; green: EA2; red: CTNNA1 protein in cell membrane. White arrows: colocalization, Scale bars, 20 µm. j) Flow cytometry assays of the binding of FAM‐labeled EA2 to KYSE‐150 cells treated with the siCTNNA1. k) Computational binding model, key interactions, and an enlarged image of the binding interfaces between EA2 and CTNNA1 by molecular docking using the HDOCK software. The key residues (amino acids) interacting with EA2 were depicted in cyan sticks, while the interacting base sites of EA2 were highlighted in green. l) Kinetic constant (*K_d_*) analyses of CTNNA1 interacting with EA2 using microscale thermophoresis assay. All data expressed as mean ± SD (*n* = 3), statistical significance between different groups was obtained by an unpaired two‐tailed Student's t test (**e**). ^**^ *p* < 0.01, significant as compared to the random ssDNA group.

As depicted in Figure S5a (Supporting Information), our previous study^[^ ^35^ ^]^ enabled the enrichment of aptamers, leading to the discovery of an EA2 aptamer that binds strongly to esophageal cancer cells. The significant and specific binding of EA2 to KYSE‐150 cells was confirmed using confocal imaging (Figure S5b, Supporting Information). EA2 exhibited an excellent binding ability to target cells at 37 °C, indicating its potential for in vivo applications. Further assessment of the binding selectivity of EA2 via flow cytometry (Figure S5c‐d, Supporting Information) demonstrated its excellent affinity for various human ESCC cell lines (KYSE‐150, KYSE‐30, KYSE‐410, and Eca9706) and low affinity for other cancer cell lines (MCF‐7, AGS, Nalm‐6, SW620, HeLa, A431, A549, U251, and HepG2), indicating that EA2 exhibits high binding affinity and specificity for ESCC cells. In contrast to other ESCC‐specific aptamers documented,^[^ ^36^, ^37^, ^38^ ^]^ such as Sgc8, AS411, and EGFR (Table S1, Supporting Information), EA2 exhibited significantly higher specificity (**Figure** **2a**). Moreover, the dissociation constant (Figure 2b) was calculated to be within the nanomolar range (23.9 ± 3.3 nM), suggesting the high binding affinity of EA2 for KYSE‐150 cells. The predicted secondary structure of EA2 is shown in Figure 2b. We further validated the in vivo targeting of EA2 using a subcutaneous xenograft mice model. Compared with random ssDNA, significantly higher fluorescence signals were observed in the tumor and liver regions at 10 and 30 min after EA2 administration (Figure 2c). The considerably increased fluorescence intensity (Figure 2d‐e) further demonstrated that EA2 could target ESCC tumors and undergo liver metabolism. Then, clinical tumor tissues were used to confirm the specific binding of EA2 to ESCC cells. Prominent pink fluorescence signals were detected in the ESCC tissue (Figure S5e, Supporting Information), with no specific binding observed in other tumor types (breast, cervical, colorectal, gastric, liver, and ovarian cancers). These findings indicate that EA2 could serve as a specific recognition probe for clinical diagnosis and targeted therapy of ESCC.

To determine the target type of EA2, KYSE‐150 and KYSE‐30 cells were treated with proteinase K for the degradation of surface proteins. Compared with that in untreated cells, fluorescence signals notably decreased following proteinase K pretreatment (Figure 2f), indicating that the targets of EA2 were likely membrane proteins. To identify the target, biotinylated EA2 was synthesized for ssDNA pull‐down experiments. Cell membrane proteins were incubated separately with biotinylated EA2 and controls, and the binding complex was purified using streptavidin‐modified magnetic beads and analyzed using SDS‐PAGE. A discernible protein band at ≈100 kDa was observed in the EA2 lane but not in the bead or random ssDNA lanes (Figure 2g; Figure S6a, Supporting Information), indicating the specific binding of EA2. Specific protein bands were further purified and analyzed via mass spectrometry (MS) using the Proteome Discovery 1.3. A list of protein candidates was obtained (Figure 2h; Figure S6b, Supporting Information), with CTNNA1 scoring the highest and having the most matched peptides known to be highly expressed in the cell membrane of ESCC cells.^[^ ^39^ ^]^ To verify the MS results, the expression of CTNNA1 in ESCC cell lines was assessed using Western blotting. As illustrated in Figure S6c (Supporting Information), KYSE‐150, KYSE‐30, KYSE‐410, and Eca9706 cells exhibited markedly elevated levels of CTNNA1 compared with that in HEEC. Additionally, confocal imaging (Figure 2i) revealed colocalization of EA2 and CTNNA1. Subsequent knockdown experiments using specific siRNAs (Table S2, Supporting Information) targeting CTNNA1 in KYSE‐150 cells revealed a notable reduction in CTNNA1 expression (Figure S6d, Supporting Information). Then, flow cytometry analysis of KYSE‐150 cells treated with siCTNNA1 (Figure 2j) showed decreased binding, confirming the essential role of CTNNA1 in the interaction between EA2 and KYSE‐150 cells. These results confirm CTNNA1 as the molecular target of EA2.

To further investigate the binding sites between CTNNA1 and EA2, 3D structures of CTNNA1 and EA2 (Figure S6e‐f, Supporting Information) were constructed, followed by molecular docking to identify the optimal model through molecular minimization. The cluster conformation of the complex structure (Figure S6g, Supporting Information) revealed that EA2 binds to the extracellular domain of CTNNA1. A magnified view (Figure 2k) further elucidates the key interactions between the amino acids of CTNNA1 and the base sites of EA2. Following structural preparation and model optimization, the interactions between CTNNA1 and EA2 were assessed and the amino acid residues involved in these interactions were documented (Table S3, Supporting Information). In terms of interaction frequencies, the interaction between CTNNA1 and EA2 was predominantly mediated by van der Waals forces. The CTNNA1 amino acids, such as GLN875, ASP876, ARG872, and ASP561, were predicted to be pivotal for binding to key residues. Subsequently, the binding affinity between CTNNA1 and EA2 was validated using microscale thermophoresis (MST). The changes in the fluorescence intensity over time in the MST experiments are shown in Figure S6h (Supporting Information), with a high signal‐to‐noise ratio (12.26), confirming the high quality of the data. The strength of the molecular interactions between CTNNA1 and EA2 was monitored by plotting the normalized fluorescence intensity against the EA2 concentration (Figure S6i, Supporting Information). The electrophoresis rates in the capillaries significantly increased with the increase in EA2 concentration, resulting in an “S”‐shaped fitting curve that indicated a pronounced and strong concentration dependency on the upper and lower plateaus. The *K*_d_ values (2.07 ± 0.60 µM) obtained from MST assay (Figure 2l) further corroborated the specific binding between CTNNA1 and EA2. Additionally, to investigate the competitive binding mechanism between EA2 and CTNNA1, we adopted a multi‐pronged experimental approach, including flow cytometry, immunofluorescence staining, and MST analysis. Immunofluorescence imaging revealed that pre‐incubation with excess CTNNA1 antibody led to a dramatic reduction in EA2 binding to KYSE‐150 cells (Figure S6j, Supporting Information), providing direct evidence for competitive interaction. This observation was consistently supported by flow cytometry analysis (Figure S6k, Supporting Information), which mirrored the CTNNA1 antibody‐mediated blockade. Notably, MST analysis demonstrated a marked reduction in EA2 binding affinity (Figure S6l, Supporting Information). Upon CTNNA1 antibody treatment, the *K*_d_ values of EA2 increased markedly from 2.3 ± 0.6 µM to 299.6 ± 11.8 µM, directly evidencing antibody‐induced competitive displacement of EA2 from its binding epitope on CTNNA1. Collectively, these findings highlight a strong affinity, specificity, and in vivo targeting abilities of EA2 toward ESCC cells, providing a solid foundation for the development of targeted nanomedicine platforms for esophageal cancer.

### 2.3 Development of EA2 Aptamer‐Modified PSL for Codelivery of PTX and LUT

To circumvent PTX‐induced hepatotoxicity and enhance the synergistic effects of PTX and LUT, we designed an aptamer‐modified PSL to facilitate efficient coloading, targeted delivery, and controllable corelease of PTX and LUT within ESCC tumor/cells. The formulation of the nanocarriers included two steps (Scheme 1): preparation of PTX/LUT‐loaded PSL, followed by modification with the targeting molecule, EA2 aptamer. The nanocarrier possessed several unique features: i) DOPE and CHEMS were integrated to facilitate co‐encapsulation of hydrophobic drugs (PTX and LUT) within the lipophilic core, ensuring selective release in the acidic TME and effective endosomal escape.^[^ ^40^ ^]^ Additionally, the fusogenic properties of DOPE and CHEMS augment their interaction with the cell membrane, thereby promoting internalization.^[^ ^41^ ^]^ ii) PEGylation (addition of TPGS) was aimed at maintaining the stability of liposomes, minimizing drug leakage during blood circulation, and mitigating nonspecific interactions between liposomes and serum proteins, thereby impeding liposome clearance by the reticular endothelial system (RES).^[^ ^42^ ^]^ iii) The liposome was cationized using polyethyleneimine (PEI), which is abundant in amino groups and demonstrates the “proton sponge” effects.^[^ ^43^ ^]^ The positively charged amino groups can interact with the negatively charged phosphate groups in the EA2 aptamer through electrostatic adsorption, thereby promoting cellular internalization. iv) The EA2 aptamer, specific for the ESCC cell surface marker CTNNA1, was attached to the liposomes, facilitating the targeted codelivery of PTX and LUT.

**Figure 3:**
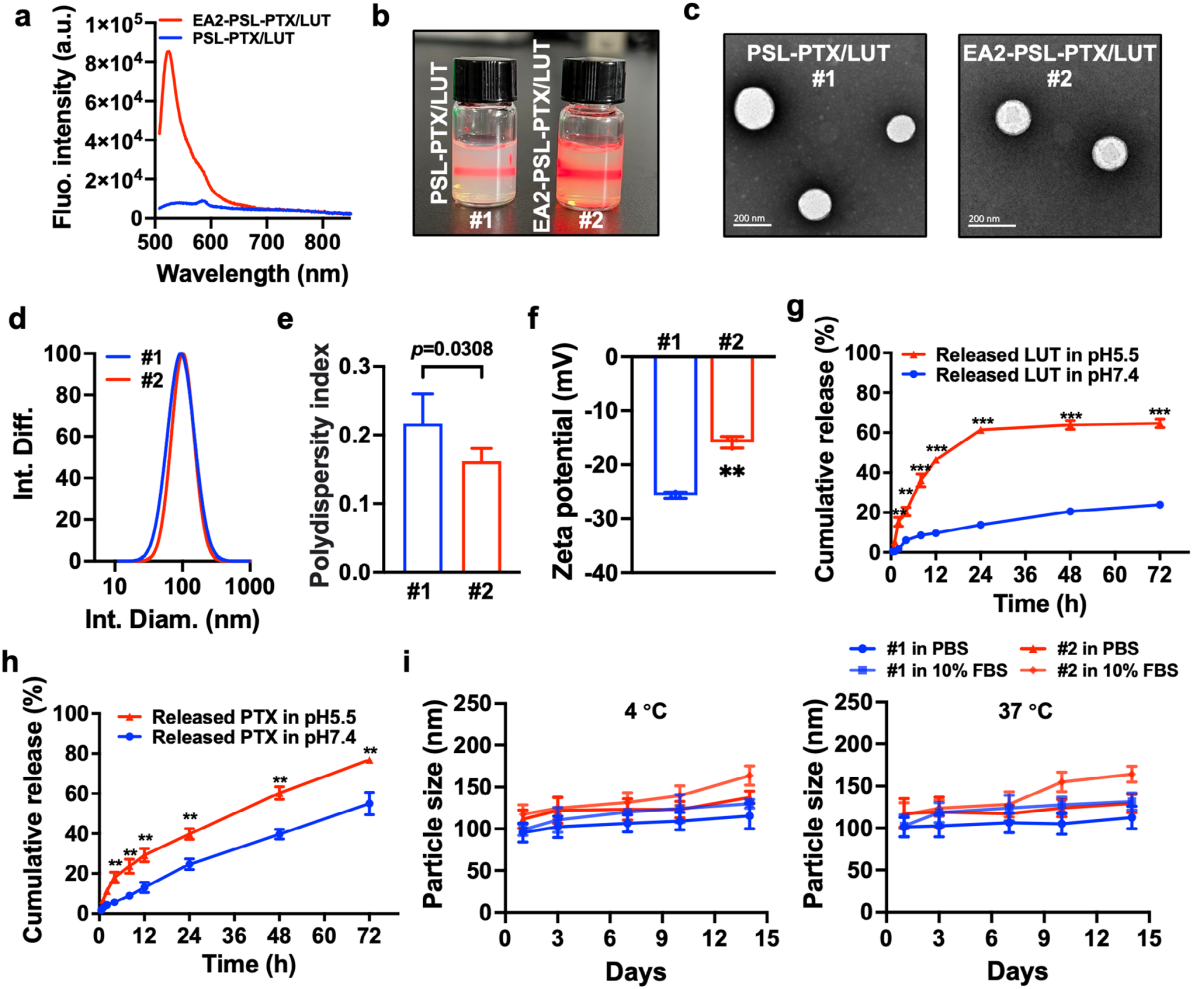
Preparation and physicochemical characterizations of EA2 aptamer‐modified and PTX/LUT‐co‐loaded pH‐sensitive liposome (EA2‐PSL‐PTX/LUT). a) FAM fluorescence emission spectra of PSL and FAM labeled‐EA2 aptamer‐modified PSL. The emission spectrum was recorded from 500 to 850 nm, with an excitation wavelength of 488 nm. b) Bright‐field appearance and Tyndall effects of PSL‐PTX/LUT (#1) and EA2‐PSL‐PTX/LUT (#2). c) Representative TEM characterization. Scale bars, 200 nm. d) Particle size distributions using DLS analysis. e‐f) Polydispersity index and surface Zeta potential. g‐h) In vitro drug release profiles of PTX and LUT from EA2‐PSL‐PTX/LUT in 0.1 M PBS (pH 7.4 and pH 5.5). i) Stability assessment of PSL‐PTX/LUT and EA2‐PSL‐PTX/LUT using mean particle size in PBS and DMEM supplemented with 10% FBS at 4 and 37 °C, respectively. All data expressed as mean ± SD (*n* = 3), statistical significance between different groups was obtained by an unpaired two‐tailed Student's t test (e, f, g, h). ^*^ *p* < 0.05, ^**^ *p* < 0.01, ^***^ *p* < 0.001, indicating statistical significance between the compared groups.

First, we investigated the effects of the proportions of lipids (DOPE and CHEMS), drug‐to‐lipid molar ratio, and TPGS addition (Figure S7 and Tables S4‐S6, Supporting Information) on the formulation. An increase in the DOPE content led to a decrease in particle size (Figure S7a,d, Supporting Information), which is attributable to the inherent structural flexibility of DOPE.^[^ ^44^ ^]^ A higher drug‐to‐lipid molar ratio apparently enhanced the vesicle organization, resulting in smaller particle sizes (Figure S7b,e, Supporting Information), albeit with a slightly decreased encapsulation efficiency (Table S5, Supporting Information). This could be attributed to the vesicle‐stabilizing properties of CHEMS and interactions of lipophilic drugs with the hydrocarbon chain.^[^ ^40^ ^]^ Moreover, the addition of TPGS to PSL improved its encapsulation efficiency (Table S6, Supporting Information) and reduced the mean particle size (Figure S7c,f, Supporting Information). The optimal molar ratio of PSL‐PTX/LUT was determined to be DOPE:CHEMS:TPGS:LUT:PTX = 6:4:0.25:0.427:0.043, with a corresponding mass ratio for 10 mL liposome of 44.64, 19.47, 3.78, 1.222, and 0.365 mg, respectively. At the optimal molar ratio, the encapsulation efficiency of PTX and LUT was 95.36 ± 1.17% and 91.78 ± 3.18%, respectively, with a drug loading capacity of 2.79 ± 0.07%. Subsequently, the results of gel electrophoresis (Figure S8a, Supporting Information) and loading capacity (Figure S8b, Supporting Information) revealed that unbound EA2 was present at the bottom of the gel. Conversely, most of the EA2 (80–250 pmol) appeared to be sequestered in the wells, and 250 pmol EA2 was chosen for effective liposome modification. Additionally, the fluorescence emission spectra (**Figure** **3a**) corroborated the characteristic peaks of FAM‐EA2‐modified PSL‐PTX/LUT observed at ≈520 nm, whereas no peak was discernible for PSL‐PTX/LUT, further indicating the successful conjugation of EA2 and PSL‐PTX/LUT.

The liposomal solutions appeared as homogeneous milky‐yellow emulsions, displaying a prominent Tyndall effect (Figure 3b). Various analytical techniques were employed to investigate the properties of the liposomes and assess the impact of aptamer modification, including TEM morphology (Figure 3c), DLS for hydrodynamic particle size (Figure 3d), polydispersity index (Figure 3e), and zeta potential analysis (Figure 3f). Both PSL‐PTX/LUT and EA2‐PSL‐PTX/LUT exhibited homogeneous and well‐defined spherical structures with a small particle size (≈100 nm), low polydispersity index (PDI) (≈0.2), and consistent and singular particle size distribution. However, in comparison to PSL‐PTX/LUT, EA2‐PSL‐PTX/LUT displayed a noticeably larger particle size (Figure S8c, Supporting Information, 127.42 ± 4.67 nm versus 102.82 ± 4.08 nm, *p* < 0.0001) and a lower PDI (0.162 ± 0.019 versus 0.217 ± 0.043, *p* < 0.05), indicating that EA2‐PSL‐PTX/LUT had a narrower range of particle size distribution. Moreover, EA2‐PSL‐PTX/LUT showed a relatively lower absolute zeta potential (–15.83 ± 1.04 versus –25.67 ± 0.57, *p* < 0.01), which could be attributed to the cationization of EA2. Particle size plays a pivotal role in the clearance of nanoparticles and influences their circulation, biodistribution of encapsulated drugs, and cytomembrane penetrability. Nanoparticles within the range of 70–200 nm can evade rapid clearance by the reticuloendothelial system (RES), resulting in prolonged in vivo circulation, enhanced tumor enrichment via EPR effects, and increased cytomembrane penetrability.

Subsequently, we investigated the pH‐responsive release of PTX and LUT from EA2‐PSL‐PTX/LUT under various conditions (pH 7.4 and pH 5.5). EA2‐PSL‐PTX/LUT displayed a pH‐triggered drug release (Figure 3g‐h). Under physiological conditions (pH 7.4), EA2‐PSL‐PTX/LUT remained relatively stable and released only minimal amounts of LUT over time. Even after 72 h, only 23.8% of LUT was released. Conversely, in an acidic environment (pH 5.5) simulating the TME, the release of LUT was notably expedited, which was attributed to the presence of DOPE and CHEMS, with a remarkable cumulative release of 64.7% within 24 h. Although acid‐accelerated release was also observed, the mechanism of PTX was relatively different, presenting a sustained release characteristic. These findings suggested that EA2‐PSL‐PTX/LUT maintained its structural integrity under neutral and physiological pH conditions with minimal drug release, whereas gradual drug release occurred as the pH transitions from 7.4 to 5.5.

To confirm the high stability of liposomes with low PDI, we assessed the long‐term storage stability (2 weeks) at 4 and 37 °C. Upon visual inspection, no nanoprecipitation, sedimentation, crystallization, or layer separation was detected during the storage of PSL‐PTX/LUT and EA2‐PSL‐PTX/LUT. Dynamic light scattering (DLS) analysis was performed to evaluate the physical stability of the liposomes by monitoring the changes in size and PDI over time. EA2‐PSL‐PTX/LUT maintained stability, with only a slight increase in size and PDI in culture medium containing 10% FBS (Figure 3i, Figure S8d, Supporting Information). This phenomenon can be ascribed to surface modification of the PEG shell of TPGS, which increased the steric hindrance between nanoparticles and provided a steric repulsion to keep sufficient stability.^[^ ^45^ ^]^ These findings underscore the robust capacity of liposomes to withstand salt‐ and serum‐induced dissociation and highlight their potential for systemic delivery.

The biocompatibility and safety of EA2‐PSL‐PTX/LUT were further investigated. The hemolysis assay (Figure S9a, Supporting Information) demonstrated that even at a high dilution of 125×, the hemolysis rate remained below 2% (Figure S9b, Supporting Information), indicating nonhemolytic behavior and ensuring excellent biosafety for intravenous administration. The biosafety of EA2‐PSL‐PTX/LUT was evaluated by intravenously injecting into mice. The physiological effects of various treatments in vivo were examined using complete blood counts. Most of blood routine data (Figure S9c‐n, Supporting Information) were within the normal range, and no obvious change was observed. Evaluation of hepatotoxicity and nephrotoxicity based on serum biochemical indexes (Figure S10a‐i, Supporting Information) further showed no significant changes in serum nitric oxide (NO), ALT, AST, AKP, total bile acid (TBA), LDH, uric acid (UA), blood urea nitrogen (BUN), and creatinine (CRE) levels between different treatments. No significant changes were observed in the coefficients of the main organs (Figure S10j, Supporting Information). Histopathological analysis of the liver and kidney tissues (Figure S10k, Supporting Information) revealed slight inflammation in the PTX and LUT combination group, whereas no morphological changes were observed in the EA2‐PSL‐PTX/LUT group, suggesting a reduction in the hepatotoxicity of free PTX due to the EA2 aptamer‐modified PSL. In addition, HE staining of the heart, spleen, lungs, and brain revealed no irregularities or lesions, indicating the safety and biocompatibility of EA2‐PSL‐PTX/LUT.

### 2.4 Enhanced Specific Cellular Internalization, Lysosomal Escape, and In Vivo ESCC Targeting Ability of EA2 Aptamer‐Modified PSL

**Figure 4:**
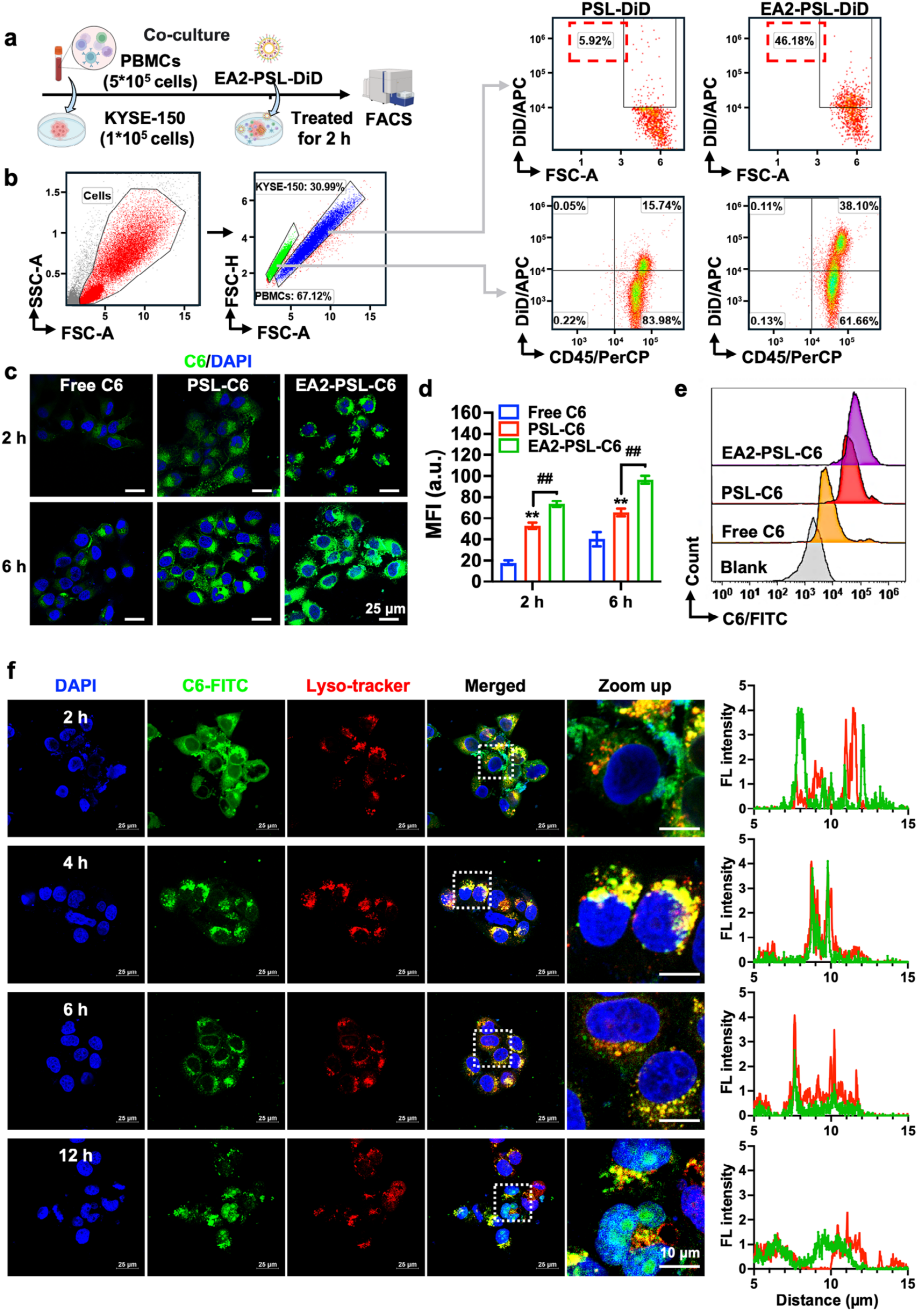
Enhanced specific cellular internalization and lysosomal escape of EA2 aptamer‐modified PSL. a) Schematic diagram of cellular uptake experiments in the coculture model of PBMCs with KYSE‐150 cells. b) Flow cytometry analysis of the cellular uptake of KYSE‐150 cells or PBMCs after incubation with PSL‐DiD or EA2‐modified PSL‐DiD for 2 h. PBMCs were labeled with PerCP anti‐human CD45 antibody. c‐d) Confocal imaging to detect the cellular internalization of C6‐loaded PSL and EA2‐PSL in KYSE‐150 cells at 2 and 6 h. Mean fluorescence intensity (MFI) was calculated by ImageJ. Nuclei were stained with DAPI. Scale bar: 25 µm. e) Flow cytometry analysis of cellular uptake at 1 h. KYSE‐150 cells were incubated with free C6, C6‐loaded PSL, and EA2‐PSL (containing equivalent amount of C6, 5 µM), and then collected for flow cytometry. f) Endocytosis and endosomal escape of C6‐loaded EA2‐PSL by observing colocalization of C6 and LysoTracker‐labeled lysosomes in KYSE‐150 cells. KYSE‐150 cells were incubated with C6‐loaded EA2‐PSL for 2, 4, 6, and 12 h at 37 °C, and lysosomes were subsequently labeled with LysoTracker Red. Nuclei were stained with DAPI. Green, red, and blue fluorescence represent C6, endo/lysosome, and nuclei, respectively. Scale bar: 25 µm. Data expressed as mean ± SD (*n* = 3), statistical significance between different groups was obtained by one‐way ANOVA using the Tukey's post‐test (d). ^**^ *p* < 0.01, significant as compared to the free C6 group. ^##^ *p* < 0.01, significant as compared to PSL‐C6 group.

To investigate whether EA2‐modified PSL could be specifically taken up by ESCC cells and could particularly be recognized and bound to CTNNA1‐overexpressing tumor cells, we prepared EA2 modified PSL‐DiD (EA2‐PSL‐DiD), and analyzed the uptake of EA2‐PSL‐DiD in various tumor cell lines using flow cytometry (Figure S11a‐b, Supporting Information). A significantly enhanced cellular uptake by KYSE‐150 cells compared to the uptake by other cancer cells, notably by those with low CTNNA1 expression (Figure S11c, Supporting Information), such as A549, HCT‐116, U251, and SiHa cells, were observed. Interestingly, increased uptake was also noted in FaDu and CAL‐27 squamous cell carcinomas with relatively higher CTNNA1 expression. Furthermore, to evaluate the targeting specificity of EA2‐modified PSL, KYSE‐150 cells were mixed with PBMCs, and then incubated with EA2‐PSL‐DiD or PSL‐DiD (**Figure** **4**a). A marked enrichment of EA2‐modified PSL‐DiD in KYSE‐150 cells was observed (Figure 4b, 46.18% versus 5.92%, an 8.9‐fold increase), whereas only a 2.4‐fold enrichment was observed in PBMCs. Similarly, specific enhanced uptake by ESCC cells (Figure S12a‐c, Supporting Information) was also observed in the coculture of myeloid‐derived suppressor cells (MDSCs) with KYSE‐150 cells. Collectively, these findings indicate that EA2‐modified PSL exhibit excellent targeting specificity to ESCC cells.

Achieving potent tumoricidal effects requires the binding of NDDS to target cells, internalization, and subsequent release of the nanomedicine within cells. The cellular uptake and lysosomal escape of liposomal nanocarriers were investigated using C6 as a fluorescent probe. The intracellular fluorescence of C6 increased over time (Figure 4c‐d) in both free drug and liposomal forms. Compared to free C6, the liposomal nanocarriers showed significantly enhanced cellular internalization. By leveraging the homing recognition between EA2 and CTNNA1, EA2 aptamer‐modified liposomes (EA2‐PSL‐C6) exhibited substantially elevated fluorescence levels. The internalization ability of the nanocarriers was further assessed quantitatively using flow cytometry (Figure 4e; Figure S11d, Supporting Information), which confirmed the superior efficacy of EA2‐PSL‐C6 delivery, as evidenced by the distinct shift in the fluorescent cells (purple curve). Subsequently, endocytosis inhibitors were used to delve deeper into the endocytosis of EA2‐PSL using confocal imaging and flow cytometry. Both the representative fluorescence images (Figure S13a, Supporting Information) and flow cytometry data (Figure S13b‐c, Supporting Information) illustrated the strong inhibitory effects of chlorpromazine, genistein, cytochalasin D, colchicine, nystatin, and wortmannin, whereas other uptake inhibitors, such as orthovanadate and chloroquine, did not affect the cellular uptake efficiency. These findings suggested that the uptake of EA2‐PSL may involve the synergistic effects of multiple internalization mechanisms, including caveolin‐mediated, cytoskeleton‐mediated, micropinocytosis, and phagocytosis‐mediated endocytic pathways.

The effective transport of nanocarriers from endo/lysosomes to the cytoplasm is essential for antitumor efficacy. We further investigated the endo/lysosomal escape of EA2‐PSL‐C6 from KYSE‐150 cells using confocal imaging. Color scatter plots were used to analyze the correlation between red and green fluorescence signals. Initially, the majority of C6 was localized to the cell membrane at 2 h (Figure 4f), with a distinct separation between the LysoTracker Red (endo/lysosome) and green (C6) signals, indicating that the membrane transport of EA2‐PSL‐C6 was facilitated by the homing recognition between EA2 and CTNNA1. After prolonged incubation, a noteworthy colocalization of C6 and endo/lysosomes was observed at 4 h, characterized by colocalized puncta emitting yellow fluorescence and strong positive correlation peaks. This observation suggested that a substantial portion of EA2‐PSL‐C6 was effectively transported via the endo/lysosomal pathways. Subsequent observations at 6 h revealed a dynamic interplay between separation and colocalization, indicating lysosomal escape. Notably, pronounced separation was observed at 12 h, indicating the effective destabilization of endo/lysosomal membranes by EA2‐PSL‐C6, thereby facilitating the entry of C6 into the cytoplasm. Moreover, as the endocytosis time increased, the intensity of green fluorescence within the cells increased, whereas the overlapping yellow fluorescence decreased. Nevertheless, a significant quantity of C6 was consistently and effectively delivered by the EA2‐PSL nanocarrier, which progressively translocated to the nucleus within 12 h.

**Figure 5:**
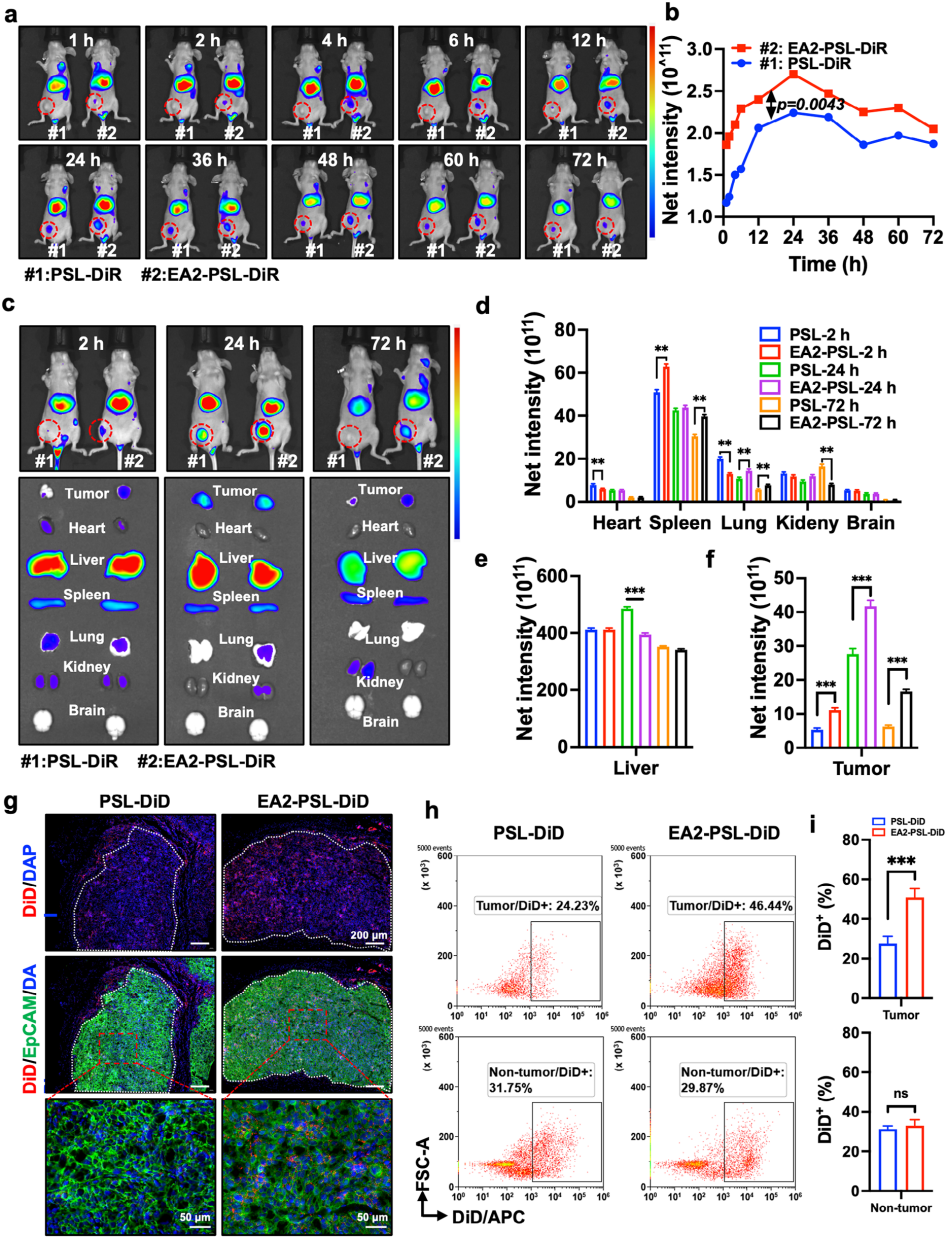
Tumor targeting and penetration of EA2 aptamer‐modified PSL in vivo. a) Representative fluorescence images of KYSE‐150 tumor‐bearing mice at different time intervals post‐intravenous injection with DiR‐loaded PSL and EA2‐PSL. The tumor regions are delineated by red dashed circles. b) Fluorescence intensity analysis and pharmacokinetic profiles of accumulated DiR at tumor sites. c‐f) *Ex vivo* imaging and biodistribution of tumor tissue and major organs at 2, 24, and 72 h post‐intravenous injection with DiR‐loaded PSL and EA2‐PSL. BALB/c nude mice, inoculated with 5 × 10^6^ cells KYSE‐150 cells, were intravenously injected with DiR‐loaded PSL and EA2‐PSL (at equivalent amount of DiR, 1 µg). Subsequently, fluorescence images were captured and quantified at 1, 2, 4, 6, 12, 24, 36, 48, 60, and 72 h. For *ex vivo* imaging, mice were euthanized at 2, 24, and 72 h after injection, and their main organs were collected for live imaging scanning. g) The representative immunofluorescence images showing the tumor penetration after 24‐h intravenous injection of DiD‐loaded PSL and EA2‐PSL, as examined by confocal microscopy. Nanoparticles were indicated by DiD (red), tumor cells were depicted in green. Scale bar: 200 µm. h‐i) Flow cytometry analysis investigating the biodistribution of nanoparticles in tumors (tumor and non‐tumor cells) after injection of DiD‐loaded PSL or EA2‐PSL for 24 h. All data expressed as mean ± SD (*n* = 3), statistical significance between different groups was obtained by an unpaired two‐tailed Student's t test (**b, d, e, f, i**) ^**^ *p* < 0.01, ^***^ *p* < 0.001, significant as compared to the PSL group.

After intravenous injection, biodistribution of nanoparticles in the blood circulation might influence the targeting of EA2‐modified PSL and cause off‐target toxicities. We performed biodistribution studies using blood cells (Figure S14, Supporting Information), which further revealed that the incubation time did not significantly affect the uptake of EA2‐modified PSL, facilitating longer circulation time in the blood. Moreover, EA2 modification significantly enhanced the specific ability to kill KYSE‐150 cells (Figure S15, Supporting Information), whereas only a slight increase was observed for PBMCs. These findings indicated that EA2‐modified PSL could specifically target ESCC cells and exhibit low off‐target toxicities. Furthermore, the in vivo tumor‐targeting ability of the EA2‐modified PSL was investigated in tumor‐bearing mice. The DiR fluorescence intensity at the tumor site increased within the initial 24 h (**Figure** **5**a‐b), indicating sustained and continuous accumulation of nanocarriers. Maximum DiR accumulation in the tumors was observed at 24 h for both PSL‐DiR and EA2‐PSL‐DiR, with the fluorescence intensity of EA2‐PSL‐DiR being 1.2‐times greater than that of PSL‐DiR. Compared with PSL‐DiR, EA2‐PSL‐DiR exhibited remarkably higher tumor accumulation at each time point (*p* = 0.0043). The fluorescence intensities within the tumors decreased over time owing to degradation and clearance of the nanocarriers. However, PEGylation extended the circulation time of the nanocarriers, enabling the detection of fluorescence intensities within the tumors even at 72 h. The highest and preferential tumor accumulation of EA2‐PSL‐DiR was confirmed by *ex vivo* images (Figure 5c‐f), and the relative fluorescence intensity of EA2‐PSL‐DiR in the tumors was approximately 2.1‐, 1.5‐, and 2.6‐fold higher than that of PSL‐DiR at 2, 24, and 72 h (Figure 5f), respectively, indicating enhanced tumor‐targeting efficiency. The high tumor accumulation of EA2‐PSL‐DiR can be attributed to the synergistic effects of the active targeting properties of the EA2 aptamer, pH‐sensitive release, slow blood clearance, and EPR effects of the nanocarrier. Additionally, a notable reduction in liver fluorescence was observed (Figure 5e), suggesting that the delivery system effectively reduced hepatotoxicity.

The enhanced tumor penetration and specific targeting abilities of EA2‐PSL were further elucidated by observing frozen tumor sections. Tumor cells were stained with FITC‐labeled anti‐EpCAM antibody. As illustrated in Figure 5g, a markedly high red fluorescence signal was detected in the EA2‐PSL‐DiD group, which correlated well with the in vivo imaging results. A more diffusive fluorescence pattern was noted in the EA2‐PSL‐DiD group, whereas the PSL‐DiD group showed red fluorescence predominantly concentrated in the peripheral area of the tumor nest. Additionally, the increased red fluorescence signal within tumor cells further demonstrated the specific targeting efficacy of EA2‐PSL toward tumor cells. These findings indicate that EA2‐PSL exhibits a favorable tumor penetration. We further characterized the distribution of PSLs in tumors, including in tumor cells (CD45^−^/EpCAM^+^), stromal cells (CD45^−^/EpCAM^−^), and immunocytes (CD45^+^/EpCAM^−^) using flow cytometry (Figure 5h; Figure S16, Supporting Information). PSLs exhibited preferential distribution within tumor cells, with their accumulation being inversely correlated with EA2 modification. Compared to the PSL group, the EA2‐PSL group demonstrated a significantly enhanced capacity to enter tumor cells (Figure 5i), suggesting that EA2 modification substantially increases the uptake of PSLs in tumor cells. These findings collectively indicate the specific capacity of EA2 aptamer‐modified PSL to efficiently and gradually deliver chemotherapeutic agents to ESCC cells.

### 2.5 In Vitro Synergistic Cancer Suppression Efficiency Enhancement by EA2 Aptamer‐Modified PSL

**Figure 6:**
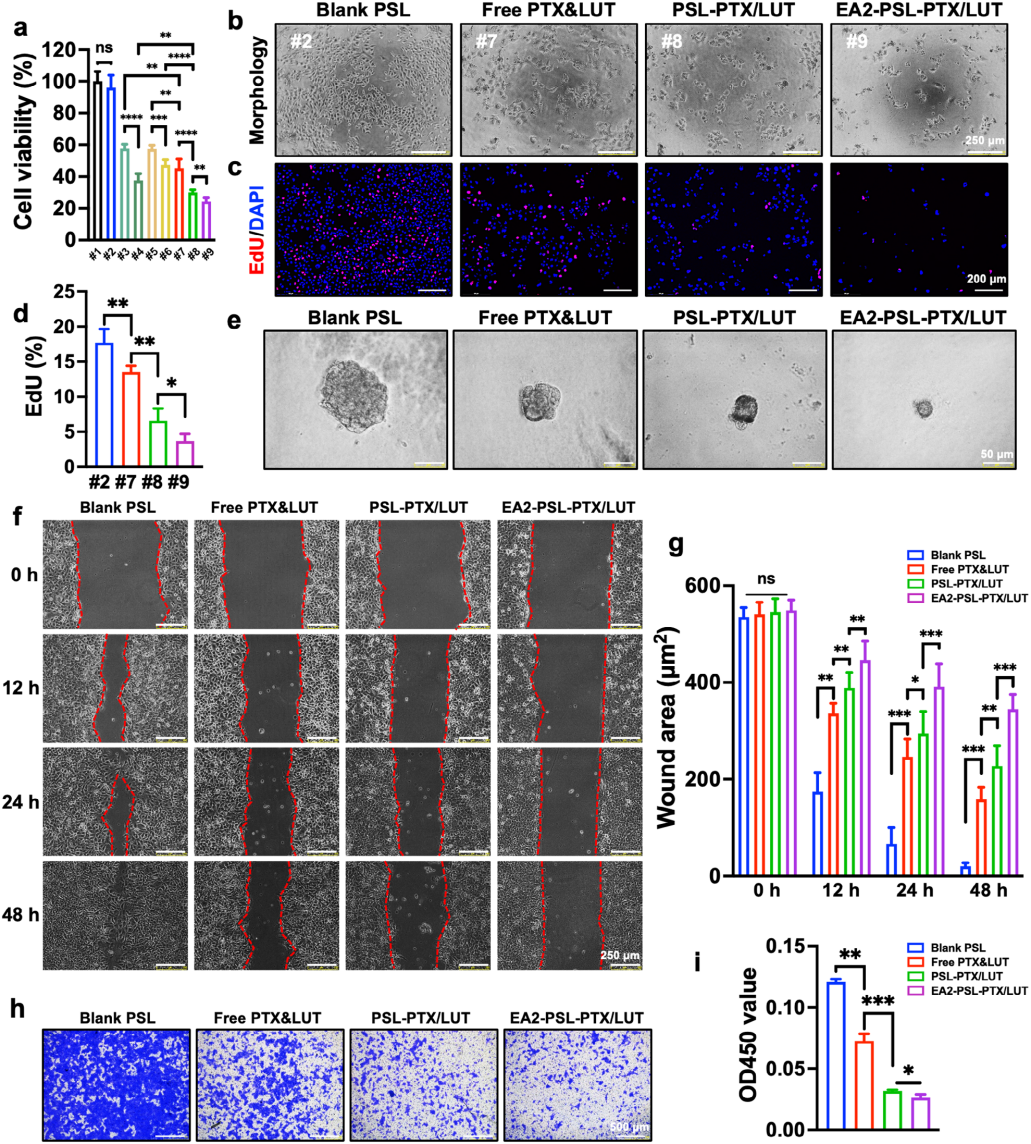
In vitro synergistic cancer suppression efficiency of EA2 aptamer‐modified PSL in KYSE‐150 cells. a) Cell viabilities of KYSE‐150 cells treated with free drug or different NDDS for 48 h using CCK‐8 assay, treatments include PBS (#1), blank PSL (#2), PTX (#3), PTX‐loaded PSL (#4), LUT (#5), LUT‐loaded PSL (#6), free PTX+LUT (#7), PSL‐PTX/LUT (#8), and EA2‐PSL‐PTX/LUT (#9) at equimolar doses of PTX (7.5 nM) and LUT (15 µM). b) Representative morphological changes. Cells were treated with free drug or different NDDS for 24 h, and images were captured by inverted optic microscope. Scale bars, 250 µm. c‐d) The suppression effects of different NDDS on proliferation of KYSE‐150 cells by EdU assay. Scale bar, 200 µm. e) 3D tumor spheroid images of the KYSE‐150 cells showing the anticancer effects of various NDDS. Scale bars, 50 µm. f‐g) Suppression effects of free drug or different NDDS on migration abilities of KYSE‐150 cells using wound healing assay. Representative images were captured at 0, 12, 24, and 48 h. Scale bars, 250 µm. The wound area was analyzed by ImageJ. h‐i) Inhibition effects of free drug or different NDDS on invasion abilities of KYSE‐150 cells using a Transwell assay. Invaded cells were fixed and stained with crystal violet, and images were captured by inverted optic microscope. Cells were destained using DMSO for detecting OD value at 570 nm. Scale bars, 500 µm. All data expressed as mean ± SD (*n* = 3), statistical significance between different groups was obtained by one‐way ANOVA using the Tukey's post‐test (a, d, g, i). ^*^ *p* < 0.05, ^**^ *p* < 0.01, ^***^ *p* < 0.001, indicating statistical significance between the compared groups.

After successfully developing a coloaded delivery system for PTX and LUT, which incorporated aptamer‐mediated ESCC‐targeted binding and exhibited acid‐triggered drug release characteristics, we evaluated the inhibitory efficacy of EA2‐PSL‐PTX/LUT in KYSE‐150 cells. The free drugs (PTX and/or LUT) and their single‐drug‐loaded PSL counterparts (PSL‐PTX and PSL‐LUT) were used as controls. The cytotoxicity of free drugs and various NDDS was investigated using the CCK‐8 assay. Blank PSL exhibited negligible toxicity in KYSE‐150 cells, underscoring the biosafety of this nanocarrier. In contrast, cell viability was significantly reduced following treatment with the drug‐loaded nanocarriers (**Figure** **6a**). Consistent with the aforementioned findings, the PTX and LUT combination displayed heightened synergistic cytotoxicity compared with the individual free drugs, whereas the PSL carrier markedly enhanced their cytotoxicity. Notably, EA2‐PSL‐PTX/LUT exhibited the highest cytotoxicity toward KYSE‐150 cells, which was attributed to the strong binding specificity of the EA2 aptamer to CTNNA1‐overexpressing ESCC cells and increased cellular uptake of the drugs. The suppression efficiency of EA2‐PSL‐PTX/LUT was evaluated based on cell morphology, colony formation, EdU, and 3D spheroid growth assays. Distinct alterations in cell morphology were observed in the PSL‐PTX/LUT group (Figure 6b; Figure S17a, Supporting Information), characterized by reduced cell numbers, a shrunken and rounded appearance, enlarged cytoplasmic vacuoles, extended pseudopods, and disorganized arrangement. These morphological changes were more pronounced in the EA2‐PSL‐PTX/LUT group. Additionally, colony formation (Figure S17b, Supporting Information) and EdU assay results (Figure 6c‐d; Figure S17c‐d, Supporting Information) demonstrated reduced colony size and proliferation rates in the NDDS‐treated groups, with EA2‐PSL‐PTX/LUT displaying superior antiproliferative effects compared to PSL‐PTX/LUT. Although 2D cellular models provide insights into cell–particle interactions, they lack the complexity of the 3D environment. To further validate the cytotoxicity of EA2‐PSL‐PTX/LUT, its spatial and temporal efficacy was evaluated in 3D KYSE‐150 spheroids. EA2‐PSL‐PTX/LUT potently inhibited spheroid growth (Figure 6e; Figure S17e‐f, Supporting Information), surpassing the limited effects of free PTX or LUT, and PSL‐PTX/LUT. Moreover, wound‐healing (Figure 6f‐g; Figure S17g‐h, Supporting Information) and Transwell assays (Figure 6h‐i; Figure S17i‐j, Supporting Information) revealed that EA2‐PSL‐PTX/LUT exhibited enhanced inhibition of cell migration and invasion compared to PTX and LUT, as evidenced by larger wound areas and reduced number of invading cells. These findings underscore the potential of EA2 aptamer‐modified multifunctional carriers in delivering PTX and LUT intracellularly, thereby enhancing their synergistic antitumor effects.

### 2.6 In Vivo Antitumor Efficacy of EA2‐PSL‐PTX/LUT

**Figure 7:**
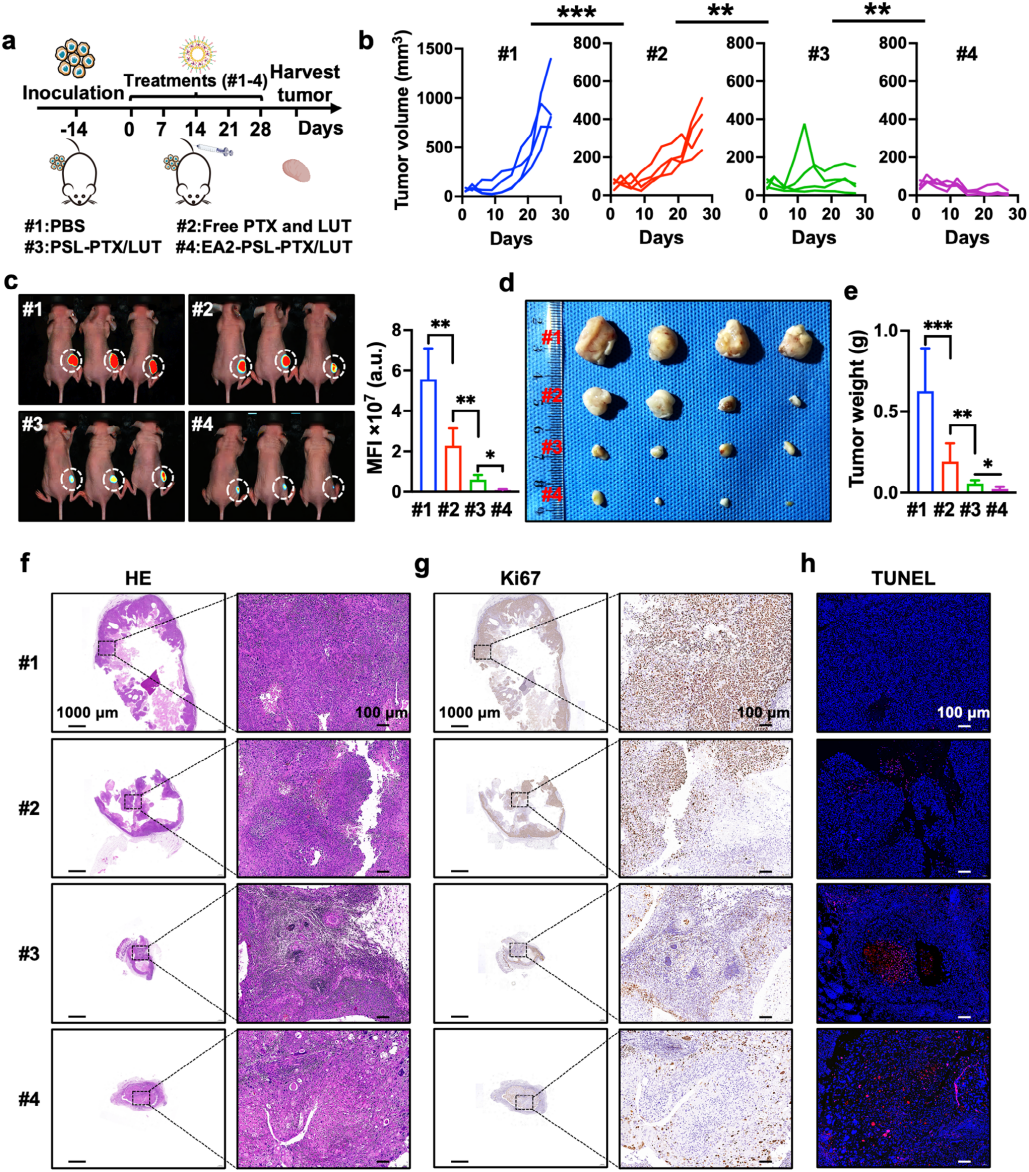
In vivo anti‐tumor efficacy of EA2‐PSL‐PTX/LUT. a) Schematic diagram of therapeutic strategy with PBS (#1), Free PTX and LUT (#2), PSL‐PTX/LUT (#3), and EA2‐PSL‐PTX/LUT (#4). b) Individual tumor growth curves of KYSE‐150 tumor‐bearing mice with different treatments. c) Luciferase signals of ESCC tumor burden were imaged by bioluminescence after different treatments administered five times. Mean luminescence levels of ESCC tumors in mice were calculated. d‐e) Tumor photographs and tumor weights of isolated tumor tissues. f‐h) Representative micrographs of HE, Ki67, and TUNEL staining of tumor tissue in each group. Luciferase‐expressing KYSE‐150 cells were subcutaneously inoculated into BALB/c nude mice (5×10^6^/100 µL) to establish a subcutaneous xenograft tumor model. Various treatments were intravenously injected into mice at equimolar doses of PTX (2.5 mg kg^−1^) and LUT (10 mg kg^−1^). Treatments were given every 7 days for a total of five cycles. All data expressed as mean ± SD (*n* = 4), statistical significance between different groups was obtained by one‐way ANOVA using the Tukey's post‐test (b, c, e). ^*^ *p* < 0.05, ^**^ *p* < 0.01, ^***^ *p* < 0.001, indicating statistical significance between the compared groups.

The in vivo antitumor effects of the liposomal carriers were evaluated in a mouse model subcutaneously inoculated with KYSE‐150 cells (**Figure** **7a**). No noticeable behavioral or mental abnormalities were observed throughout the treatment period. The minimal fluctuations in body weight among all the groups over time (Figure S18a, Supporting Information) indicated the safety and biocompatibility of the nanocarriers. Notably, the tumor growth curves (Figure 7b; Figure S18b, Supporting Information) confirmed superior tumor growth inhibition by EA2‐PSL‐PTX/LUT, whereas the tumor volume steadily increased over time in the PBS and PTX and LUT combination groups. Correspondingly, bioluminescence imaging, performed to capture the ESCC tumor burden after treatment with nanocarriers (Figure 7c), revealed noticeable tumor growth inhibition and delayed progression in all NDDS‐treated mice. PSL‐PTX/LUT exhibited a significant reduction in tumor burden compared to the PTX and LUT combination group (2.28 ± 0.87 ×10^7^ a.u. versus 0.60 ± 0.22 ×10^7^ a.u., *p* < 0.01). EA2‐PSL‐PTX/LUT demonstrated the most profound suppression of tumor growth, displaying the lowest tumor burden (0.084 ± 0.037 ×10^7^ a.u.). The remarkable cancer suppression efficacy of PSL can be attributed to several factors: (i) prolonged circulation of PSL prevents direct contact with the plasma, minimizes drug leakage during circulation, and reduces nonspecific interactions with serum proteins; (ii) selective release of PSL into the acidic TME and efficient endosomal escape; (iii) enhanced cellular internalization of EA2‐PSL‐PTX/LUT through EPR effects and specific binding of the EA2 aptamer to ESCC cells. Following treatment, tumor tissues were excised and collected (Figure 7d) for measuring the tumor weight (Figure 7e), which revealed superior therapeutic efficacy of EA2‐PSL‐PTX/LUT. Furthermore, a comparison of the HE staining results of the tumors (Figure 7f) showed notable necrosis of tumor cells in the PSL groups, with the most severe cellular changes (condensed nuclei, reduced cytoplasmic and nuclear staining, cytoplasmic vacuolization, and cell shrinkage) observed in the EA2‐PSL‐PTX/LUT group, indicating its superior tumor‐suppressive efficacy. The Ki67 assay (Figure 7g) showed a decrease in the brown‐stained area, whereas the TUNEL assay (Figure 7h) showed an increase in the red fluorescence signal, indicating that EA2‐PSL‐PTX/LUT promoted cell apoptosis and inhibited cell proliferation in vivo. Collectively, EA2‐PSL offers significant advantages in targeted delivery of PTX and LUT for enhanced synergistic suppression of ESCC.

### 2.7 EA2‐PSL‐PTX/LUT Inhibited Tumor Growth by Remodeling the Immunosuppressive Tumor Microenvironment

**Figure 8:**
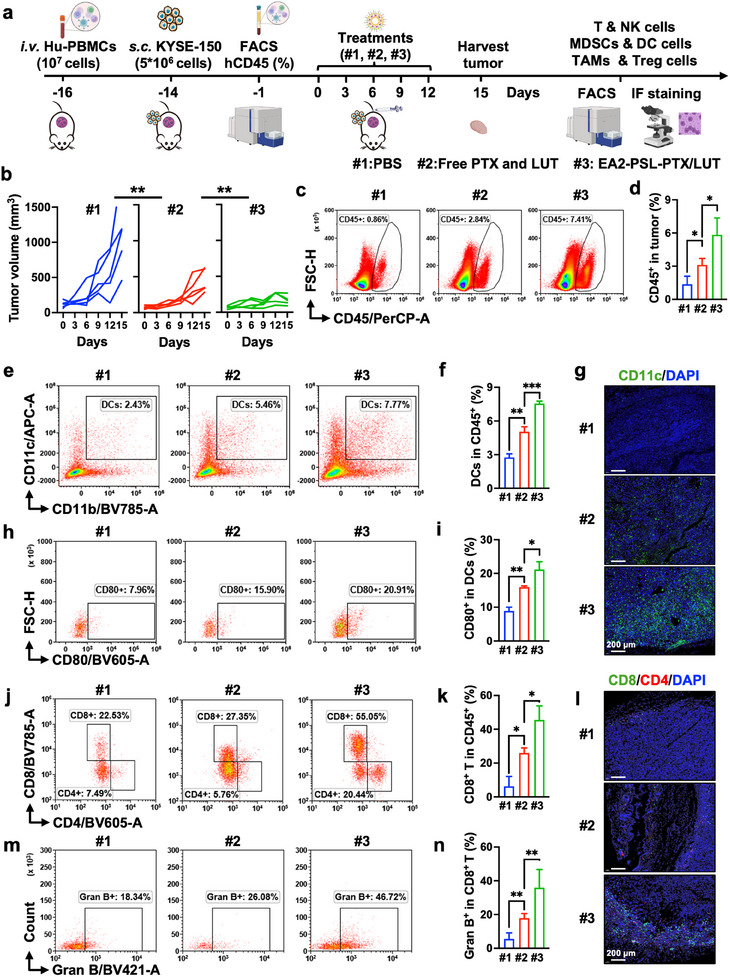
EA2‐PSL‐PTX/LUT increases intratumoral accumulation of DCs and CD8^+^ T cells. a) Schematic diagram of therapeutic strategy with PBS (#1), free PTX and LUT (#2), and EA2‐PSL‐PTX/LUT (#3) at equimolar doses of PTX (2.5 mg kg^−1^) and LUT (10 mg kg^−1^) in the PBMCs‐engrafted tumor‐bearing mice model. b) Individual tumor growth curves of KYSE‐150 tumor‐bearing mice with different treatments. c‐d) Representative flow cytometric analysis and proportion of immune cells (CD45^+^) in tumors. e‐f) Representative flow cytometric analysis and proportion of DCs (CD11b^+^/CD11c^+^) in CD45^+^ cells. g) Representative immunofluorescence images showing tumor‐infiltrating DCs, as examined by confocal microscopy. Blue: DAPI; Green: CD11c; Scale bar, 200 µm. h‐i) Representative flow cytometric analysis and proportion of matured DCs (CD80^+^/CD11c^+^). j) Representative flow cytometric analysis for CD8^+^ and CD4^+^ T cells in tumors. k) Quantitative analysis of CD8^+^ T cells in CD45^+^ cells. l) Representative immunofluorescence images showing tumor‐infiltrating T cells, Blue: DAPI; Green: CD8^+^ T; Red: CD4^+^ T; Scale bar, 200 µm. m‐n) Representative flow cytometric analysis for the expression of the cytotoxicity markers Granzyme B in tumor‐infiltrating CD8^+^ T cells. For tumor growth curves, data expressed as mean ± SD (*n* = 5); for flow cytometry, data expressed as mean ± SD (*n* = 3). Statistical significance between different groups was obtained by one‐way ANOVA using the Tukey's post‐test (b, d, f, i, k, n). ^*^ *p* < 0.05, ^**^ *p* < 0.01, ^***^ *p* < 0.001, indicating statistical significance between the compared groups.

Next, we investigated whether EA2‐PSL‐PTX/LUT inhibited tumor growth by altering the immunosuppressive TME. The capacity of EA2‐PSL‐PTX/LUT to rebuild the immunosuppressive microenvironment within ESCC tumors was evaluated in a PBMCs‐engrafted KYSE‐150 tumor‐bearing mouse model (**Figure** **8a**). The percentage of human CD45^+^ cells was evaluated in mouse peripheral blood after 15 days of injecting PBMCs. The proportion of human CD45^+^ cells exceeded 20% (Figure S19a‐b, Supporting Information), confirming successful establishment of the model. During treatment, the body weights of the mice gradually increased without significant changes among the groups (Figure S19c, Supporting Information). Both PTX and LUT combination and EA2‐PSL‐PTX/LUT significantly inhibited the tumor growth (Figure 8b; Figure S19d, Supporting Information). The EA2‐PSL‐PTX/LUT group exhibited the greatest antitumor efficiency, consistent with the observed superiority of the antitumor activity in the nude model.

To further investigate the mechanisms of EA2‐PSL‐PTX/LUT for immunochemotherapy, we analyzed the alterations in the immune profiles within TME after administration using flow cytometry (Figure S20, Supporting Information). Tumors treated with EA2‐PSL‐PTX/LUT had significantly higher levels of CD45^+^ immune cells than those treated with PBS or free PTX and LUT (Figure 8c‐d), highlighting the increased recruitment of immune cells to the tumor microenvironment by EA2‐PSL‐PTX/LUT. To further validate the recruitment of immune cells, we employed a Transwell co‐culture system to mimic the ESCC tumor microenvironment under NDDS treatment condition (Figure S21a, Supporting Information). Confocal microscopy analysis (Figure S21b‐c, Supporting Information) revealed that activation of CTNNA1 signaling significantly enhances immune cell recruitment. Intriguingly, non‐modified NDDS demonstrated dual functionality: while inducing cytotoxic effects on KYSE‐150 cells, they concurrently recruited immune cells at levels significantly higher than the EA2 control group. Furthermore, the EA2‐modified NDDS (EA2‐PSL‐PTX/LUT) achieved a 1.5‐fold increase in PBMC infiltration compared to the unmodified PSL‐PTX/LUT formulation.

Mature dendritic cells (DCs), as the primary antigen presenting cells, are important in initiating both innate and adaptive immune responses. The presence of MDSCs and regulatory T cells (Tregs) can inhibit the maturation of DCs, thereby impairing their ability to secrete essential costimulatory molecules and cytokine signals required for T‐cell activation. Therefore, the maturation of DCs within the tumor was investigated. Both flow cytometry (Figure 8e‐f) and immunofluorescence analysis (Figure 8g) revealed that EA2‐PSL‐PTX/LUT considerably increased the infiltration of DCs into the tumor. Compared with the PBS group, the maturation of DCs was notably increased to approximately 20% in the EA2‐PSL‐PTX/LUT‐treated group (Figure 8h‐i), which was essential for promoting the priming and recruitment of T cells. Subsequently, mature DCs present antigens to T cells and induce their activation. Therefore, T cells were further analyzed. As shown in Figure 8j‐l, the total number of tumor‐infiltrating CD8^+^ T cells was significantly increased after the treatments. Compared to PBS or free PTX and LUT, EA2‐PSL‐PTX/LUT markedly increased the infiltration (Figure 8k) and granzyme B production (Figure 8m‐n) in specific cytotoxic CD8^+^ T cells within the TME. These results demonstrate that EA2‐PSL‐PTX/LUT can effectively promote the infiltration and maturation of DCs, increase intra‐tumoral T‐cell recruitment, and boost antitumor CD8^+^ T‐cell immunity within the TME.

**Figure 9:**
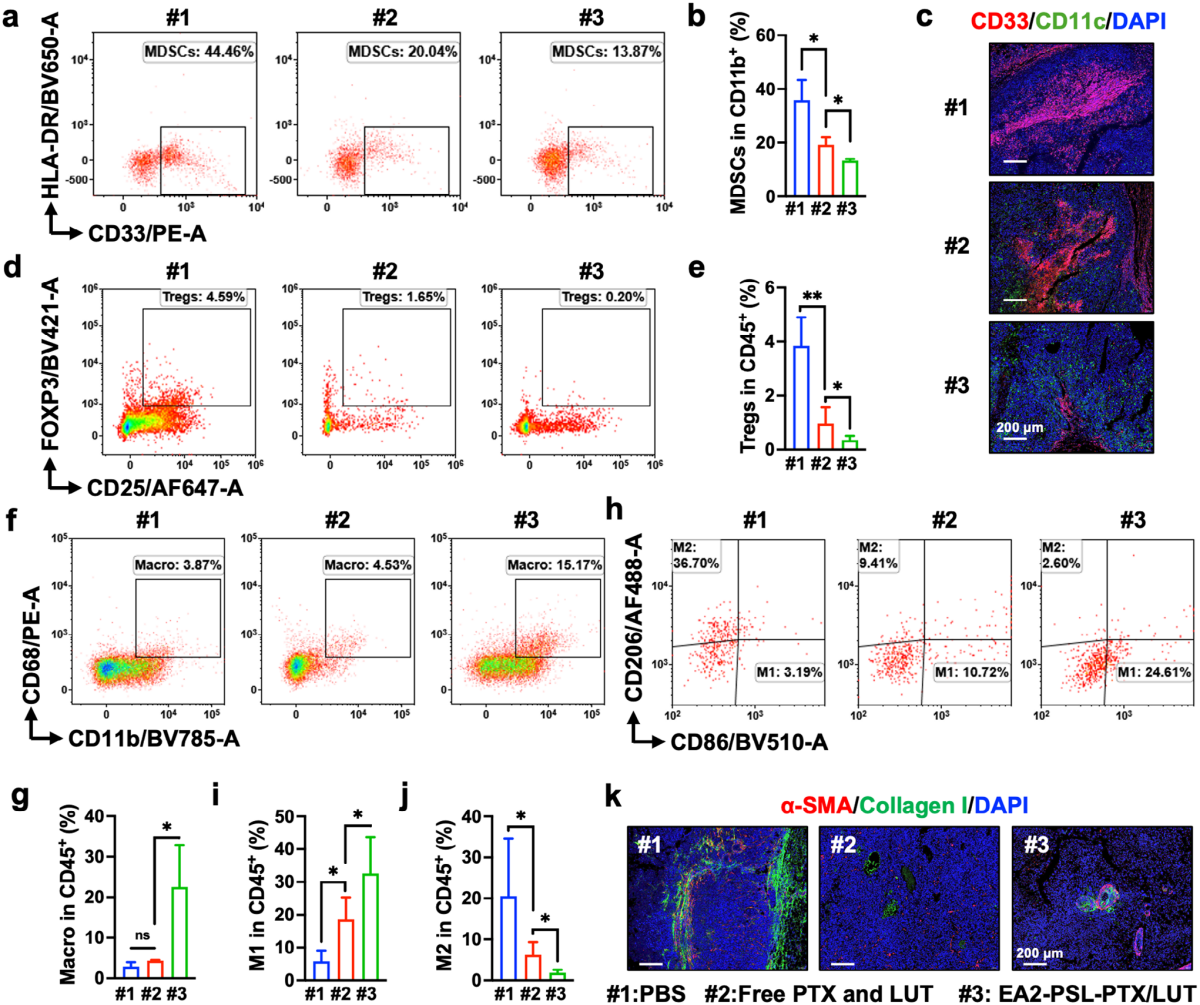
EA2‐PSL‐PTX/LUT remodels the immunosuppressive tumor microenvironment. a‐b) Representative flow cytometric analysis and proportion of MDSCs (CD11b^+^/CD33^+^/HLA‐DR^−^) in myeloid cells. #1: PBS, #2: Free PTX and LUT, #3: EA2‐PSL‐PTX/LUT at equimolar doses of PTX (2.5 mg kg^−1^) and LUT (10 mg kg^−1^). c) Representative immunofluorescence images showing the distribution of MDSCs in tumors, as examined by confocal microscopy. Blue: DAPI; Red: CD33; Green: CD11c; Scale bar, 200 µm. d‐e) Representative flow cytometric analysis and proportion of Tregs (CD4^+^/CD25^+^/FOXP3^+^) in CD45^+^ cells. f‐g) Representative flow cytometric analysis and proportion of TAMs (CD11b^+^/CD68^+^) in CD45^+^ cells. h‐j) Representative flow cytometric analysis and proportion of M1 (CD86^+^/CD206^−^) and M2 (CD206^+^/CD86^−^) TAMs in CD45^+^ cells. k) Representative immunofluorescence images showing the expression of α‐SMA and collagen deposition in tumors. Blue: DAPI; Red: α‐SMA; Green: collagen I; Scale bar, 200 µm. All data expressed as mean ± SD (*n* = 3), statistical significance between different groups was obtained by one‐way ANOVA using the Tukey's post‐test (b, e, g, i, j). ^*^ *p* < 0.05, ^**^ *p* < 0.01, indicating statistical significance between the compared groups.

The immunosuppressive TME has the potential to undermine therapeutic efficacy. Specifically, during ESCC development, the abundance of MDSCs increases progressively, creating an immunosuppressive microenvironment, while the proportion of antitumor CD8⁺ T cells declines in parallel. Hence, we analyzed the immunosuppressive cells, including MDSCs, Tregs, and tumor‐associated macrophages (TAMs), in tumors. A significantly higher percentage of MDSCs was observed in the PBS group (**Figure** **9**a‐c). EA2‐PSL‐PTX/LUT treatment substantially blocked the infiltration of MDSCs. Additionally, flow cytometry data showed that EA2‐PSL‐PTX/LUT considerably depleted Tregs (Figure 9d‐e). TAMs are the main executors that induce phagocytosis of tumor cells. Hence, we further clarified the expression of M1‐type (CD86) and M2‐type (CD206) TAMs in tumors after treatments. Flow cytometry data (Figure 9f‐j) showed that the proportion of CD86^+^ cells was significantly increased, whereas that of CD206^+^ cells was decreased in tumors treated with EA2‐PSL‐PTX/LUT compared with that in tumors treated with PBS or free PTX and LUT. These findings suggested that EA2‐PSL‐PTX/LUT significantly promoted the polarization of TAMs toward the M1‐type. These results collectively indicate that the immunosuppressive TME was dramatically reversed by EA2‐PSL‐PTX/LUT treatment.

The antitumor immunity conferred by the infiltration of immune cells is significantly impeded by physical barriers in tumors. For example, activated cancer‐associated fibroblasts (CAFs) and secreted collagen I can prevent T cells from entering tumors, thereby enabling tumor cells to evade immune surveillance. We investigated the physical barriers (characterized by collagen I) and activated CAFs (characterized by α‐SMA) in the tumors. Diffuse intracytoplasmic α‐SMA and collagen deposition was abundant in the PBS group, whereas the EA2‐PSL‐PTX/LUT treatment notably reduced the α‐SMA‐positive CAFs and collagen in the tumors (Figure 9k). These findings suggested that EA2‐PSL‐PTX/LUT alleviated the stromal barrier by deactivating the CAFs and reducing the collagen deposition. Taken together, EA2‐PSL‐PTX/LUT reeducated the TME into an immune‐activated state of ESCC tumors by promoting maturation of dendritic cells, infiltration of T cells, and M1‐type polarization of TAMs. Additionally, the immunosuppressive microenvironment was remarkably remodeled by decreasing the MDSC and Treg infiltration and softening of the stromal barrier.

Despite preclinical promise, translating EA2‐PSL‐PTX/LUT to the clinic still requires addressing critical challenges, such as batch‐to‐batch consistency, storage stability, and formulation standardization. Notwithstanding these hurdles, the translational potential of our nanocarrier is underscored by its dual functionality—targeted drug co‐delivery and TME remodeling, which addresses unmet needs in ESCC therapy. Moving forward, integrating pharmacokinetic modeling and conducting toxicity studies (including cardiovascular, hepatic and renal safety) will bridge the gap between preclinical and clinical. While this study establishes a foundational framework, interdisciplinary collaborations are needed to refine manufacturing processes, enhance formulation stability, and validate safety profiles, thereby advancing aptamer‐targeted liposomes toward clinical viability in ESCC therapy.

## 3 Conclusions

In conclusion, the synergistic inhibitory effects of LUT and PTX on ESCC, with reduced hepatotoxicity, are noteworthy, and this study aimed to develop an effective NDDS to capitalize on them. The selected aptamer, EA2, demonstrated a high specificity and binding affinity for CTNNA1 in ESCC cells. A pH‐sensitive, aptamer‐modified, multifunctional liposomal drug delivery platform (EA2‐PSL‐PTX/LUT) was successfully engineered for targeted combination chemotherapy. This nanocarrier shows immense promise for achieving efficient coloading, precise targeting, and controlled corelease of PTX and LUT. It enhances cellular uptake, prolongs the circulation time, and increases the accumulation of drugs in malignant tissues. In both in vitro and in vivo studies, EA2‐PSL‐PTX/LUT exhibited significantly enhanced antitumor efficacy against ESCC. Notably, EA2‐PSL‐PTX/LUT was confirmed to remarkably reeducate the immunosuppressive TME of ESCC tumors into an immune‐activated state by promoting the maturation of dendritic cells, infiltration of T cells, M1‐type polarization of TAMs, and decreasing MDSC and Treg infiltration. These results validate our hypothesis that pH‐responsive and aptamer‐modified multifunctional liposomes hold considerable potential as nanocarriers for the precise delivery of combination chemotherapeutic agents for ESCC targeted therapy.

## 4 Experimental Section

### Animals Experiments

C57BL/6 mice (SPF, female, 8 weeks, license No. A202308080054), BALB/c nude mice (SPF, female, 4–5 weeks, license No. A202308220078) and NCG mice *(Prkdc)KO/KO, (Il2rg)KO/KO* (SPF, female, 5 weeks, license No. A202412100332) were purchased from GemPharmatech Co., Ltd. (Nanjing, China). All animals were housed in a specific pathogen‐free facility in microisolator cages with a 12/12 h light/dark cycle, maintained at ambient temperature of 20–26 °C, and relative humidity of 40–70%. Animals were provided with autoclaved food and had access to acidified autoclaved water. All animal procedures were approved by the Animal Care and Use Committee of the Affiliated Huai'an No. 1 People's Hospital of the Nanjing Medical University (DW‐P‐2024‐008‐01).

### Statistical Analysis

The mean ± standard deviation was used to represent the acquired experimental data. GraphPad Prism 10.0 (GraphPad Software, USA) was used to draw all graphs and conduct statistical analyses. Comparisons between two groups were performed using an unpaired two‐tailed Student's t test. Through a one‐way analysis of variance (ANOVA) coupled with the Tukey's post hoc test, the statistical significance of differences among diverse groups was evaluated. Significance levels (*p* ˂ 0.05, *p* ˂ 0.01, *p* ˂ 0.001, and *p* ˂ 0.0001) were deemed statistically acceptable.

Details of reagents, antibodies, and methods used are provided in the Supporting Information.

## Conflict of Interest

The authors declare no conflict of interest.

## Supporting information



Supporting Information

## Data Availability

The data that support the findings of this study are available from the corresponding author upon reasonable request.

## References

[1] F. Bray , M. Laversanne , H. Sung , J. Ferlay , R. L. Siegel , I. Soerjomataram , A. Jemal , CA Cancer. J. Clin. 2024, 74, 229. 38572751 10.3322/caac.21834

[2] E. Morgan , I. Soerjomataram , H. Rumgay , H. G. Coleman , A. P. Thrift , J. Vignat , M. Laversanne , J. Ferlay , M. Arnold , Gastroenterology 2022, 163, 649. 35671803 10.1053/j.gastro.2022.05.054

[3] C. Xia , P. Basu , B. S. Kramer , H. Li , C. Qu , X. Q. Yu , K. Canfell , Y. Qiao , B. K. Armstrong , W. Chen , Lancet Public Health 2023, 8, 996. 10.1016/S2468-2667(23)00186-X PMC10665203 38000379

[4] R. Chen , R. Zheng , S. Zhang , S. Wang , K. Sun , H. Zeng , L. Li , W. Wei , J. He , J Natl Cancer Cent 2023, 3, 21. 39036314 10.1016/j.jncc.2023.01.002 PMC11256685

[5] H. Wang , H. Tang , Y. Fang , L. Tan , J. Yin , Y. Shen , Z. Zeng , J. Zhu , Y. Hou , M. Du , J. Jiao , H. Jiang , L. Gong , Z. Li , J. Liu , D. Xie , W. Li , C. Lian , Q. Zhao , C. Chen , B. Zheng , Y. Liao , K. Li , H. Li , H. Wu , L. Dai , K.‐N. Chen , JAMA Surg 2021, 156, 444. 33729467 10.1001/jamasurg.2021.0133 PMC7970392

[6] M. Abal , J. Andreu , I. Barasoain , Curr. Cancer Drug Targets 2003, 3, 193. 12769688 10.2174/1568009033481967

[7] X. Li , L. Chen , S. Luan , J. Zhou , X. Xiao , Y. Yang , C. Mao , P. Fang , L. Chen , X. Zeng , H. Gao , Y. Yuan , Semin. Cancer Biol. 2022, 86, 873. 35074509 10.1016/j.semcancer.2022.01.007

[8] L. G. M. Daenen , J. M. L. Roodhart , M. Van Amersfoort , M. Dehnad , W. Roessingh , L. H. Ulfman , P. W. B. Derksen , E. E. Voest , Cancer Res. 2011, 71, 6976. 21975929 10.1158/0008-5472.CAN-11-0627

[9] A. V. Thatishetty , N. Agresti , C. B. O'Brien , Clin. Liver Dis. 2013, 17, 671. 24099024 10.1016/j.cld.2013.07.010

[10] Y. Jun , Z. Tang , C. Luo , B. Jiang , X. Li , M. Tao , H. Gu , L. Liu , Z. Zhang , S. Sun , K. Han , X. Yu , X. Song , G. Tao , X. Chen , L. Zhang , Y. Gao , Q. Wang , ACS Appl. Mater. Interfaces 2020, 12, 47330. 32997489 10.1021/acsami.0c15419

[11] B. Li , H. Shao , L. Gao , H. Li , H. Sheng , L. Zhu , Drug Deliv 2022, 29, 2130. 35815678 10.1080/10717544.2022.2094498 PMC9275501

[12] C. Wang , D. Yang , L. Jiang , S. Wang , J. Wang , K. Zhou , X. Shi , L. Chang , Y. Liu , Y. Ke , H. Liu , Bioorg. Med. Chem. Lett. 2017, 27, 2058. 28285918 10.1016/j.bmcl.2017.02.008

[13] X. Deng , J. Sheng , H. Liu , N. Wang , C. Dai , Z. Wang , J. Zhang , J. Zhao , E. Dai , Biol. Pharm. Bull. 2019, 42, 1500. 31474710 10.1248/bpb.b19-00174

[14] X. Su , C. Gao , F. Shi , X. Feng , L. Liu , D. Qu , C. Wang , Drug Deliv 2017, 24, 10. 28155336 10.1080/10717544.2016.1225854 PMC8241110

[15] J. Ying , M. Zhang , X. Qiu , Y. Lu , Biomed. Pharmacother. 2018, 103, 381. 29674273 10.1016/j.biopha.2018.04.088

[16] J. Gu , P. Zhang , H. Li , Y. Wang , Y. Huang , L. Fan , X. Ma , X. Qian , J. Xi , ACS Nano 2024, 18, 6229. 38345570 10.1021/acsnano.3c09528

[17] S. Zong , X. Li , G. Zhang , J. Hu , H. Li , Z. Guo , X. Zhao , J. Chen , Y. Wang , Z. Jing , Phytomedicine 2024, 130, 155611. 38776737 10.1016/j.phymed.2024.155611

[18] Z.‐B. Jiang , W.‐J. Wang , C. Xu , Y.‐J. Xie , X.‐R. Wang , Y.‐Z. Zhang , J.‐M. Huang , M. Huang , C. Xie , P. Liu , X.‐X. Fan , Y.‐P. Ma , P.‐Y. Yan , L. Liu , X.‐J. Yao , Q.‐B. Wu , E. Lai‐Han Leung , Cancer Lett 2021, 515, 36. 34052328 10.1016/j.canlet.2021.05.019

[19] H.‐T. Wu , J. Lin , Y.‐E. Liu , H.‐F. Chen , K.‐W. Hsu , S.‐H. Lin , K.‐Y. Peng , K.‐J. Lin , C.‐C. Hsieh , D.‐R. Chen , Phytomedicine 2021, 81, 153437. 33352494 10.1016/j.phymed.2020.153437

[20] T. Qin , J. Zhao , X. Liu , L. Li , X. Zhang , X. Shi , Y. Ke , W. Liu , J. Huo , Y. Dong , Y. Shen , Y. Li , M. He , S. Han , L. Li , C. Pan , C. Wang , Phytother Res 2021, 35, 6228. 34494324 10.1002/ptr.7267

[21] S. A. Rajput , A. Shaukat , K. Wu , I. R. Rajput , D. M. Baloch , R. W. Akhtar , M. A. Raza , A. Najda , P. Rafał , A. Albrakati , A. F. El‐Kott , M. M. Abdel‐Daim , Antioxidants 2021, 10, 1268. 34439516 10.3390/antiox10081268 PMC8389199

[22] X. Zhu , Z. Yu , L. Feng , L. Deng , Z. Fang , Z. Liu , Y. Li , X. Wu , L. Qin , R. Guo , Y. Zheng , Carbohydr. Polym. 2021, 268, 118237. 34127219 10.1016/j.carbpol.2021.118237

[23] Y. Jiang , Y. Zhou , C. Y. Zhang , T. Fang , Int J Nanomedicine 2020, 15, 3319. 32494132 10.2147/IJN.S249144 PMC7227817

[24] H. Xu , M. Hu , M. Liu , S. An , K. Guan , M. Wang , L. Li , J. Zhang , J. Li , L. Huang , Biomaterials 2020, 235, 119769. 31986348 10.1016/j.biomaterials.2020.119769 PMC7093100

[25] X. Wang , Y. Song , L. Yu , X. Xue , M. Pang , Y. Li , X. Luo , Z. Hua , C. Lu , A. Lu , Y. Liu , ACS Appl. Mater. Interfaces 2023, 15, 34360. 37432741 10.1021/acsami.3c03233

[26] D. Fan , Y. Cao , M. Cao , Y. Wang , Y. Cao , T. Gong , Signal Transduct Target Ther 2023, 8, 293. 37544972 10.1038/s41392-023-01536-y PMC10404590

[27] Q. Zhou , J. Xiang , N. Qiu , Y. Wang , Y. Piao , S. Shao , J. Tang , Z. Zhou , Y. Shen , Chem. Rev. 2023, 123, 10920. 37713432 10.1021/acs.chemrev.3c00062

[28] H. Ding , P. Tan , S. Fu , X. Tian , H. Zhang , X. Ma , Z. Gu , K. Luo , J Control Release 2022, 348, 206. 35660634 10.1016/j.jconrel.2022.05.056

[29] S. Wilhelm , A. J. Tavares , Q. Dai , S. Ohta , J. Audet , H. F. Dvorak , W. C. W. Chan , Nat. Rev. Mater. 2016, 1, 16014.

[30] R. Sun , J. Xiang , Q. Zhou , Y. Piao , J. Tang , S. Shao , Z. Zhou , Y. H. Bae , Y. Shen , Adv Drug Deliv Rev 2022, 191, 114614. 36347432 10.1016/j.addr.2022.114614

[31] N. Wettschureck , B. Strilic , S. Offermanns , Physiol. Rev. 2019, 99, 1467. 31140373 10.1152/physrev.00037.2018

[32] R. Reuten , S. Zendehroud , M. Nicolau , L. Fleischhauer , A. Laitala , S. Kiderlen , D. Nikodemus , L. Wullkopf , S. R. Nielsen , S. McNeilly , C. Prein , M. Rafaeva , E. M. Schoof , B. Furtwängler , B. T. Porse , H. Kim , K. J. Won , S. Sudhop , K. W. Zornhagen , F. Suhr , E. Maniati , O. M. T. Pearce , M. Koch , L. B. Oddershede , T. Van Agtmael , C. D. Madsen , A. E. Mayorca‐Guiliani , W. Bloch , R. R. Netz , H. Clausen‐Schaumann , et al., Nat. Mater. 2021, 20, 892. 33495631 10.1038/s41563-020-00894-0

[33] D. Shangguan , Y. Li , Z. Tang , Z. C. Cao , H. W. Chen , P. Mallikaratchy , K. Sefah , C. J. Yang , W. Tan , Proc. Natl. Acad. Sci. USA 2006, 103, 11838. 16873550 10.1073/pnas.0602615103 PMC1567664

[34] Z. Geng , L. Wang , K. Liu , J. Liu , W. Tan , Angew Chem Int Ed 2021, 60, 15459. 10.1002/anie.202102631 33904236

[35] F. Xie , J. Qiu , C. Sun , L. Feng , Y. Jun , C. Luo , X. Guo , B. Zhang , Y. Zhou , Y. Wang , L. Zhang , Q. Wang , Adv. Sci. 2024, 11, 2309084. 10.1002/advs.202309084 PMC11267304 38704694

[36] J. Chen , J. He , T. Bing , Y. Feng , Y. Lyu , M. Lei , W. Tan , Anal. Chem. 2024, 96, 10601. 38889444 10.1021/acs.analchem.4c01186

[37] C. Qi , W. Li , Y. Luo , S. Ni , M. Ji , Z. Wang , T. Zhang , X. Bai , J. Tang , B. Yuan , K. Liu , Int. J. Biol. Macromol. 2024, 273, 133134. 38876234 10.1016/j.ijbiomac.2024.133134

[38] X. Li , L. Zhang , X. Guo , F. Xie , C. Shen , Y. Jun , C. Luo , L. Liu , X. Yu , Z. Zhang , Q. Wang , Y. Gao , K. Xu , J. Nanobiotechnol. 2021, 19, 388. 10.1186/s12951-021-01135-5 PMC8614048 34823537

[39] N. Xu , C. Lai , Q.‐M. He , Y. Cai , H. Yu , W. Zhong , S. Chen , F.‐C. Wu , H. Chen , Life Sci. 2023, 332, 122078. 37734435 10.1016/j.lfs.2023.122078

[40] T. D. S. Moreira , A. D. O. Silva , B. R. F. Vasconcelos , E. D. S. Santos , A. C. C. De Sousa , J. V. B. De Freitas , Y. S. De Oliveira , L. M. T. Vidal , F. D. O. S. Ribeiro , A. R. De Araújo , J. D. B. Vieira Neto , C. D. Ó. Pessoa , R. Petrilli , J. O. Eloy , Pharmaceutics 2023, 15, 915.36986777

[41] C. Isalomboto Nkanga , A. Murhimalika Bapolisi , N. Ikemefuna Okafor , R. Werner Maçedo Krause , Liposomes – Advances and Perspectives, IntechOpen, London, UK 2019.

[42] H. Ren , Y. He , J. Liang , Z. Cheng , M. Zhang , Y. Zhu , C. Hong , J. Qin , X. Xu , J. Wang , ACS Appl. Mater. Interfaces 2019, 11, 20304. 31056910 10.1021/acsami.8b22693

[43] M. Jain , J. R. Seth , L. R. Hegde , K. P. Sharma , Macromolecules 2020, 53, 8974.

[44] H. Shah , A. Madni , M. M. Khan , F.‐D. Ahmad , N. Jan , S. Khan , M. A. Rahim , S. Khan , M. M. Ali , M. Kazi , Pharmaceutics 2022, 14, 129. 35057025 10.3390/pharmaceutics14010129 PMC8779429

[45] C. Sun , W. Li , P. Ma , Y. Li , Y. Zhu , H. Zhang , M. Adu‐Frimpong , W. Deng , J. Yu , X. Xu , Food Chem. Toxicol. 2020, 137, 111126. 31954714 10.1016/j.fct.2020.111126

