# Supplementary material for: Paclitaxel/Luteolin Coloaded Dual‐Functional Liposomes for Esophageal Cancer Therapy
Source: Adv Sci (Weinh). 2025 Apr 23;12(20):2411930. doi: 10.1002/advs.202411930 (PMC12120766; doi:10.1002/advs.202411930)
Supplement: Supplementary file 1 — Supporting Information [file ADVS-12-2411930-s001.docx]

Supporting Information

**Paclitaxel/Luteolin Coloaded Dual-functional Liposomes for Esophageal Cancer Therapy**

*Congyong Su**n*^*,^*^#^, Fei Xie**^#^, Huiyun Zhang^#^,* *Lulu Feng^#^, Yuting Wang, Chaofan Huang, Zhizhen Cui,* *Chao Luo**, Li Zhang*^*^*, and Qilong Wang*^*^

C. Sun, F. Xie, L. Feng, Y. Wang, C. Huang, C. Luo, L. Zhang, and Q. Wang

The Comprehensive Cancer Centre, Department of Central Laboratory, The Affiliated Huai’an No.1 People’s Hospital, Nanjing Medical University, Huai’an, Jiangsu, 223300, China

E-mail: [qlwang@njmu.edu.cn](mailto:qlwang@njmu.edu.cn), [zhangl@njmu.edu.cn](mailto:zhangl@njmu.edu.cn), [cysun@njmu.edu.cn](mailto:cysun@njmu.edu.cn)

H. Zhang

Department of Pharmaceutical Engineering, School of Chemistry and Chemical Engineering, Yancheng Institute of Technology, Yancheng, Jiangsu, 224003, China

Z. Cui

Department of Acute Infectious Disease Control and Prevention, Huai’an Center for Disease Control and Prevention, Huai’an, Jiangsu, 223003, China

Congyong Sun, Fei Xie, Huiyun Zhang and Lulu Feng contributed equally to this work.

Table of contents

[1 Materials and methods 4](#_Toc193812165)

[1.1 Materials 4](#_Toc193812166)

[1.2 Cell lines 5](#_Toc193812167)

[1.3 Tumor tissue sections 6](#_Toc193812168)

[1.4 *In vitro* synergistic anti-ESCC effects of PTX and LUT 6](#_Toc193812169)

[1.5 Real-time cell analysis (RTCA) 7](#_Toc193812170)

[1.6 Colony formation assay 7](#_Toc193812171)

[1.7 Cell migration and invasion 7](#_Toc193812172)

[1.8 *In vivo* synergistic anti-ESCC effects 8](#_Toc193812173)

[1.9 HE, Ki67, and TUNEL assays 8](#_Toc193812174)

[1.10 *In vitro* hepatoprotective effect of LUT against PTX-induced hepatotoxicity 9](#_Toc193812175)

[1.11 EdU staining 9](#_Toc193812176)

[1.12 Measurement of ALT, AST, MDA, and SOD levels 9](#_Toc193812177)

[1.13 Western blotting analysis of hepatoprotective mechanism 10](#_Toc193812178)

[1.14 *In vivo* hepatoprotective effect of LUT against PTX-induced hepatotoxicity 10](#_Toc193812179)

[1.15 Cell-SELEX procedure 11](#_Toc193812180)

[1.16 Binding analysis of EA2 12](#_Toc193812181)

[1.17 ssDNA pull-down assay of EA2 14](#_Toc193812182)

[1.18 Liquid chromatography–tandem mass spectrometry (LC-MS/MS) analysis 14](#_Toc193812183)

[1.19 Binding ability of EA2 to CTNNA1 15](#_Toc193812184)

[1.20 Molecular docking 16](#_Toc193812185)

[1.21 Microscale thermophoresis assays (MST) 16](#_Toc193812186)

[1.22 Competitive binding between EA2 and CTNNA1 antibody 17](#_Toc193812187)

[1.23 *In vivo* targeting ability of EA2 17](#_Toc193812188)

[1.24 Development of EA2 aptamer-modified and PTX/LUT-co-loaded PSL 18](#_Toc193812189)

[1.25 Characterization of EA2-PSL-PTX/LUT 19](#_Toc193812190)

[1.26 Entrapment eﬃciency (EE) and drug loading (DL) 19](#_Toc193812191)

[1.27 Dynamic light scattering (DLS) and zeta potential 20](#_Toc193812192)

[1.28 Transmission-electron microscopy (TEM) 20](#_Toc193812193)

[1.29 Stability 20](#_Toc193812194)

[1.30 Hemolysis test 21](#_Toc193812195)

[1.31 Biological safety evaluation 21](#_Toc193812196)

[1.32 Cellular uptake 22](#_Toc193812197)

[1.33 Internalization mechanism study 22](#_Toc193812198)

[1.34 Endosomal escape study 23](#_Toc193812199)

[1.35 Targeting of EA2-modified PSLs 23](#_Toc193812200)

[1.36 Biodistribution of nanoparticles in blood cells 24](#_Toc193812201)

[1.37 *In vivo* biodistribution 24](#_Toc193812202)

[1.38 *In vivo* tumor penetration 24](#_Toc193812203)

[1.39 Biodistribution of nanoparticles in tumors via flow cytometry assay 25](#_Toc193812204)

[1.40 Immune cell recruitment ability 25](#_Toc193812205)

[1.41 *In vitro* therapeutic efficacy 25](#_Toc193812206)

[1.42 *In vivo* anti-tumor efficacy 26](#_Toc193812207)

[1.43 *In vivo* remodeling effects of EA2-PSL-PTX/LUT on TME 26](#_Toc193812208)

[2 Supporting Figures 28](#_Toc193812209)

[3 Supporting Tables 46](#_Toc193812210)

[4 References 50](#_Toc193812211)

**1 Materials and methods**

**1.1 Materials**

PTX (HY-B0015), LUT (HY-N0162), dexamethasone (HY-14648), DiD dye (HY-D1028), genistein (HY-14596), chlorpromazine hydrochloride (HY-B0407A), colchicine (HY-16569), sodium orthovanadate (HY-D0852), wortmannin (HY-10197), cytochalasin D (HY-N6682), chloroquine phosphate (HY-17589), and nystatin (HY-17409) were purchased from MedChemExpress. Absolute ethanol, methanol, and dichloromethane were purchased from Sinopharm Chem. Reagent Co., Ltd. (Shanghai, China). DOPE, CHEMS, DiR dye, and crystal violet were obtained from Shanghai Macklin Biochemical Technology Co., Ltd. Dimethyl sulfoxide (DMSO), proteinase K, branched polyethylenimine (PEI, Mw~25000), and 4,6-diamidino-2-phenylindole (DAPI) were purchased from Sigma-Aldrich. TPGS, coumarin-6 (C6), acetic acid, and Tween 80 were purchased from Aladdin Industries Corp. (Shanghai, China). Chromatographically pure methanol was obtained from TEDIA Company, Inc. (Fairfield, USA). Invitrogen (Carlsbad, CA-USA) supplied the reagents fetal bovine serum (FBS), nonessential amino acid (NEAA), LysoTracker Red, ITS media supplement, DMEM basic, DMEM/F12 (DF12), MEM, RMPI 1640 media, and Alexa Fluor™ 594 goat anti-mouse IgG (H+L) cross-adsorbed secondary antibody. Trypsin, CCK-8, and penicillin–streptomycin were obtained from Beyotime Institute of Biotechnology (Jiangsu, China). Matrigel and Transwell plates were purchased from Corning. The anti-CTNNA1, anti-Ki67, Nrf2, Keap1, HO-1 and NQO-1 antibodies were procured from Abcam, and HRP-conjugated goat anti-mouse IgG was from Proteintech. The Cell-Light EdU Apollo567 *In Vitro* Kit (C10310-1) and RiboAPO One-Step TUNEL Apoptosis Kit (C11026-1) were purchased from Guangzhou RiboBio Co., Ltd. d-Luciferin potassium salt was obtained from PerkinElmer. The ssDNA library, EA2 aptamer, FAM- or Cy5-labeled EA2, and siCTNNA1 were synthesized by GenePharma. Nanjing JianCheng Bioeng. Inst. (Nanjing, China) provided the commercial reagent kits for determining alanine transaminase (ALT), aspartate transaminase (AST), lactate dehydrogenase (LDH), alkaline phosphatase (AKP), superoxide dismutase (SOD), malondialdehyde (MDA), total bile acid (TBA), uric acid (UA), creatinine (CRE), urea (BUN) and BCA protein. Double-distilled water (ddH_2_O) was prepared and purified using a Millipore water system (Millipore Corp., Bedford, MA, USA). All chemicals were commercially obtained and analytically pure.

**1.2** **Cell lines**

Human ESCC KYSE-150 and KYSE-30 cells were procured from the BeNa Culture Collection (BNCC) and authenticated by the Henan Engineering Research Center of Industrial Microbiology. Luciferase-expressing KYSE-150 cells (Lucif-KYSE-150 cells) were engineered in our laboratory. Other cancer cell lines, including human esophageal cancer (Eca9706 and KYSE-410), human breast cancer (MCF-7), human gastric adenocarcinoma (AGS), human acute lymphoblastic leukemia (Nalm-6), human colorectal cancer (SW620), human cervical cancer (HeLa), human epidermoid carcinoma (A431) human lung adenocarcinoma (A549), human malignant glioma (U251), human hepatocellular carcinoma (HepG2), human cervical squamous cell (SiHa), human colon cancer cell (HCT-116), human tongue squamous cell carcinoma (CAL-27), and human pharyngeal squamous cell (FaDu) were all sourced from the Cell Bank of the Chinese Academy of Sciences (Shanghai, China). The human esophageal epithelial cells (HEEC) and normal mouse hepatocytes AML12 (alpha mouse liver 12) were obtained from Procell Life Science & Technology Co., Ltd. All cells were cultured at 37 °C under 5% CO_2_ in a humidified atmosphere. AML12 cells were nurtured in DF12 supplemented with 10% FBS, 1% ITS media supplement, dexamethasone (40 ng/mL), streptomycin (100 μg/mL), and penicillin (100 U/mL). The culture medium for KYSE-150, Eca9706, KYSE-30, KYSE-410, A549, A431, U251, HCT-116, CAL-27, and FaDu comprised DMEM with 10% FBS and antibiotics. AGS and Nalm-6 cells were maintained in RPMI1640, whereas HeLa, SiHa, HepG2, and HEEC were cultured in MEM with NEAA. Conversely, SW620 and MCF-7 were maintained in Leibovitz's L-15, and MEM with NEAA and 10 μg/mL insulin, respectively.

**1.3 Tumor tissue sections**

All paraffin-embedded ESCC slides (including tumor tissues and adjacent normal esophageal tissues) required for tissue imaging were provided by the Department of Thoracic Surgery, Affiliated Huai’an No. 1 People’s Hospital of Nanjing Medical University. The associated research on tissue specimens was approved by the Ethics Committee of the Affiliated Huai’an No. 1 People’s Hospital of Nanjing Medical University (KY-2024-143-01). Paraffin-embedded slides of breast, cervical, colon, stomach, liver, and ovarian cancers (two cases for each disease) were procured from Shanghai YEPCOME Biotechnology Co., Ltd. The tissue pretreatment procedure was conducted as follows: Slides were subjected to heating in an oven at 65 °C for 1 h, followed by immediate deparaffinization in xylene (15 and 12 min, once each) and subsequent rehydration through a series of graded ethanol solutions (100%, 95%, 85%, and 70% ethanol, 5 min each). After washing twice with PBS buffer, the rehydrated tissue slides were immersed in sodium citrate buﬀer (0.01 M, pH 6.0) and then exposed to a microwave at 98 °C for 15 min to facilitate antigen retrieval for further analysis.

**1.4 *In vitro* synergistic anti-ESCC effects of PTX and LUT**

KYSE-150 cells (2×10^3^ cells/well, 100 μL) at the exponential growth phase were seeded into 96-well plates and incubated overnight. The cells were treated with varying concentrations of PTX (1, 2, 5, 10, 20, 40, 60, and 80 nM), and LUT (10, 20, 40, 60, 80, 100, 120, 160, and 200 μM). After 24, 48, and 72 h incubation, the cells were incubated with CCK-8 solution (10 μL each well) for 2 h at 37 °C. The OD_450nm_ value was determined using a microplate reader (Epoch, BioTek, USA). The relative cell viability was calculated by the formula: Cell viability (%) = (OD_sample_ – OD_blank_)/(OD_untreated_ – OD_blank_) × 100%. The IC_50_ values of PTX and LUT were calculated using the GraphPad software. For synergistic investigation, cells were treated with combinations of PTX (0, 6, and 12 nM) and LUT (0, 11.25, 22.5, 45, and 90 μM) for 48 h. Cell morphology was captured using a phase-contrast microscope (Leica, USA), LDH leakage in the medium was measured, and cell viabilities were assessed using the CCK-8 assay. Drug interactions were analyzed using the median effect principle ^[1]^. The combination index (CI) was calculated using CompuSyn software, where CI = 1 denotes an additive effect, CI < 1 indicates a synergistic effect, and CI > 1 suggests an antagonistic effect.

**1.5 Real-time cell analysis (RTCA)**

KYSE-150 cells were seeded in E-plates (2×10^3^/well, 100 μL), and then treated with PBS, PTX (12 nM), LUT (22.5 μM), or a combination of PTX and LUT for 72 h incubation. Cell growth curves were automatically monitored and recorded using the xCELLigence System RTCA (Agilent, USA).

**1.6 Colony formation assay**

KYSE-150 cells were seeded into 6-well plates (2×10^3^ cells/well, 2000 μL), and then treated with PBS, PTX (12 nM), LUT (22.5 μM), or a combination of PTX and LUT. Following 48 h of incubation, the medium was replaced with fresh medium, and the cells were cultured for an additional 12 days. Cell colonies were fixed with 4% paraformaldehyde (PFA) and stained with 0.1% crystal violet dye. Finally, cell colonies (>50 cells) were captured and counted using ImageJ software.

**1.7 Cell migration and invasion**

For the wound-healing assay, KYSE-150 cells (6×10^5^/well, 2000 μL) were seeded in 6-well plates and incubated at 37 °C. Following 24 h of incubation, once the cell monolayer reached confluence, it was mechanically scratched using a 20 μL pipette and washed gently with PBS three times. Subsequently, cells were treated with PBS, PTX (12 nM), LUT (22.5 μM), or a combination of PTX and LUT in DMEM medium (with 0.5% FBS) for an additional 48 h. Images were captured at 0, 12, 24, and 48 h by phase-contrast microscope. The wound areas were quantified using ImageJ with Wound_healing_size_tool. For invasion assays, KYSE-150 cells (2×10^4^/well, 200 μL) were suspended in DMEM basic medium, and seeded into the upper Transwell chambers (8 mm pore size, NEST, Matrigel-coated) in a 24-well plate. Subsequently, cells were treated with PBS, PTX (12 nM), LUT (22.5 μM), or a combination of PTX and LUT in DMEM medium. The lower chambers were filled with DMEM supplemented with 10% FBS. After 48 h of incubation, the cells on the upper surface of the membrane were removed by gentle scraping with a cotton swab. Simultaneously, cells on the bottom surface of the membrane were fixed with 4% PFA and stained with 0.1% crystal violet. The penetrated cells were captured in five random fields. Cells were destained using DMSO for detecting OD value at 570 nm.

**1.8 *In vivo* synergistic anti-ESCC effects**

For the xenograft models, KYSE-150 cells were subcutaneously (5×10^6^ cells resuspended in 100 μL PBS) injected into BALB/c nude mice. Once the tumor volume reached approximately 100 mm^3^, mice were randomized to the following treatment groups: PBS (0.1 mL), PTX (5 mg/kg), LUT (50 mg/kg), and PTX plus LUT. The mice were intravenously treated for seven times. Tumor length and width and animal body weight were monitored every 3 days. Tumor volumes were calculated using the following formula: tumor volume (mm^3^) = length × width × width/2. On the 21st day, blood samples were collected for evaluating the liver function by measuring serum ALT, AST, AKP, and LDH levels. The mice were then sacrificed, and the tumors were excised and weighed. Tumors were fixed in 4% PFA and embedded in paraffin for histological analysis.

**1.9 HE, Ki67, and TUNEL assays**

Hematoxylin and eosin (HE) staining was used to distinguish between normal and pathological tissues. Specifically, 5 μm tissue sections were obtained from tumor samples. They were deparaffinized, rehydrated, and rinsed as previously described. Sections were stained with HE, dehydrated in a graded alcohol series, cleared in xylene, and cemented with a neutral resin. Ki-67, a marker of cell proliferation indicative of the cell cycle phase distribution, was used for immunostaining. Tissue sections were incubated with anti-Ki67 antibody, followed by incubation with goat anti-rabbit peroxidase-conjugated secondary antibody. They were finally stained with 3,3-diaminodbenzidine substrate and hematoxylin. TUNEL staining was conducted using a TUNEL staining kit to label the DNA strand breaks. The prepared sections were incubated with proteinase K for 15 min at 37 °C, and then incubated with the TUNEL reaction solution. Apoptosis was visualized by red fluorescence and the cell nuclei were counterstained with DAPI. The histological images were captured using an upright fluorescence microscope (Leica, USA).

**1.10 *In vitro* hepatoprotective effect of LUT against PTX-induced hepatotoxicity**

AML12 cells were chosen to establish *in vitro* PTX-induced hepatotoxicity. AML12 (5×10^3^ cells/well, 100 μL) at exponential growth phase were seeded into 96-well plates and incubated overnight, then cells were treated with varying concentrations of PTX (10, 20, 50, 100, 200, 300, 400, and 500 nM) in FBS-free DMEM. After 24 h of incubation, CCK-8 assays were performed, and the IC_50_ was calculated to determine the concentration of PTX resulting in cell injury. To investigate the hepatoprotective effect of LUT against PTX-induced hepatotoxicity, AML12 (5×10^3^ cells/well, 100 μL) were seeded into 96-well plates and incubated for 24 h. They were then pretreated with various concentrations of LUT (0, 10, 20, and 40 μM) for 24 h before being challenged with PTX (150 nM) for another 24 h. Cell viability was assessed using the CCK-8 assay. Cell morphology was observed using an inverted microscope (Leica, USA), and released LDH by AML12 hepatocytes were detected using a colorimetric assay kit, according to the protocols provided by the manufacturer.

**1.11 EdU staining**

EdU is a thymine nucleoside analog involved in replicating DNA molecules during cell proliferation. The EdU assay, in which only newly synthesized DNA are labeled during cell division, is used to validate the antiproliferative activity in PTX-induced hepatotoxicity. For EdU staining, after treatment with LUT and PTX, cells were incubated with 50 μM EdU for 4 h, and fixed with 4% PFA for 30 min. After washing with PBS, the cells were stained with APOLLO dye. Proliferating cells were visualized by red fluorescence, and cell nuclei were counterstained with DAPI.

**1.12 Measurement of ALT, AST, MDA, and SOD levels**

AML12 cells (6×10^5^/well, 2000 μL) were seeded in 6-well plates and incubated at 37 °C for 24 h. Then, cells were pretreated with various concentrations of LUT (0, 10, 20, and 40 μM) for 24 h before being challenged with PTX (150 nM) for another 24 h. Subsequently, AML12 cells and the supernatant of the cell culture were collected. ALT, AST, and AKP levels in the cell supernatants and MDA and SOD levels in the cells were determined using commercial kits. All procedures were performed according to the manufacturer’s instructions.

**1.13** **Western blotting analysis of hepatoprotective mechanism**

Following the above-described treatment, AML12 cells were harvested and subjected to protein extraction. Cells were resuspended in ice-cold RIPA lysis buffer supplemented with protease inhibitors (1 mM PMSF and 1× protease inhibitor cocktail), followed by vigorous vortexing for 15 s to ensure uniform lysis. The cell suspension was incubated on ice for 20 min to facilitate complete protein solubilization, after which lysates were centrifuged at 12000 rpm for 10 min at 4°C. The resulting supernatants were carefully transferred to fresh tubes and stored at −80°C until further use.

Equal amounts of protein (25 μg per sample) were separated by electrophoresis in 10% or 12.5% SDS-PAGE and transferred to PVDF membranes (Millipore, USA). After blocking with 5% BSA at room temperature for 1 h, proteins were, respectively incubated with primary antibodies (Nrf2, Keap1, HO-1 and NQO1) at 4 ℃ overnight. After several washes with TBST, membranes were incubated with the HRP-conjugated secondary antibodies for 2 h at room temperature. Finally, protein expression was detected using an enhanced chemiluminescence reagent (ZETA LIFE, USA) and quantified using ImageJ software. Protein levels were standardized by comparison with β-actin.

**1.14 *In vivo* hepatoprotective effect of LUT against PTX-induced hepatotoxicity**

PTX was resuspended in 5% DMSO, 40% polyethylene glycol 3000, and 5% Tween 80 in ddH_2_O. C57BL/6J mice were injected intraperitoneally with PTX (10 mg/kg) for five consecutive days to induce hepatotoxicity ^[2]^. The PBS group were injected intraperitoneally with PBS. Pretreated C57BL/6J mice were randomized into five groups (n = 5): PBS, PTX, LUT (25 mg/kg) + PTX, and LUT (50 mg/kg) + PTX. LUT was administered intravenously at doses of 25 and 50 mg/kg once daily for seven consecutive days. The mice in the control and model groups were administered PBS. The animals were sacrificed 1 h after the last LUT administration. Retro-orbital bleeding was applied to collect blood samples. Serum was obtained via centrifugation for 10 min at 3700 rpm, and liver tissues were collected and stored for the test. Serum ALT and AST activities were selected as indicators of hepatic functions and assayed via standard diagnostic kits in accordance with the speciﬁcations of the manufacturer. The levels of MDA and SOD in the liver tissues were determined according to the manufacturer’s instructions. The liver tissues were weighed and fixed in 4% formalin; they were embedded in paraffin and sectioned into 5 μm tissue sections. The slices were stained with HE and photographed under a light microscope.

**1.15 Cell-SELEX procedure**

The GenePharma (Shanghai, China) synthesized ssDNA library and primers used in our previous study ^[3]^ were utilized in this study as well. The 79-nt ssDNA library comprised a 32-nt randomized sequence and two primers ﬂanked on each side for polymerase chain reaction (PCR) ampliﬁcation (5’-AGCCTAAGCCTGTCCAGGAATCG-N32-ATGGCTTAGTGGCACGATTAGGTC-3’). The forward primer was fluorescently labeled with FAM at the 5’ end (5’‑FAM‑AGCCTAAGCCTGTCCAGGAATCG‑3’) to monitor the progress of the selection via ﬂow cytometry. The reverse primer was biotinylated at the 5’ end (5’‑Biotin‑GACCTAATCGTGCCACTAAGCCAT‑3’) for separation using streptavidin-coated sepharose beads. The washing buffer comprised 0.01 M DPBS combined with 5 mM MgCl_2_ and 4.5 g/L glucose. The binding buffer was formulated by adding 0.1 mg/mL yeast tRNA and 1 mg/L bovine serum albumin (BSA) into the washing buffer.

The cell-SELEX procedure was executed as depicted in **Figure S5a**. For positive selection, 1.5×10^6^ KYSE-150 cells were incubated with the DNA library on a rotary shaker at 4 °C for 60 min. After incubation, the cells were rinsed thrice with washing buffer to remove unbound sequences. Subsequently, the harvested cells were heated at 95 °C for 10 min to elute the cell-bound ssDNA from the cell surface. This was followed by centrifugation at 15,000 rpm to remove cell debris. The eluted ssDNA was then subjected to PCR amplification (3 min at 95 °C, 35 cycles of 15 s at 95 °C, 20 s at 68 °C, and 90 s at 72 °C, followed by 10 min at 72 °C) to enrich the selected pool. Next, the PCR products were incubated with streptavidin-modified sepharose beads for 30 min at room temperature. Denaturation was performed using 200 mM NaOH for 10 min, followed by centrifugation for separation. The resulting ssDNA library was desalted, quantified, and dried for the next round of selection. Negative selection was implemented in the 4th round of selection to remove nonspecific bound sequences. Specifically, the ssDNA library was incubated with HEEC at 4 °C for 30 min; the unbound DNA in the supernatant was collected for the subsequent round of positive selection. In the ensuing selection process, screening pressures were progressively intensified to enhance specificity and affinity. The incubation time for negative selection was extended from 30 to 60 min and the number of washing cycles was increased from three to five. Meanwhile, the incubation time of positive selection was reduced from 60 to 30 min; the dosage of the aptamer pool used for selection was also reduced from 1.4 nmol to 30 pmol. After 15 rounds of selection, ssDNA was amplified and subcloned into the pUC19 vector in *Escherichia coli* DH5a cells. Selected clones (EA2) were sequenced (**Table S1**), and their secondary structures were analyzed using DNAMAN v.3.2.

**1.16 Binding analysis of EA2**

To assess the binding ability of EA2, 1×10^5^ target KYSE-150 cells and control HEEC were incubated with 250 nM FAM-labeled EA2 in 200 μL binding buﬀer at 4 °C for 45 min. To evaluate the binding specificity of the EA2, control aptamers (including Sgc8, EGFR, and AS1411 at 250 nM, as summarized in **Table S1**) labeled with FAM were synthesized by Sangon Biological Technology Co., Ltd. (Shanghai, China) and incubated with 1×10^5^ KYSE-150 cells. Additionally, ESCC cells, such as KYSE-30, Eca9706, and KYSE-410, along with other cancer cell lines including MCF-7, AGS, Nalm-6, SW620, HeLa, A431, A549, U251, and HepG2, were incubated with 250 nM FAM-labeled EA2 at 4 °C for 60 min. After incubation, the cells were washed three times and resuspended in 400 μL binding buffer. The cells were analyzed by flow cytometry using FITC channel (BD, C6 plus, USA).

To determine the equilibrium dissociation constants (*K*_d_) of EA2, increasing concentrations (0, 50, 100, 150, 200, 250, and 300 nM) of FAM-labeled EA2 were incubated with 1×10^5^ KYSE-150 cells at 4 °C for 60 min, and the fluorescence signal was analyzed by flow cytometry. The *K*_d_ value was calculated using GraphPad Prism 8 software according to the one-site saturation equation: *Y* = *B*_max_ *X*/(*K*_d_ + *X*). To preliminarily identify the target type of EA2, KYSE-150 and KYSE-30 cells were treated with 0.1 mg/mL proteinase K for 10 min to degrade cell-surface proteins. Then, treated cells were incubated with FAM-labeled EA2 at 4 °C for 60 min. After washing, fluorescence signals were detected by flow cytometry. FAM-labeled random ssDNA served as the negative control. All experiments were repeated three times.

To confirm the binding ability of EA2, 4×10^4^ KYSE-150 cells and HEEC were seeded into confocal dishes (NEST) overnight. After the cells were washed with PBS three times, they were incubated with 250 nM FAM-labeled EA2 in binding buffer at 4 °C for 1 h, and 37 °C for 1 h. The cells were then washed, fixed with 4% PFA for 20 min, and then stained with DAPI (10 μg/mL) for 10 min to counterstain the nucleus. Fluorescence images were captured using laser scanning confocal microscopy (LSCM) (Nikon, Japan). To further ascertain the feasibility of using EA2 in clinical tissue samples, various tumor tissue slides, including ESCC and breast, cervical, colon, gastric, liver, and ovarian cancers, and paired adjacent tissues, were subjected to deparaffinization, rehydration, and antigen retrieval, as previously described. Tissue slides were washed with washing buffer three times and incubated for 30 min with 250 nM random sequences at 4 °C for blocking. After washing, the slides were incubated with 250 nM Cy5-labeled EA2 in binding buffer for 1 h. Post incubation, the slides were washed, stained with DAPI for 10 min, and further washed three times for observation via LSCM. All fluorescence images were captured using LSCM. The excitation and emission ranges for FAM, DAPI, and Cy5 were 488/512, 410/503, and 649/670 nm, respectively.

**1.17 ssDNA pull-down assay of EA2**

The target molecule of EA2 was identified by performing an ssDNA pull-down assay. Specifically, 2×10^7^ KYSE-150 cells were meticulously washed thrice with PBS at 4 °C and harvested using a cell scraper. The collected cells were lysed with RIPA buﬀer (containing 0.1 mM PMSF and 1× cocktail protease inhibitors) at 4 °C for 30 min. Following centrifugation at 4 °C for 30 min, the supernatant containing total proteins was isolated for the enrichment of integral membrane proteins utilizing the Mem-PER™ Plus kit (Thermo Fisher, CA, USA). Subsequently, 300 μg membrane proteins were incubated with 250 nM biotin-labeled random sequences or biotin-labeled EA2 at 4 °C for 45 min. Then, random sequence–protein and aptamer–protein complexes were captured by incubating them with 50 μL streptavidin-modified magnetic beads at 4 °C for 1 h. The retrieved magnetic beads were washed four times with cold PBS. The captured proteins were eluted using 40 μL of protein loading buffer, and then heated at 95 °C for 5 min to denature the proteins. The captured proteins were subjected to 10% sodium dodecyl sulfate-polyacrylamide gel electrophoresis (SDS-PAGE) and silver staining (Beyotime Biotech P0017S), followed by scanning using a Bioimage system (UVP GelDoc-It2 Imager).

**1.18 Liquid chromatography–tandem mass spectrometry (LC-MS/MS) analysis**

Specific aptamer-purified protein bands were excised for digestion and subsequently subjected to LC-MS/MS analysis conducted by Zoonbio Biotechnology (Nanjing, China). LC-MS/MS-based quantitative proteomics using Q Exactive Plus (Thermo Fisher Scientific) was employed to screen, identify, and quantify distinctive proteins. Data analysis was performed using the Proteome Discoverer platform (Version 2.1, Thermo), with MASCOT 2.6 serving as the database search engine.

**1.19 Binding ability of EA2 to CTNNA1**

4×10^4^ KYSE-150 cells were seeded into confocal dishes overnight. After the cells were washed with PBS three times, they were fixed with 4% PFA for 20 min, washed thrice with PBS, and then treated with 5% BSA for 30 min at room temperature. Cells were washed twice with PBS and incubated overnight at 4 °C with primary CTNNA1 antibody. Subsequently, 100 nM FAM-labeled EA2 and Alexa Fluor™ 594 goat anti-mouse IgG (H+L) cross-adsorbed secondary antibody were incubated with cells at 4 °C for 2 h. Nuclei were counterstained with DAPI. Fluorescence images were captured using LSCM.

To validate CTNNA1 as a target protein, transient small interfering RNAs (siRNA) transfection of CTNNA1 into KYSE-150 cells was performed. Three siRNA sequences targeting CTNNA1 and a negative control were designed and procured from GenePharma (**Table S2**). KYSE-150 cells were seeded into 6-well culture plates (6×10^5^ cells/well). Following a 24-h incubation period, cells were transfected with siRNA for 6 h using Lipofectamine 3000 (Thermo). Subsequently, after 48 h of incubation, total protein was extracted for Western blotting using an anti-CTNNA1 antibody. siCTNNA1 transfected KYSE-150 cells, untreated KYSE-150 cells, HEEC, KYSE-30, KYSE-410, and Eca9706 were lysed in RIPA lysis buffer (containing 1mM PMSF and 1mM cocktail) on ice for 30 min. The lysate was then centrifuged at 4 ℃ for 20 min at 12,000 *g* to obtain the supernatant (total cellular protein). A BCA protein assay (Beyotime, Shanghai, China) was performed to measure protein concentration. Protein samples were denatured with 5×loading buffer at 100 ℃ and separated by SDS-PAGE. After transferring the proteins onto PVDF membranes (Millipore, USA), the membranes were blocked with 5% BSA at room temperature for 1 h. Next, the PVDF membranes were incubated with primary antibody (anti-CTNNA1 mouse, 1:1000, Abcam, USA) at 4 ℃ overnight. After several washes with TBST, the HRP-conjugated secondary antibody was incubated at room temperature for 2 h. Finally, CTNNA1 expression was detected using an enhanced chemiluminescence reagent (ZETA LIFE, USA) and quantified using ImageJ software. CTNNA1 expressions in CAL-27, FaDu, A549, HCT-116, U251, and SiHa cell lines were validated using the same procedure.

To further confirm the binding ability of EA2 aptamer to CTNNA1, 1×10^5^ target KYSE-150 cells or siCTNNA1 transfected KYSE-150 cells were incubated with 250 nM FAM-labeled EA2 in 200 μL binding buﬀer at 4 °C for 45 min. After the cells were washed and analyzed by flow cytometry.

**1.20 Molecular docking**

The molecular docking analysis was employed to identify potential binding sites between CTNNA1 and EA2. Initially, the 3D structure of CTNNA1 was retrieved from the AlphaFold database (https://alphafold.ebi.ac.uk/entry/P35221). Energy minimization procedures were carried out to eliminate water molecules, ionic groups, and heteroatoms, protonate the proteins, and flip the amino acid atoms using the MOE software, resulting in a rational conformation of CTNNA1. For the EA2 aptamer, the secondary structure was predicted using the RNAfold web server, followed by the construction of the 3D structure using RNAcomposer software. Subsequently, the nucleic acid bases U were converted to T, and energy minimization was performed to obtain a reliable structure for EA2 using the PyMol program. The ribonucleotides were then transformed into deoxynucleotides in the MOE software. The minimized structure of EA2 was docked to CTNNA1 using the HDOCK software, and a series of energy minimizations on the aptamer-protein complexes were conducted. The resulting minimized complex structure was utilized for further analysis. A conformational search was conducted to determine the top 10 optimal conformations for docking the receptors on frequently activated sites using PyMol. The results were determined by analyzing the systematic scores and interaction forces.

**1.21 Microscale thermophoresis assays (MST)**

The interaction between CTNNA1 and EA2 was quantified using MST experiments conducted on a Monolith NT.115 system (NanoTemper, Germany). CTNNA1 was labeled with the manufacturer’s Monolith Protein Labeling Kit RED-NHS 2nd Generation (Cat. No. Mo-L011, NanoTemper, Germany) Solutions of CTNNA1 (50 nM) were prepared in 10 mM PBS (pH 7.4), while EA2 solutions (ranging from 50 µM to 0.00153 µM) were prepared in 10 mM PBST (0.05% v/v Tween-20, pH 7.4). The mixture of CTNNA1 and EA2 were introduced into Monolith NT.115 capillaries (Cat.No.Mo-K022, Lot.NO.23K022.005, NanoTemper, Germany) and subsequently subjected to MST analysis. Kinetic constant (K_d_) was determined by fitting the curve using a single-site model.

**1.22 Competitive binding between EA2 and CTNNA1 antibody**

To further investigate EA2 binding site, competitive binding assay between EA2 and CTNNA1 antibody were performed using confocal imaging, flow cytometry and MST analysis. For confocal imaging, 4×10^4^ KYSE-150 cells were seeded into confocal dishes overnight. After washed with DPBS, cells were incubated with CTNNA1 antibody (1:200) at 4 °C for 4 h, followed by incubation with FITC-Goat Anti-Rabbit IgG (1:200) at 4 °C for 2 h, and finally incubated in 250 nM Cy5-labelled EA2 in binding buffer at 4 °C for 1 h. After washed with washing buffer twice, co-location of FITC and Cy5 fluorescence signal was imaged by LSCM. For flow cytometry analysis, 4×10^4^ KYSE-150 cells were incubated with CTNNA1 antibody (1:200) at 4 °C for 4 h, followed by incubation with 250 nM Cy-5 labeled EA2 in binding buffer at 4 °C for 1 h. Non-incubation with the CTNNA1 antibody served as the control group. After washing with washing buffer for 2 times, Cy5 fluorescence signal was detected by flow cytometry. For MST analysis, the effects of CTNNA1 antibody on the CTNNA1-EA2 interaction was quantified by measuring changes in binding affinity. Dissociation constants (*K_d_*) were determined in the presence of CTNNA1 antibody (0.2 and 1 µM).

**1.23 *In vivo* targeting ability of EA2**

Once the tumor volume reached approximately 100 mm^3^ in KYSE-150 tumor-bearing mice, Cy5-labeled EA2 (5 nmol/100 μL) was injected intravenously to mice. After 10 and 30 min, the mice were anesthetized with 2.5% isoﬂurane, and the distribution of EA2 was monitored by a live imaging system (X5, Tanon). The major organs (heart, liver, spleen, lung, kidney, and tumor) were excised and scanned. A Cy5-labeled random ssDNA was used as control.

**1.24 Development of EA2 aptamer-modified and PTX/LUT-co-loaded PSL**

Motivated by our previous successful development of aptamer-conjugated nanocarriers for targeted drug delivery to ESCC cells, we constructed pH-responsive liposomes modified with functional aptamers on the surface for the dual delivery of PTX and LUT. Liposomes were engineered using the thin-film hydration technique, and their formulation compositions were screened. Briefly, a mixture of DOPE, CHEMS, and TPGS (optimized molar ratio: 6:4:0.25, total lipids molar: 102.5 mmol) served as the liposome carrier. This lipid mixture was dissolved in a mixed organic solvent of methanol and dichloromethane (10 mL, 1:1.5, v/v). Then, 6.40 mmol LUT and 0.64 mmol PTX were introduced, resulting in the formation of a clear and transparent solution through ultrasonic treatment at 25 °C. The removal of the organic solvent was carried out at 40 °C using a rotary evaporator (Heidolph Co., Germany), leaving a thin ﬁlm complex at the bottom of the flask, which was then desiccated overnight in a vacuum drier. Subsequently, 10 mL of PBS was incorporated, and the desiccated mixture complex was fully hydrated with magnetic stirring in a water bath at 37 °C for 1 h. The mixture underwent low-temperature sonication for 6 min (1 s on, 1 s off, total sonication power of 130 W, sonication power of 60%). The resulting solutions were centrifuged at 10,000 *g* for 30 min to remove the unencapsulated PTX and LUT. Finally, the solution was filtered through a 0.45 μm nylon membrane to obtain the liposome solution (PSL-PTX/LUT). For aptamer modification, 6.6 μg PEI was introduced into 100 μL PSL-PTX/LUT and vibrated at room temperature for 1 h. The resulting cationic liposomes were sonicated every 2 min three times. Then, 250 pmol EA2 was vibrated with PSL-PTX/LUT for 30 min at 4 °C and subsequently bath-sonicated every 2 min three times to acquire EA2-modified liposome (EA2-PSL-PTX/LUT). Blank, PTX-loaded, and LUT-loaded liposomes were prepared following the aforementioned protocol. The final formulations were stored at 4 °C.

**1.25 Characterization of EA2-PSL-PTX/LUT**

To confirm the successful conjugation of the EA2 to PSL-PTX/LUT, agarose gel electrophoresis and fluorescence emission spectra analysis were performed. Samples, including the DNA ladder, free EA2, PSL-PTX/LUT, a mixture of EA2 and PSL-PTX/LUT, and EA2-PSL-PTX/LUT, were mixed with loading buffer and subjected to electrophoresis at 100 V for 90 min using 0.5 tris-borate-EDTA (TBE) buffer as the running buffer. Results were visualized using a Bioimage system (UVP, GelDoc-It2 Imager). Typical fluorescence spectra of FAM-labeled EA2-PSL-PTX/LUT and PSL-PTX/LUT were recorded using an Edinburgh FLS1000 fluorescence spectrometer.

**1.26 Entrapment eﬃciency (EE) and drug loading (DL)**

The EE and DL of EA2-PSL-PTX/LUT were estimated using an ultrafiltration-centrifugation method. Briefly, 200 μL of EA2-PSL-PTX/LUT was placed in a 0.5 mL ultrafiltration tube (MWCO, 100,000 Da, Millipore), then centrifuged at 4 °C, 10000 rpm for 10 min. The quantities of PTX and LUT in the filtrate were regarded as the non-encapsulated drug content. Concurrently, 200 μL of EA2-PSL-PTX/LUT was ruptured by methanol, and subjected to ultrasonic treatment to liberate PTX and LUT, which was considered the total content of PTX and LUT. Then, the concentration of free PTX or free LUT (*W*_non-encapsulated_) and the initial total PTX or total LUT (*W*_total_) were determined using high-performance liquid chromatography (HPLC) method. The excipient weight (*W*_exc_) was calculated from the preparation. The EE% and DL% of EA2-PSL-PTX/LUT were calculated using the following formulas: EE (%) = (*W*_total_ – *W*_non-encapsulated_) / *W*_total_ × 100% and DL (%) = (*W*_total_ – *W*_non-encapsulated_) / (*W*_total_ – *W*_non-encapsulated_ + *W*_exc_) × 100%. The EE and DL of all the samples were determined in triplicate.

The quantification of PTX and LUT was performed by HPLC (Agilent 1260, USA) through a Waters symmetry C 18 column (4.6 mm × 150 mm, pore size 3.5 μm) eluted at a flow rate of 1 mL/min. The column temperature was maintained at 35 °C, and a sample volume of 20 μL was injected. PTX was quantified at 227 nm using a mobile phase comprising methanol and water (72/25, v/v). Conversely, the chromatographic conditions for LUT were quantized using the same HPLC system but at 350 nm, with the mobile phase comprising a mixture of methanol and water (53/47, v/v) with 0.2% acetic acid. The standard curves of PTX and LUT were *Y* = 42.48 × *C* – 34.631 (linear range: 1–100 μg/mL, *R*^2^ = 0.9995), *Y* = 63.512 × *C* – 15.363 (linear range: 1–100 μg/mL, *R*^2^ = 0.9974), respectively, where *Y* refers to the peak area, and *C* denotes drug concentration. The precision, accuracy, and sensitivity of the analytical method were validated.

**1.27 Dynamic light scattering (DLS) and zeta potential**

For particle size and zeta potential analysis, an appropriate quantity of the prepared liposomes was diluted with PBS and subjected to laser diffraction and zeta potential analysis using a DLS instrument (PSS Nicomp 380 Z3000). Liquids were placed in cuvettes and examined at a 90° angle and 25 °C. Particle size and distribution, polydispersity index (PDI), and zeta potential were recorded and determined in triplicate.

**1.28 Transmission-electron microscopy (TEM)**

A droplet of diluted liposomes was deposited onto a dedicated copper grid to create a thin film and subsequently counterstained with acidic phosphotungstic (2%) solution for 30 s. The prepared thin films were air-dried for 30 min at ambient temperature prior to observation via TEM (7800HT, Hitachi, Japan).

**1.29 Stability**

Samples (500 μL each) were diluted using PBS and DMEM media with 10% FBS, respectively. Subsequently, they were stored for varying durations (1, 3, 7, 10, and 14 days) at 4 °C and 37 °C, and the particle size and PDI were evaluated for stability study.

*In vitro drug release:* The *in-vitro* pH-responsive drug release behavior of EA2-PSL-PTX/LUT was evaluated using the dialysis-bag method. Briefly, 1 mL aliquots of EA2-PSL-PTX/LUT were transferred into dialysis bags (Mol_wt._ = 3500 Da, 25 mm × 5 m; Spectrum Medical Industries Inc., USA). Subsequently, sealed bags were immersed in the different fresh dissolution media (PBS, pH = 7.4; and citrate buffer, pH = 5.4; 50 mL for each) that were maintained at 37 °C and stirred at 100 rpm. At predetermined times (0.5, 1, 2, 4, 8, 12, 24, 48, and 72 h), 1 mL samples were withdrawn and immediately replenished with an equal volume of preheated dissolution media. The quantities of released PTX and LUT were ascertained using the HPLC method described earlier, and *in-vitro* cumulative drug release profiles were plotted. All assessments were performed in triplicate.

**1.30 Hemolysis test**

Peripheral blood was extracted from C57BL/6 mice and centrifuged at 1500 g for 10 min at 4 °C to gather red blood cells (RBCs). RBCs were resuspended in PBS and mixed with varying concentrations of EA2-PSL-PTX/LUT. After incubating the mixture at 37 °C for 4 h, the supernatant was assayed for OD_450nm_ to determine the hemolysis rate, with 1% Triton-X 100 and PBS serving as positive and negative controls, respectively.

**1.31 Biological safety evaluation**

The biosafety of EA2-PSL-PTX/LUT was evaluated in KYSE-150 tumor-bearing mice. Mice were intravenously injected with PBS, combined PTX and LUT, PSL-PTX/LUT, and EA2-PSL-PTX/LUT (at same dosage of PTX at 2.5 mg/kg, LUT at 10 mg/kg) for once. At 1, 3, 5, and 7 days, blood samples were collected from retro-orbital bleeding for complete blood count. 14 days later, blood was collected for the biochemical measurements. Concurrently, major organs including the heart, liver, spleen, lung, kidney, and brain were collected for HE staining analysis, and weighed for determining the organ coefficients. Liver and kidney functions were evaluated by to measuring ALT, AST, AKP, LDH, UA, CRE, and BUN levels. Alterations in organ coefficients and histological staining were integrated to comprehensively assess the systemic toxicity *in vivo*.

**1.32 Cellular uptake**

Various C6-loaded liposomes were prepared using the aforementioned protocol. Circular glass slides were placed at the bottom of a 24-well cell culture plate, and KYSE-150 cells (2.5×10^4^ cells/500 μL) were individually seeded onto the slides and cultured overnight. After the cells were washed with PBS, culture media containing free C6, C6-loaded PSLs, and EA2-modified C6-loaded PSLs (with a final concentration of C6 was 5 μM) were added to different wells and incubated for 2 and 6 h. Subsequently, cells were thrice rinsed with cold PBS to arrest cellular uptake, fixed with 4% PFA, and counterstained with DAPI for 10 min. Fluorescence images were captured using LSCM. The same methodology was repeated, and the cells were trypsinized, centrifuged (300 g for 5 min), and resuspended in 500 µL cold PBS. The quantitative fluorescence intensity of the cells was determined by flow cytometry (BD C6 plus, USA) using FITC channel. To further evaluate the specific cellular uptake of EA2-modified PSLs, DiD-loaded PSLs and EA2-modified DiD-loaded PSLs were prepared. Various cancer cells (SiHa, U251, HCT-116, A549, CAL-27, FaDu, and KYSE-150 cells) were treated EA2-modified DiD-loaded PSLs for 1 h, then cells were harvested for flow cytometry (BD C6 plus, USA) using APC channel.

**1.33 Internalization mechanism study**

KYSE-150 cells (2×10^5^ cells/1000 μL) were individually seeded in a 12-well plate and incubated overnight. Subsequently, the cells were treated with different internalization inhibitors for 30 min at 37 °C, including genistein (inhibitor of caveolin-mediated endocytosis, 200 μM), chlorpromazine (inhibitor of clathrin-mediated endocytosis, 20 μM), colchicine (inhibitor of cytoskeleton-mediated endocytosis, 50 μM), nystatin (inhibitor of caveolin-mediated endocytosis, 5 μM), wortmannin (inhibitor of micropinocytosis, 20 μM), sodium orthovanadate (inhibitor of dynein, 10 μM), chloroquine (inhibitor of endosomal acidification, 100 μM), and cytochalasin D (inhibitor of phagocytosis, 20 μM). Following inhibitor treatment, the cells were incubated with EA2-modified C6-loaded PSLs for an additional 2 h. Subsequently, the cells were washed thrice with cold PBS, fixed with 4% PFA, and counterstained with DAPI. Representative fluorescence images were captured using an inverted fluorescent microscope. The same methodology was repeated, and the cells were collected for quantitative analysis using flow cytometry (BD C6 plus, USA).

**1.34 Endosomal escape study**

Cells were treated with the nanocarriers as described above. At predetermined times (2, 4, 6, and 12 h), cells were washed three times with PBS, and incubated with LysoTracker Red (2 μM) for 30 min. Subsequently, cells were fixed with 4% PFA and nuclei were stained with DAPI. Red fluorescence from LysoTracker and green fluorescence from C6 were captured and analyzed using LSCM.

**1.35 Targeting of EA2-modified PSLs**

Human peripheral blood mononuclear cells (PBMCs, obtained from Milestone Biological Scicnce & Technology Co. Ltd) were mixed with KYSE-150 cells at a ratio of 5:1 to establish a coculture model. The coculture model was incubated with DiD-loaded PSLs or EA2-modified DiD-loaded PSLs for 2 h at 37 °C and then stained with a PerCP anti-human CD45 antibody (Biolegend, 304025) for 30 min at 4 °C to mark PBMCs. The targeting capacity of EA2-modified PSLs to KYSE-150 cells was detected by flow cytometry (BD C6 plus, USA) using APC channel. Meanwhile, myeloid-derived suppressor cells (MDSCs) were used to evaluate the targeting capacity of EA2-modified PSLs in immunosuppressive microenvironment. Briefly, adherent monocytes from PBMCs were kept cultured in a surface‑treated 6‑well plate in the complete induction medium [RPMI‑1640 with 10% FBS, 10 ng/ml IL-6 (MCE, HY-P7044) and 10 ng/ml GM‑CSF (MCE, HY-P7016A)] for 7 days. The harvested MDSCs were mixed with KYSE-150 cells at a ratio of 5:1, treated with EA2-modified PSLs for 0.5 h, and stained with PE anti-human CD33 antibody (Biolegend, 366607) for flow cytometry.

To evaluate the off-toxicity of EA2-modified PSL, the coculture model of PBMCs with KYSE-150 cells was established as above, then PSL-PTX/LUT or EA2-modified PSL- PTX/LUT were added for 1 h-incubation. PBMCs were labelled with PerCP anti-human CD45 antibody, and a live/dead stain was used to identify dead cells. The cytotoxicity in KYSE-150 cells or PBMCs was detected by flow cytometry.

**1.36 Biodistribution of nanoparticles in blood cells**

Healthy human white blood cells (WBCs, obtained from Milestone Biological Scicnce & Technology Co. Ltd) were incubated with EA2-modified DiD-loaded PSLs for 0.5 and 2 h at 37 °C. The uptake of nanoparticles in WBCs was detected by flow cytometry using APC channel.

**1.37 *In vivo* biodistribution**

DiR was utilized as *in vivo* ﬂuorescent probe, the DiR-loaded PSLs and EA2-modified DiR-loaded PSLs were meticulously prepared. After the subcutaneous xenograft tumor model was established (tumor volume reached approximately 100 mm^3^), the mice were randomly assigned to groups. 100 µL DiR-loaded PSLs and EA2-modified DiR-loaded PSLs (same dosage of DiR was 1 μg) were intravenously injected into KYSE-150 tumor-bearing mice. At preselected time points (1, 2, 4, 6, 12, 24, 36, 48, 60, and 72 h), mice were anesthetized with 2.5% isoﬂurane, *in vivo* live imaging (X5, Tanon) was performed, and signal intensities in tumors were recorded. For biodistribution studies, mice were euthanized at 2, 24, and 72 h after injection, and the main organs, including the heart, liver, spleen, lungs, kidneys, brain, and tumor, were collected for live imaging scanning. The signal intensities were compared and analyzed for various organs.

**1.38 *In vivo* tumor penetration**

To analyze the accumulation of nanoparticles in tumors, KYSE-150 tumor bearing mice were intravenously injected with DiD-loaded PSLs and EA2-modified DiD-loaded PSLs (same equivalent dosage of DiD, 1 μg). After 24 h, the mice were sacrificed, and the harvested tumors were frozen-sectioned for further experiments. The nuclei were stained with DAPI, tumor cells were stained with FITC-tagged EpCAM, followed by observation under LSCM.

**1.39 Biodistribution of nanoparticles in tumors via flow cytometry assay**

To evaluate the number of different nanoparticles in tumor cells, tumor tissues were dissociated for flow cytometry. Briefly, tumor tissues were sliced into 3 × 3 × 3 mm cubes and enzymatically dissociated using 1 mg/mL type I collagenase (Worthington, LS004194), 1 mg/mL type IV collagenase (Worthington, LS004186), and 50 U/mL DNase I (Roche, 10104159001) for 40 min at 37 °C to obtain single-cell suspensions. Cellular surface staining was conducted for 30 min on ice, including PE/Cyanine7-CD45 (BioLegend, 103113), PE-EpCAM (BioLegend, 324205). Afterwards, the cells were washed with PBS three times, and the stained cells were analyzed using flow cytometry (URIT bf-730, Guilin Ulead Medical Electronics Co., Ltd, China). The data were analyzed using Kaluza software.

**1.40 Immune cell recruitment ability**

The *in vitro* co-culture model was constructed with KYSE-150 cells and PBMCs using Transwell (as depicted in **Figure S21a**). Briefly, a total of 3×10^4^ DiR-labeled PBMCs were added to the upper chamber. 1×10^5^ CFSE-labeled KYSE-150 cells with or without 20 ng/mL CCL-2, 250 nM EA2, PSL-PTX/LUT, and EA2- PSL-PTX/LUT were added to the lower insert medium, respectively. The chemotactic efficiency of PBMCs was determined by DiD fluorescence in the lower chamber using LSCM.

**1.41 *In vitro* therapeutic efficacy**

Various treatments were prepared, including PBS, PTX, LUT, PTX combined with LUT, PSL-PTX, PSL-LUT, PSL-PTX/LUT, and EA2-PSL-PTX/LUT. All treatments were formulated at equimolar doses, specifically 7.5 nM for PTX and 15 μM for LUT. To evaluate the cytotoxic effects of various nanomedicines on KYSE-150 cells, cell proliferation, migration, and invasion were assessed using the CCK-8 assay, colony formation assay, EdU staining, wound-healing assay, and Transwell assay, as previously described. For 3D tumor sphere growth analysis, single-cell suspensions of KYSE-150 cells (6000 cells/mL) were combined with pre-chilled Matrigel on ice, seeded into a 15 mm confocal dish, and cultured for approximately a week until tumor spheroids formed. Subsequently, treatments with various nanomedicines were administered every 3 days for a total of five rounds. The sizes of the resulting tumor spheroids were recorded.

**1.42 *In vivo* anti-tumor efficacy**

A subcutaneous xenograft tumor model was established using luciferase-expressing KYSE-150 cells (5×10^6^/100 μL) in BALB/c nude mice. When the tumor volume reached approximately 100 mm^3^, the mice were randomly assigned to groups: PBS, PTX plus LUT, PSL-PTX/LUT, and EA2-PSL-PTX/LUT. The mice were intravenously injected with equimolar doses of PTX (2.5 mg/kg), LUT (10 mg/kg), or other nanomedicines. Treatments were administered every 7 days for five cycles. Body weight was recorded every 3 days, and a weight curve was plotted. Tumor dimensions were measured using calipers, and tumor volume was calculated to create a tumor growth curve. Following the final treatment, mice were intraperitoneally injected with d-luciferin (150 mg/kg), KYSE-150-derived subcutaneous tumors were monitored using a live imaging system, and the fluorescence intensity of the tumor was recorded. Subsequently, the mice were sacrificed, and the tumor tissues were removed, weighed, and photographed. Tumors were dissected, fixed in 4% PFA, and sectioned. HE staining and TUNEL staining were performed to assess tumor cell apoptosis, and Ki67 staining was conducted to analyze tumor cell proliferation.

**1.43 *In vivo* remodeling effects of EA2-PSL-PTX/LUT on TME**

Human immune microenvironment was reconstructed in NCG mice. Briefly, cryopreserved human PBMCs were purchased commercially from Milestone Biological Scicnce & Technology Co. Ltd. PBMCs were washed twice with PBS after thawing, then, injected intravenously into NCG mice (10^7^/100 μL). After 3 days of PBMCs engrafting, Luciferase-expressing KYSE-150 cells were subcutaneously inoculated into PBMCs-engrafted mice (5×10^6^/100 μL) to establish a subcutaneous xenograft tumor model. After 14 days, whole blood was collected for flow cytometry assay, and human immune cell populations were monitored using a PerCP anti-human CD45 antibody (Biolegend, 304025). PBMCs-engrafted NCG mice bearing KYSE-150 tumors (hCD45>20%, and tumor volume> 100 mm^3^) were randomized blindly into 3 treatment groups, PBS, free PTX plus LUT, and EA2-PSL-PTX/LUT groups. The mice were intravenously injected with equimolar doses of PTX (2.5 mg/kg), LUT (10 mg/kg), for every 3 days for five cycles. Mice were sacrificed on day 15 to analyze tumor microenvironment.

The relative frequency of various immune cell subsets including dendritic cells (DCs), T cells, MDSCs, regulatory T cells (Tregs), and tumor-associated macrophages (TAMs), were examined by flow cytometry analysis as described above. The antibodies included the following: PerCP anti-human CD45 (BioLegend, 304026), Alexa Fluor 488 anti-human CD3 (BioLegend, 300415), Brilliant Violet 605™ anti-human CD4 (BioLegend, 317437), Brilliant Violet 785™ anti-human CD8 (BioLegend, 344739), Brilliant Violet 421™ anti-human Granzyme B (BioLegend, 396413), Brilliant Violet 785™ anti-human CD11b (BioLegend, 101243), PE anti-human CD68 (BioLegend, 333808), Brilliant Violet 510™ anti-human CD86 (BioLegend, 305432), Alexa Fluor 488 anti-human CD206 (BioLegend, 321114), Alexa Fluor 647 anti-human CD25 (BioLegend, 356128), Brilliant Violet 421™ anti-human FOXP3 (BioLegend, 320123), Brilliant Violet 650™ anti-human HLA-DR (BioLegend, 307649), APC anti-human CD11c (BioLegend, 371505), Brilliant Violet 605™ anti-human CD80 (BioLegend, 305225), PE anti-human CD33 (BioLegend, 366607). The stained cells were analyzed using flow cytometry (URIT bf-730, Guilin Ulead Medical Electronics Co., Ltd, China). The data were analyzed using Kaluza software.

The immunofluorescence analysis was further used to evaluate the infiltration of DC cells (CD11c^+^), T cells (CD8^+^/CD4^+^), inhibition of MDSCs (CD33^+^), and softening of stroma barrier (α-SMA/Collagen I) in the ESCC region. Staining was performed with primary CD11c, CD8, CD4, CD33^+^, α-SMA and Collagen I antibodies at a dilution ratio of 1:100. The sections were further stained with corresponding secondary antibodies and DAPI, respectively. Fluorescence images were captured and analyzed using LSCM.

**2 Supporting Figures**


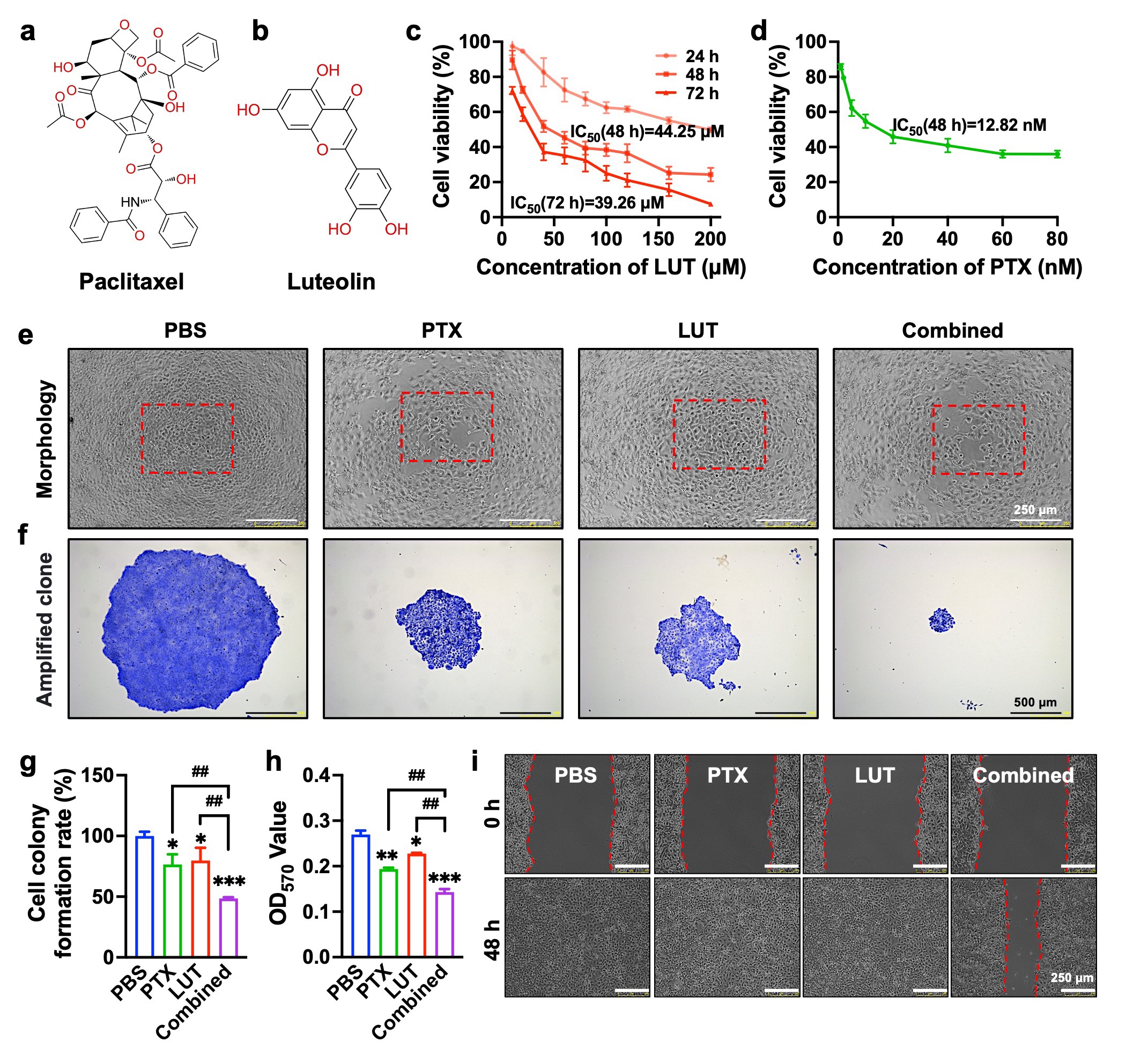


**Figure S1. LUT and PTX exerts a remarkable synergistic inhibition on ESCC *in vitro*. a-b.** Chemical structure of PTX and LUT. **c**. Inhibition effects of LUT on cell viability in KYSE-150 cells. Cells were treated with different concentrations of LUT (10, 20, 40, 60, 80, 100, 120, and 200 μM) for 24, 48, or 72 h, then CCK-8 assay was performed to determine cell viability, and IC_50_ was calculated. **d**. Cell viabilities of KYSE-150 cells treated with varying concentrations of PTX (1, 2, 5, 10, 20, 40, 60, and 80 nM) for 48 h, and IC_50_ was calculated. **e**. Representative morphological changes magnified at 100 ×. Scale bars, 250 μm. **f**. Representative single clone magnified at 200 ×. Scale bars, 500 μm. **g**. Effects of combined PTX and LUT on colony formation rate. **h**. Effects of combined PTX and LUT on cell invasion of KYSE-150 cells using Transwell assays. Invaded cells were stained with crystal violet, and then destained using DMSO for detecting OD value at 570 nm. **i**. Representative images captured at 0 and 48 h in wound-healing assay, Scale bars, 250 μm. All data expressed as mean ± SD (n=5), statistical significance between different groups was obtained by one-way ANOVA using the Tukey’s post-test (**g**, **h**). ^*^ *p*<0.05, ^**^ *p*<0.01, ^***^ *p*<0.001, significant as compared to the PBS group. ^##^ *p*<0.01, ^###^ *p*<0.001, significant as compared to PTX or LUT group.


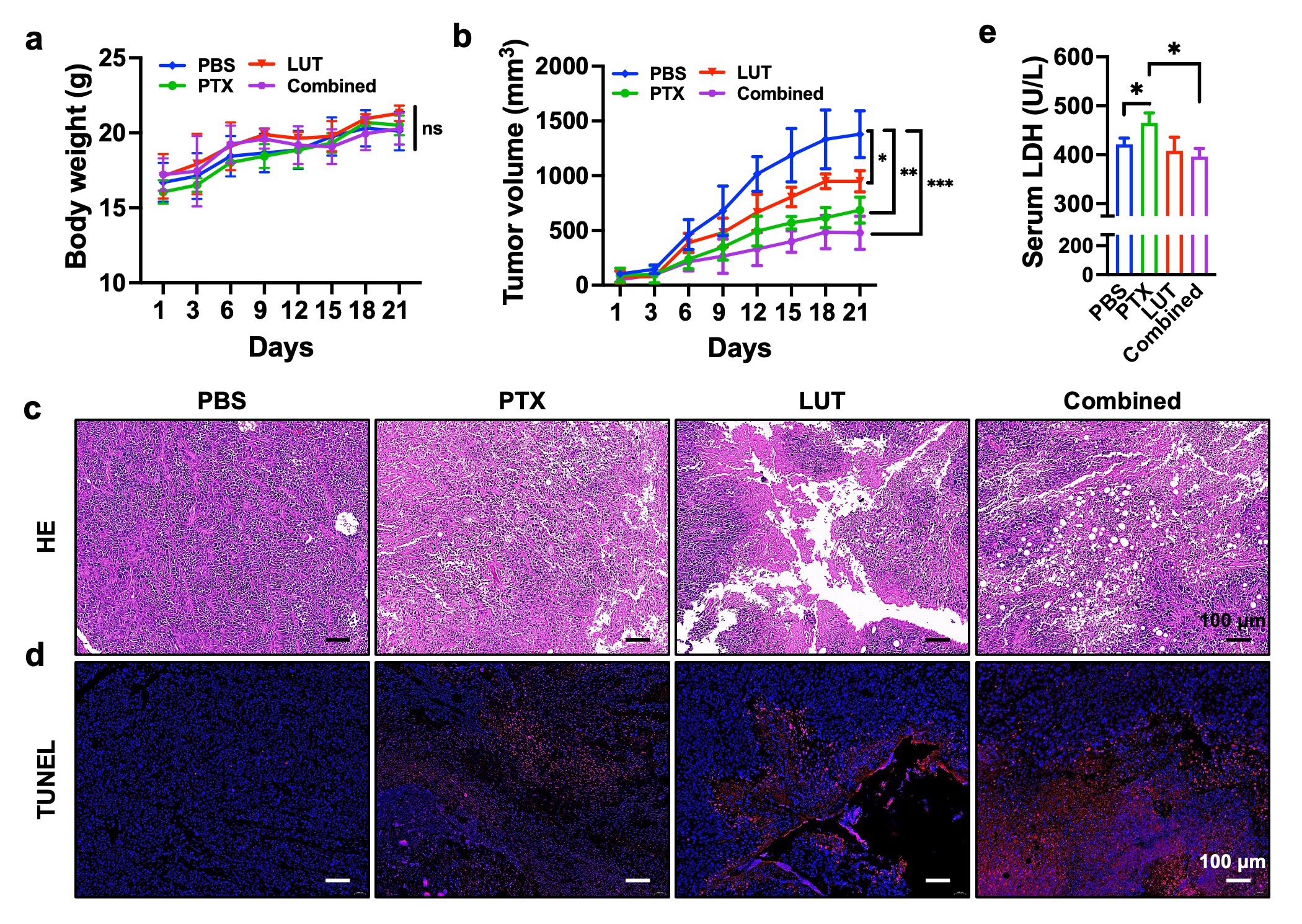


**Figure S2. LUT and PTX exerts a remarkable synergistic inhibition on ESCC *in vivo*.** **a**. Body weight change of the nude mice during various treatments, including PBS, PTX (5 mg/kg), LUT (50 mg/kg), and Combined (PTX, 5 mg/kg and LUT, 50 mg/kg). **b**. Tumor volume changes measured every 3 days of KYSE-150 tumor-bearing mice under different treatments. **c-d**. Representative micrographs of HE and TUNEL staining of tumor tissues. Scale bars, 100 μm. **e**. Serum LDH leakage after different treatments. All data expressed as mean ± SD (n=3), statistical significance between different groups was obtained by one-way ANOVA using the Tukey’s post-test (**a**, **b**, **e**). ^*^ *p*<0.05, ^**^ *p*<0.01, ^***^ *p*<0.001, indicating statistical significance between the compared groups.


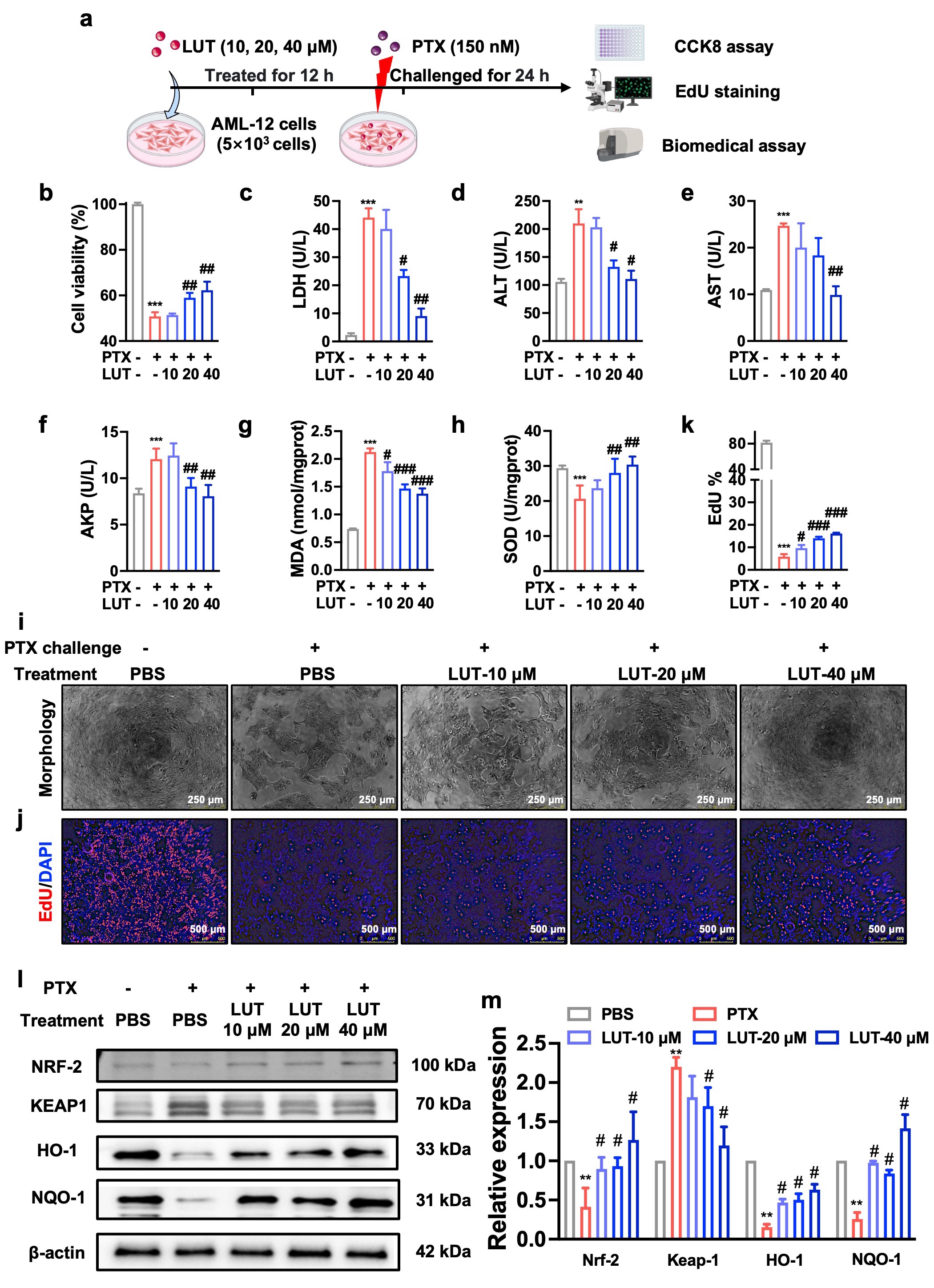


**Figure S3.** **LUT alleviates the PTX-induced hepatotoxicity in the AML12 cell.** **a**. Systemic illustration of hepatoprotective effects of LUT against PTX-induced injury. **b**. Cytoprotective effects of LUT on cell viabilities in AML12 cells subjected to PTX-induced damage. AML12 cells were preincubated with LUT (10, 20, and 40 μM) for 12 h, followed by PTX exposure (150 nM) for 24 h, cell viability was determined using CCK-8 assay. **c**. LUT decreased LDH leakage in the cell supernatant of PTX-treated AML12 cells. **d-f**. ALT, AST, and AKP levels in the cell supernatant. **g-h**. Intracellular oxidative damage (MDA and SOD). **i**. Effects of LUT on the cellular morphological alterations in PTX-treated AML12 cells. Scale bars, 250 μm. **j-k**. Cell proliferation in AML12 cells was increased by LUT treatment using EdU assay. Red: EdU positive cells; blue: cell nuclei. Percentage of EdU^+^ proliferating cells calculated by ImageJ. Scale bars, 500 μm. **l-m**. Protein expression of Nrf2 and Keap1, HO-1 and NQO-1 in PTX-treated in AML12 cells. All data expressed as mean ± SD (n=5), statistical significance between different groups was obtained by one-way ANOVA using the Tukey’s post-test (**b**, **c**, **d**, **e**, **f**, **g**, **h**, **k**, **m**). ^*^ *p*<0.05, ^**^ *p*<0.01, ^***^ *p*<0.001, significant as compared to the PBS only group. ^#^ *p*<0.05, ^##^ *p*<0.01, ^###^ *p*<0.001, significant as compared to PTX only group.


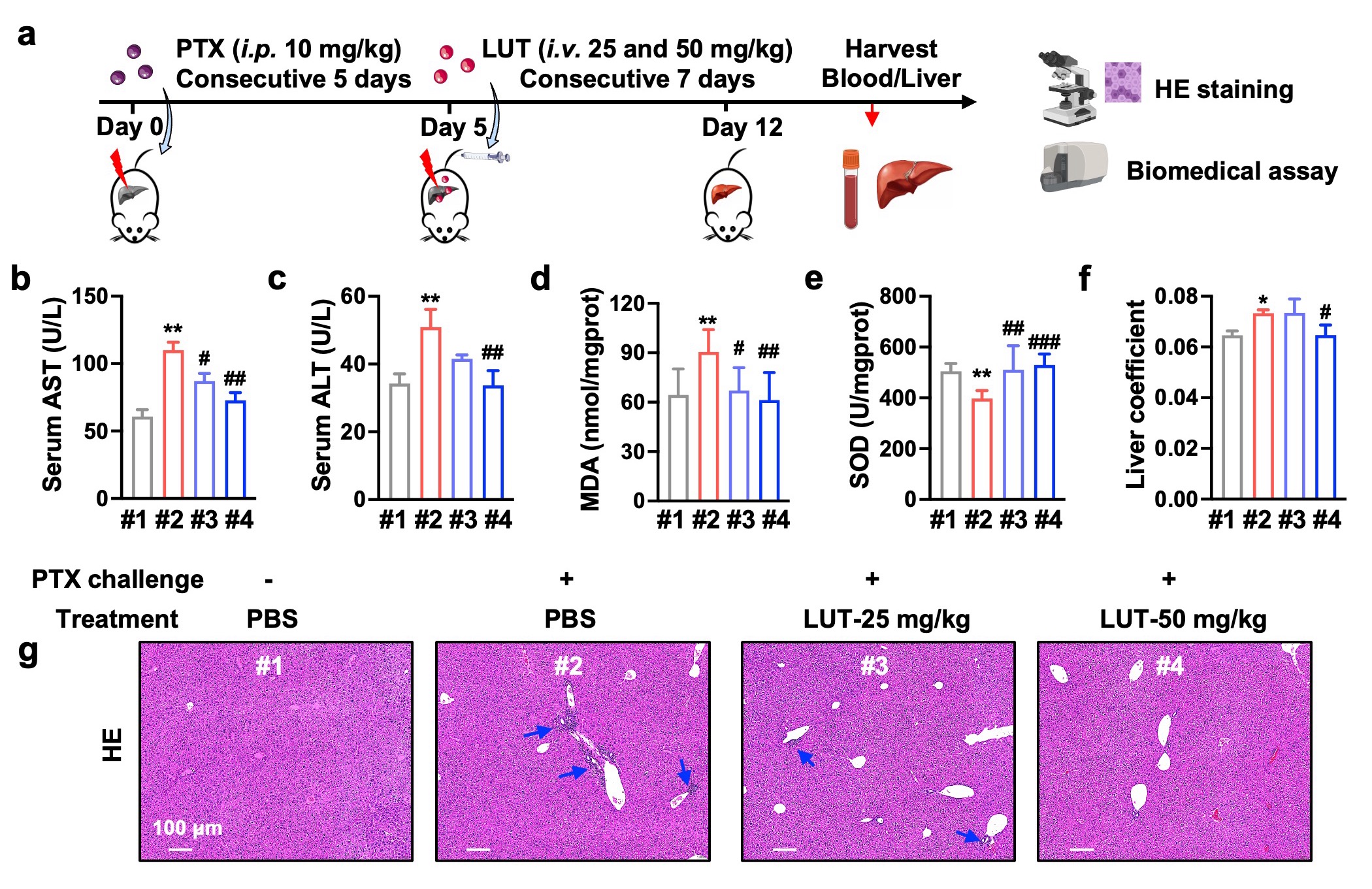


**Figure S4.** **LUT alleviates the PTX-induced hepatotoxicity in C57BL/6J mice.** **a**. Schematic illustration of PTX-induced liver injury and treatment of LUT in mice. Hepatotoxicity was induced in C57BL/6J mice by intraperitoneal injection of PTX (10 mg/kg) for 5 consecutive days. Groups included PBS only group (#1), PTX-injured group without LUT treatment (#2), PTX-injured group receiving 25 mg/kg LUT treatment (#3), and PTX-injured group receiving 50 mg/kg LUT treatment (#4). Following treatment with LUT for 7 consecutive days, mice were sacrificed, and the serum and liver were collected. Levels of ALT and AST in the serum, as well as MDA and SOD in the liver, were determined. **b-c**. Serum hepatic function indicators (AST and ALT). **d-e**. Oxidative damage (MDA and SOD) in liver tissues. **f**. Effects of LUT on liver index. **g**. Representative HE staining of liver tissues. Images were captured by inverted optic microscope, blue arrows: inflammation. Scale bars, 100 μm. All data expressed as mean ± SD (n=3), statistical significance between different groups was obtained by one-way ANOVA using the Tukey’s post-test (**b**, **c**, **d**, **e**, **f**). ^*^ *p*<0.05, ^**^ *p*<0.01, significant as compared to the PBS only group. ^#^ *p*<0.05, ^##^ *p*<0.01, ^###^ *p*<0.001, significant as compared to PTX only group.


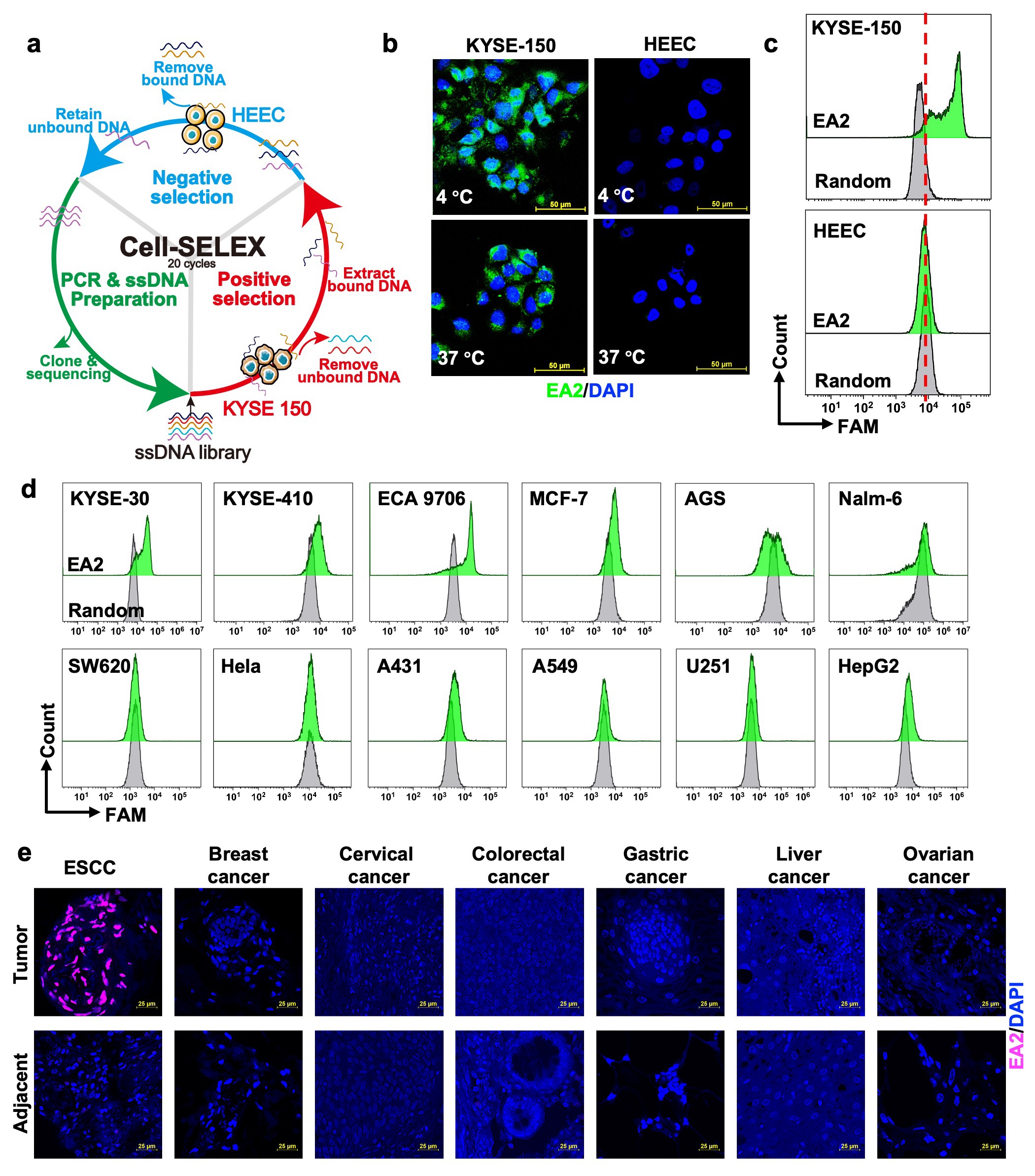


**Figure S5.** **Screening, affinity, and specificity characterization of aptamer EA2.** **a**. Schematic diagram of cell-SELEX process. **b**. Binding specificity of FAM-labeled EA2 with KYSE-150 and HEEC cells at 4 °C and 37 °C using confocal imaging. Blue: cell nuclei; green: EA2. Scale bars, 50 μm. **c**. Binding aﬃnity of FAM-labeled EA2 (250 nM) to KYSE-150 and HEEC cells by flow cytometry assays. FAM-labeled random ssDNA was used as control, and fluorescence signals were recorded through the FAM channel. **d**. Binding selectivity assays of the FAM-labeled EA2 to different cancer cell lines by flow cytometry, including KYSE-30, KYSE-410, ECA-9706, MCF-7, AGS, Nalm-6, SW620, HeLa, A431, A549, U251, and HepG2. **e**. Confocal imaging to detect the binding specificity of Cy5-labeled EA2 with ESCC tissue and adjacent non-tumor tissue, as well as various other tumor tissues (breast, cervical, colorectal, gastric, liver, and ovarian cancers). The Cy5 fluorescence signal was collected at excitation of 649 nm and emission of 670 nm. Blue indicates cell nuclei, and pink indicates EA2 binding.


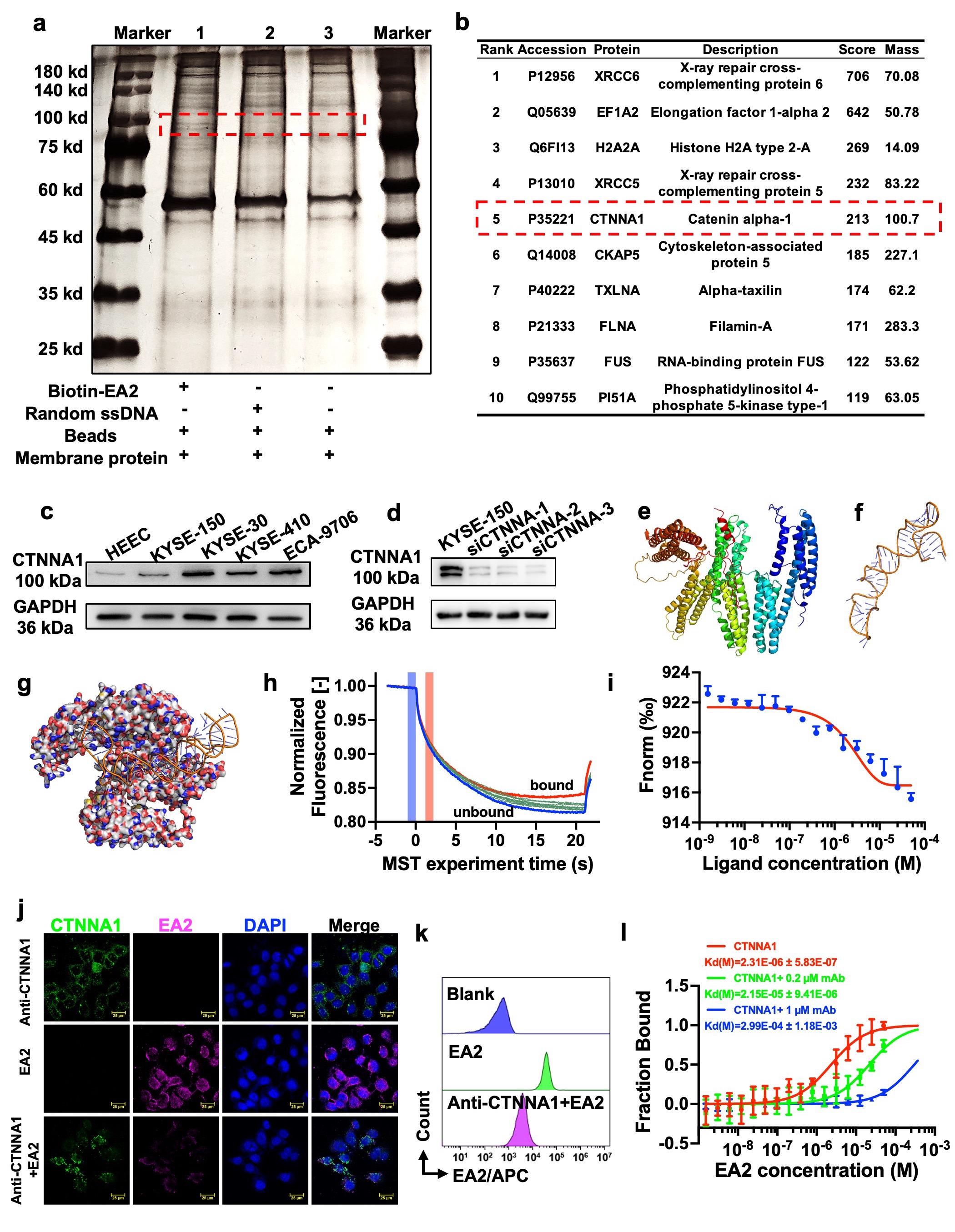


**Figure S6. The identification of CTNNA1 being the binding target of EA2**. **a**. Differential band analysis by SDS-PAGE after ssDNA pull-down assays. **b**. CTNNA1 was identified as the binding target of EA2 by mass spectrometry detection and comparative analysis using Proteome Discovery 1.3, the top 10 proteins based on scores were listed. **c**. Relatively protein expression of CTNNA1 by Western blotting analysis in normal human esophageal epithelial cells (HEEC) and various ESCC cell lines, including KYSE-150, KYSE-30, KYSE-410, and ECA9706. **d**. Knockdown of CTNNA1 in KYSE-150 cells using siRNAs. Total proteins of the treated cells were subjected to Western blotting. **e**. 3D protein structure of CTNNA1 retrieved from the AlphaFold database. **f**. 3D structure of aptamer EA2 using RNAcomposer. **g**. Computational model and interactions of CTNNA1 and EA2 complex. **h**. The fluorescence time traces and fluorescence responses of NHS-labeled CTNNA1 in the presence of varying concentrations of EA2 (ranging from 50 µM to 0.00153 µM) in MST analysis. **i**. Dose-response curves for EA2 against CTNNA1. Dose-response curves were fitted to a one-site binding model to obtain *K*_d_ values. Error bars represent the standard deviation. MST experiments were conducted with an LED power setting of 60% and at moderate MST power. Fnorm denotes normalized fluorescence. **j**. Competitive binding of Cy-5 labelled EA2 (Magenta) and FITC labelled CTNNA1 antibody (Green) in KYSE-150 cells. **k**. Competitive binding of EA2 and CTNNA1 antibody in KYSE-150 cells using flow cytometry. **l**. Kinetic constant (K_d_) analyses of CTNNA1 interacting with EA2 under the competitive binding of CTNNA1 antibody using MST experiments.


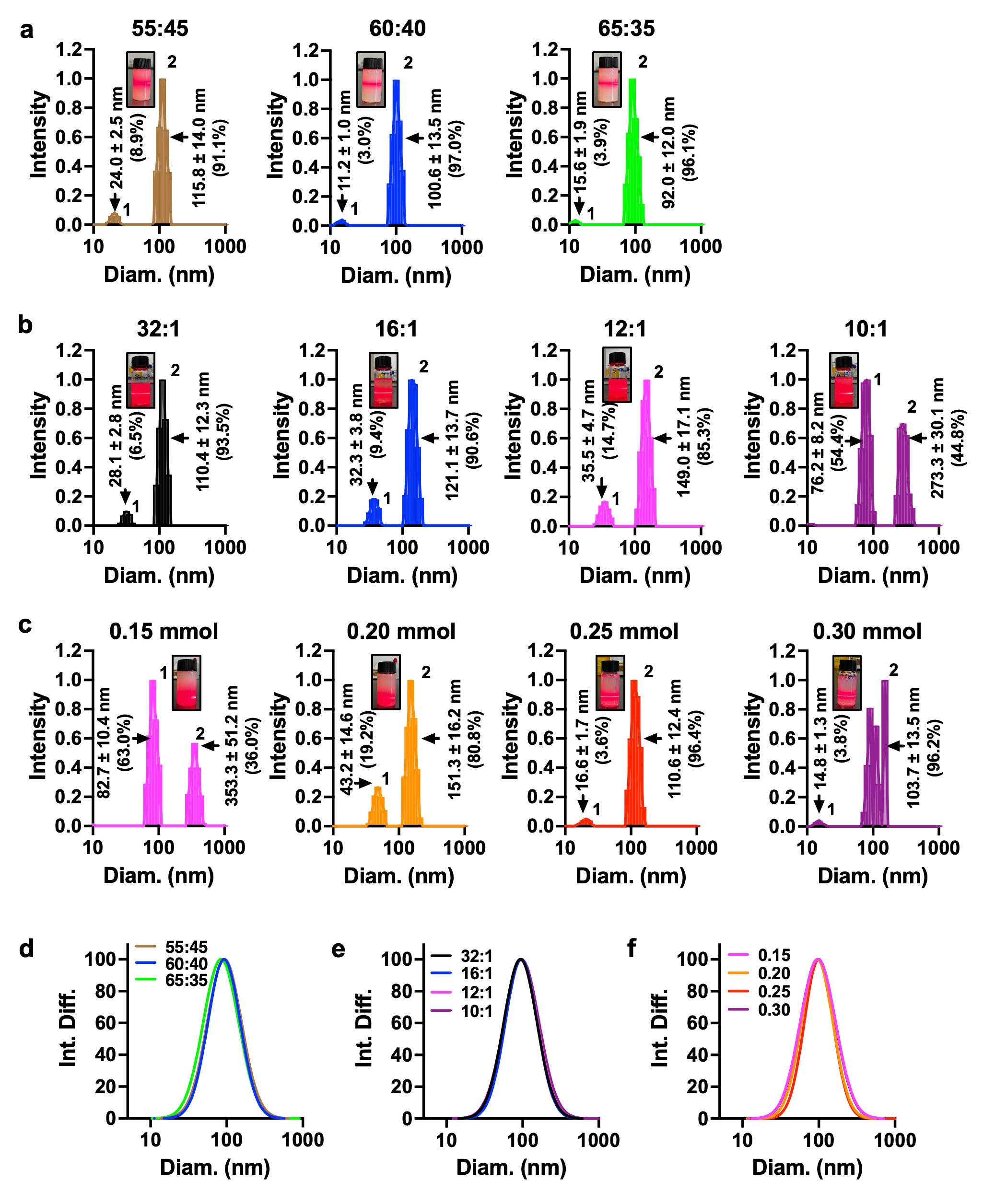


**Figure S7. Formulation optimization of PSL-PTX/LUT**. Particle size distributions and Gaussian distributions were employed for optimization, involving screening of the molar ratios of DOPE to CHEMS (**a, d**), drug-to-lipid molar ratio (**b, e**), and TPGS addition (**c, f**).

**
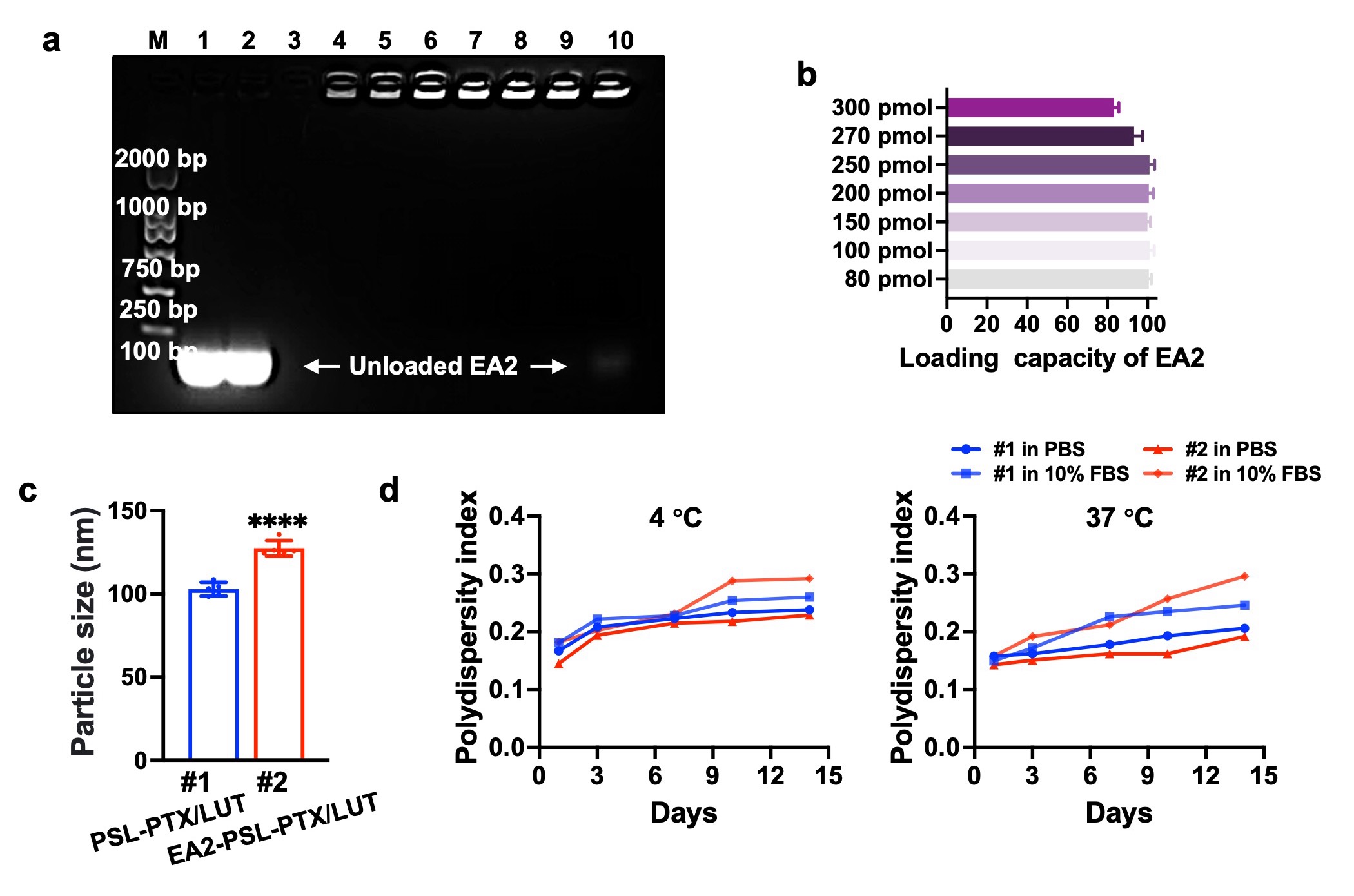
**

**Figure S8. Physicochemical characterizations of EA2-PSL-PTX/LUT. a**. 2% Agarose electrophoresis assay of PSL-PTX/LUT incorporating varying amounts of EA2. The ratio was maintained at 100 μL PSL-PTX/LUT: 6.6 μg PEI, with different quantities of EA2 added. Conjugation aptamer EA2 to PSL-PTX/LUT was identified at 250 pmol EA2. M: DNA ladder, lane 1: free EA2, lane 2: simple mixture of PSL-PTX/LUT and EA2, lane 3: PSL-PTX/LUT, lane 4: 80 pmol EA2, lane 5: 100 pmol EA2, lane 6: 150 pmol EA2, lane 7: 200 pmol EA2, lane 8: 250 pmol EA2, lane 9: 270 pmol EA2, lane 10: 300 pmol EA2. **b**. Loading capacity of aptamer EA2 to PSL-PTX/LUT. **c**. Mean particle size of PSL-PTX/LUT (#1) and EA2-PSL-PTX/LUT (#2). **d**. Stability assessment using PDI in PBS and DMEM supplemented with 10% FBS at 4 and 37 °C. All data expressed as mean ± SD (n=3), statistical significance between different groups was obtained by an unpaired two-tailed Student’s t test (**c**), ^***^ *p*<0.001, indicating statistical significance between the compared groups.


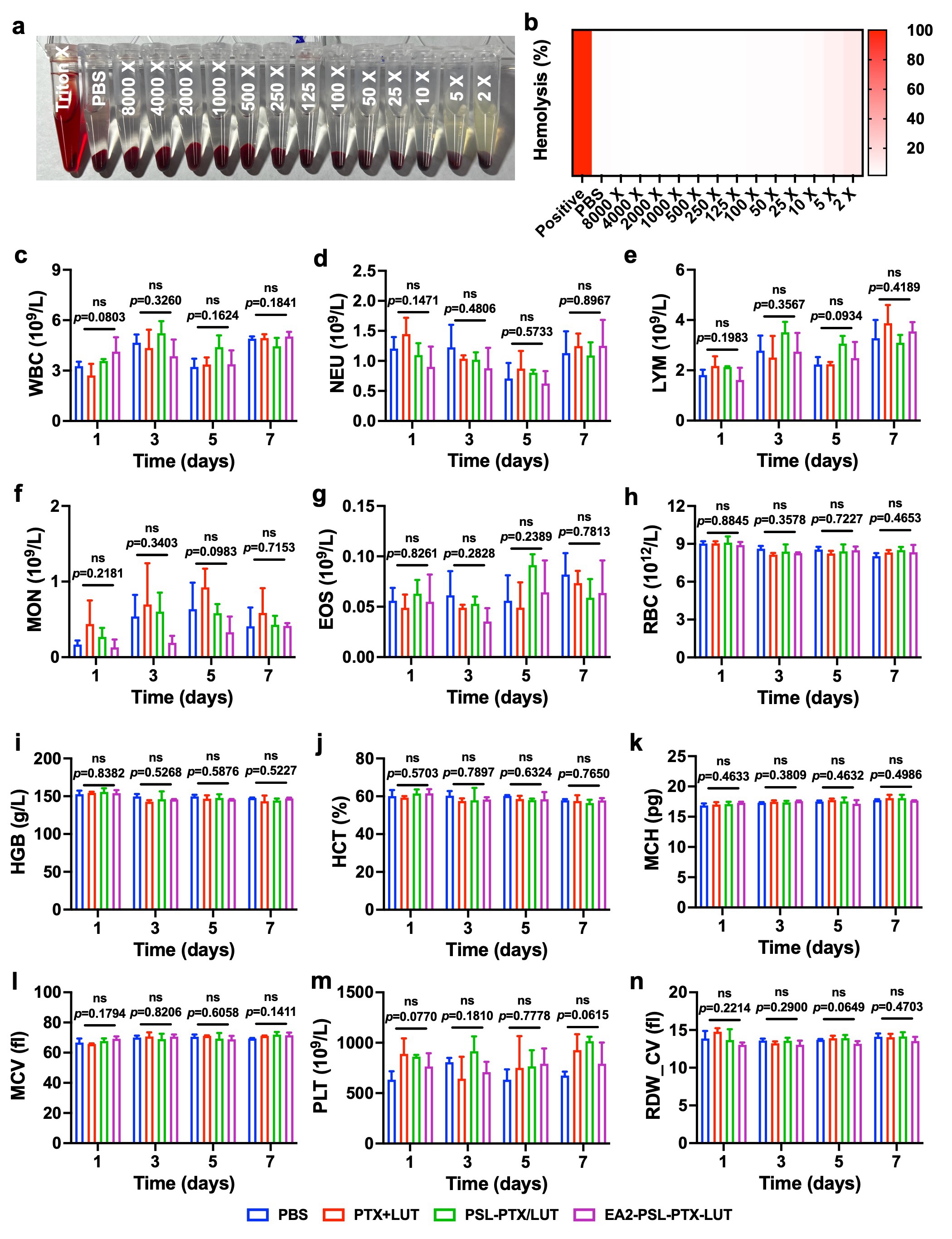


**Figure S9. *In vivo* hematological toxicity of EA2-PSL-PTX/LUT**. **a-b**. Hemolysis evaluation of EA2-PSL-PTX/LUT at various dilutions. **c-n**. Blood routine analysis. KYSE-150 tumor-bearing mice were treated with PBS, free PTX and LUT, PSL-PTX/LUT, and EA2-PSL-PTX/LUT at equimolar doses of PTX (2.5 mg/kg) and LUT (10 mg/kg). Complete blood cell counts, and differential white blood cell counts were performed to evaluate the hematological toxicity. WBC, white blood cell; NEU, neutrophils; LYM, lymphocytes; MON, monocytes; EOS, eosinophils; RBC, red blood cells; HGB, haemoglobin; HCT, hematocrit; MCH, mean corpuscular hemoglobin; MCV, mean corpuscular volume; PLT, platelets; RDW_CV, red blood cell distribution width. All data expressed as mean ± SD (n=3), statistical significance between different groups was obtained by one-way ANOVA using the Tukey’s post-test (**c-n**).


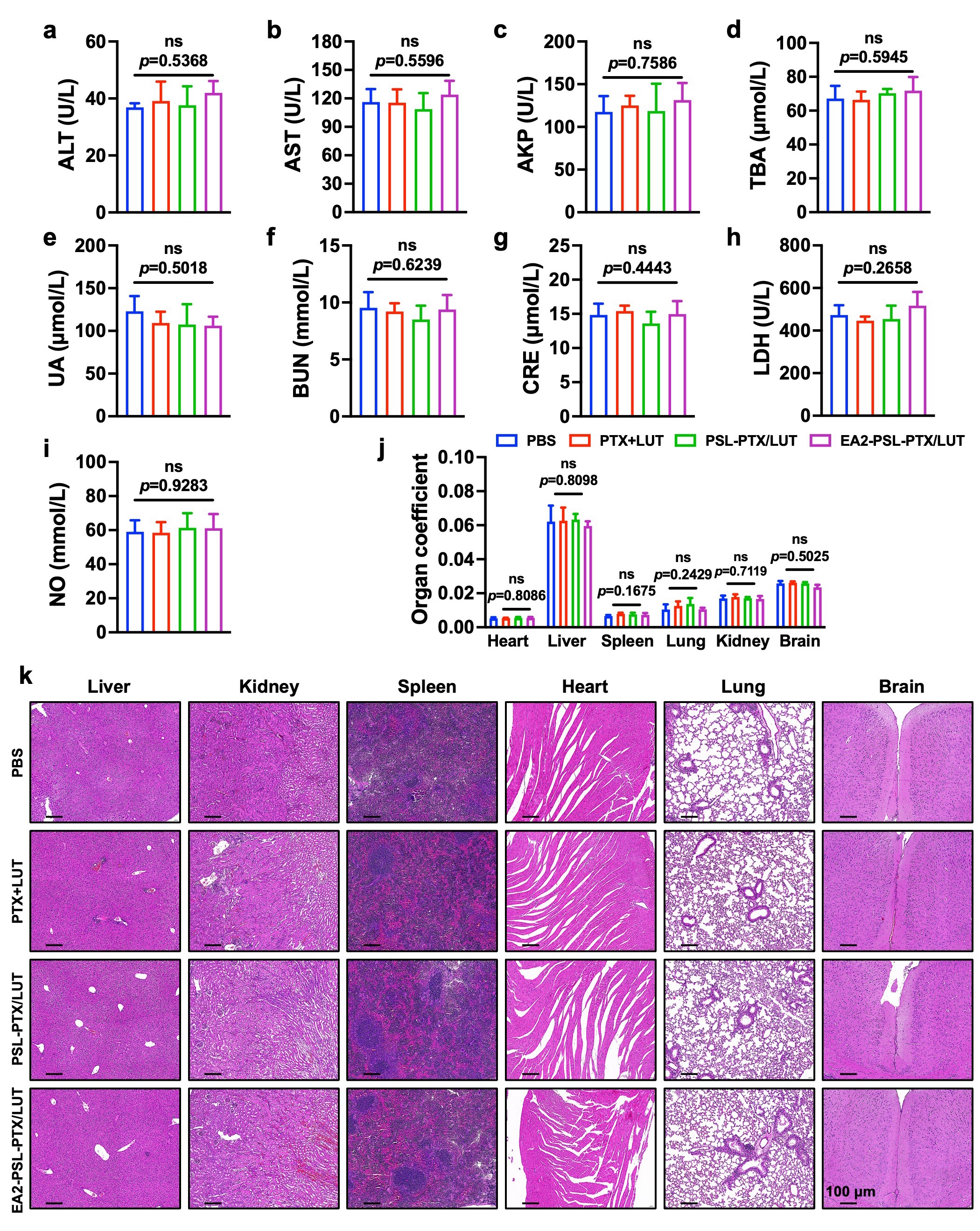


**Figure S10. *In vivo* biocompatibility of EA2-PSL-PTX/LUT**. **a-d**. Serum hepatic function indicators (ALT, AST, AKP, and TBA). **e-g** Serum renal function indicators (UA, BUN, and CRE). **h**. Tissue damage marker (LDH). **i**. Serum immune response index (NO). **j**. Main organ coefficients. **k**. Representative HE staining images of liver, kidney, spleen, heart, lung, and brain tissues. Scale bars, 100 μm. All data expressed as mean ± SD (n=3), statistical significance between different groups was obtained by one-way ANOVA using the Tukey’s post-test (**a-j**).

**
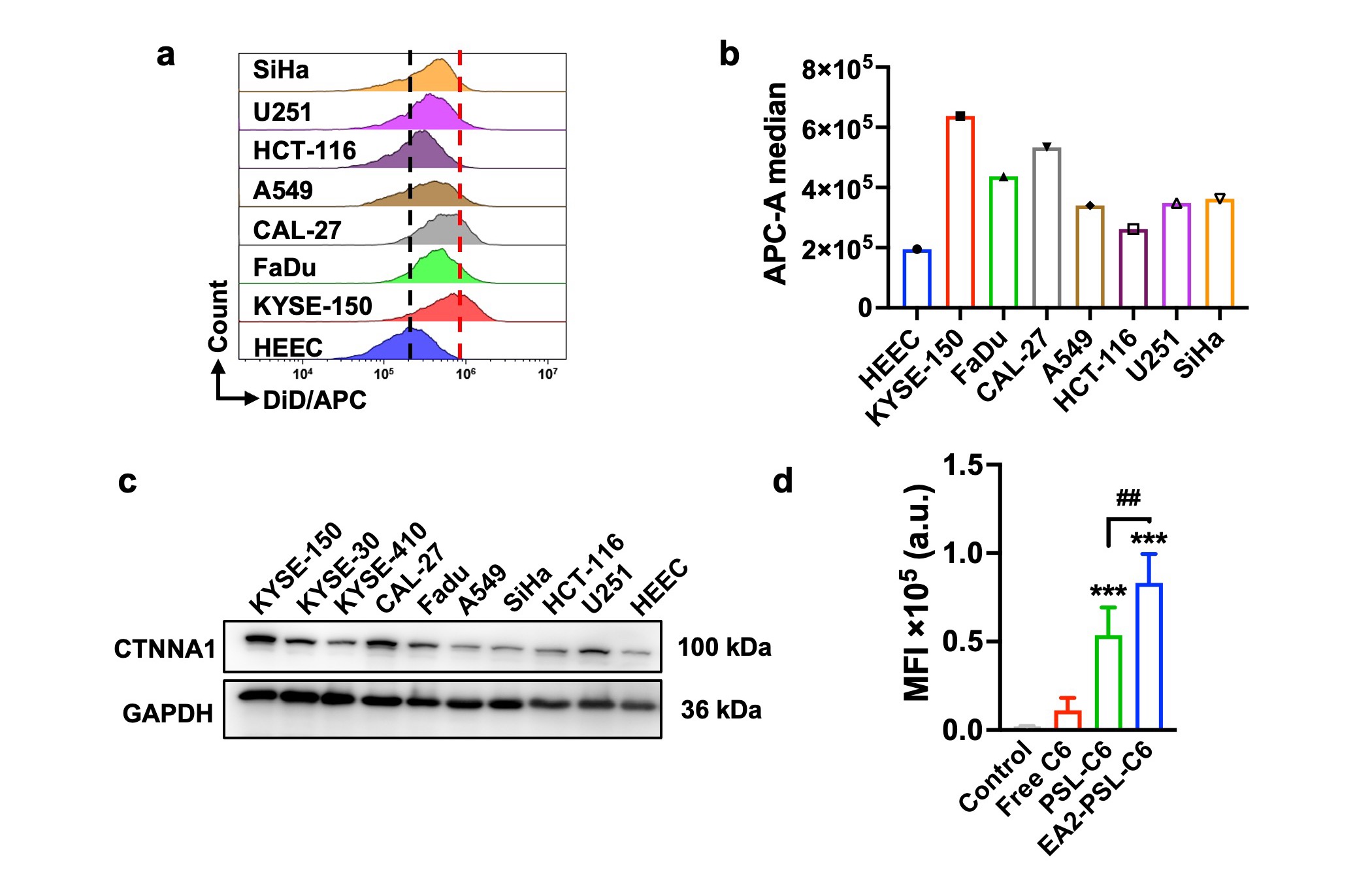
**

**Figure S11. Specific cellular uptake of EA2 aptamer-modified PSL.** **a-b**. Flow cytometry and mean fluorescence intensity analysis were conducted to assess specific cellular uptake. Various cancer cell lines, including KYSE-150, FaDu, CAL-27, A549, HCT-116, U251, SiHa, and HEEC**,** were incubated with EA2-PSL for 1 h, and then collected for flow cytometry. **c.** Relatively protein expression of CTNNA1 by Western blotting analysis. **d**. Statistical analysis of cellular uptake at 1 h using MFI. All data expressed as mean ± SD (n=3), statistical significance between different groups was obtained by one-way ANOVA using the Tukey’s post-test (**d**). ^***^ *p*<0.001, significant as compared to the free C6 group. ^##^ *p*<0.01, indicating statistical significance between the compared groups.


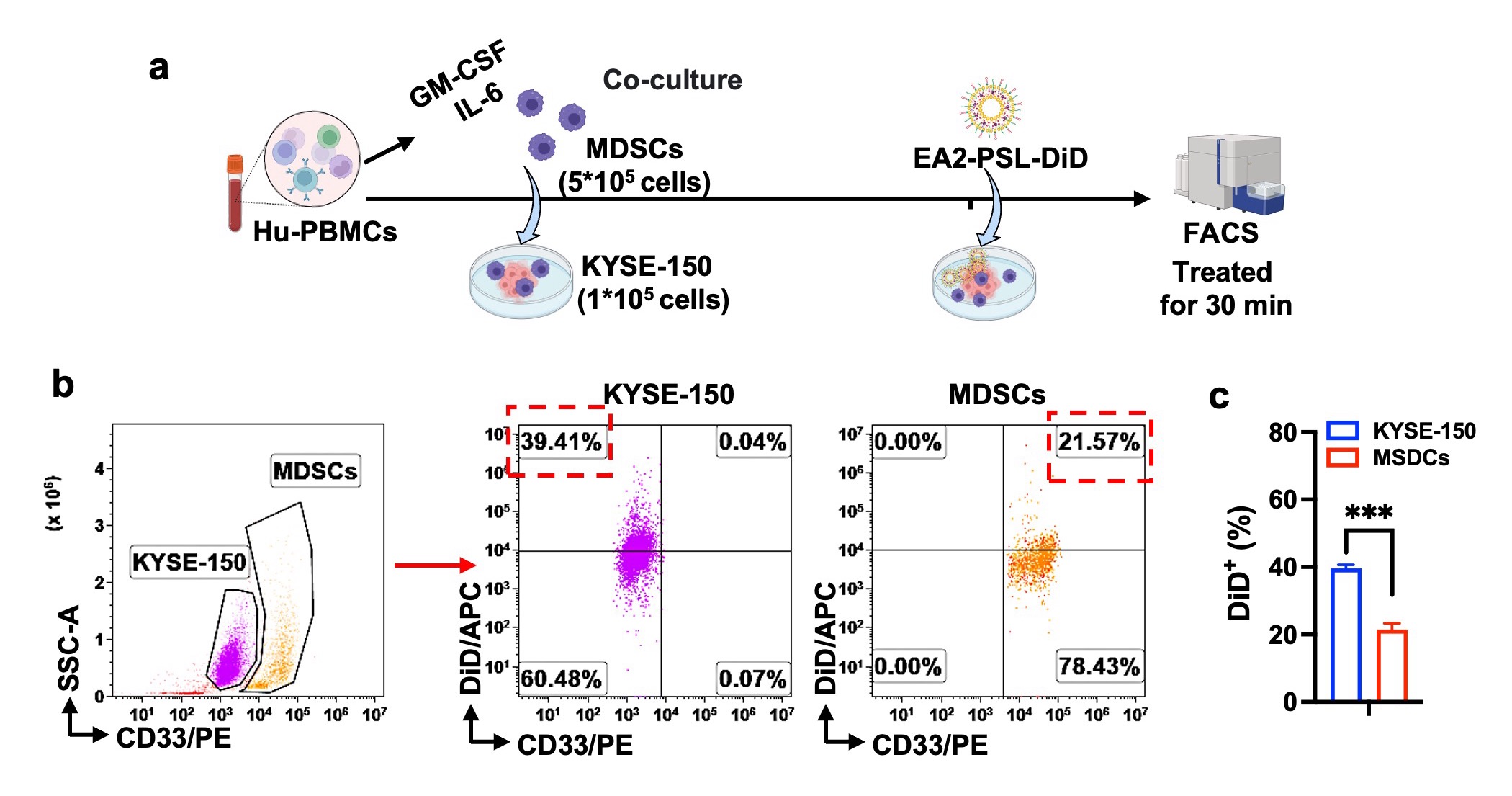


**Figure S12. Targeting of EA2-modified PSLs. a.** Schematic diagram of cellular uptake experiments in the coculture model of MDSCs with KYSE-150 cells. **b-c.** Flow cytometry analysis of the cellular uptake of KYSE-150 cells or MDSCs after incubation with PSL-DiD or EA2-modified PSL-DiD for 2 h. MDSCs were labelled with PE anti-human CD33 antibody, then cells were collected for flow cytometry. All data expressed as mean ± SD (n=3), statistical significance between different groups was obtained by an unpaired two-tailed Student’s t test (**c**), ^***^ *p*<0.001, indicating statistical significance between the compared groups.


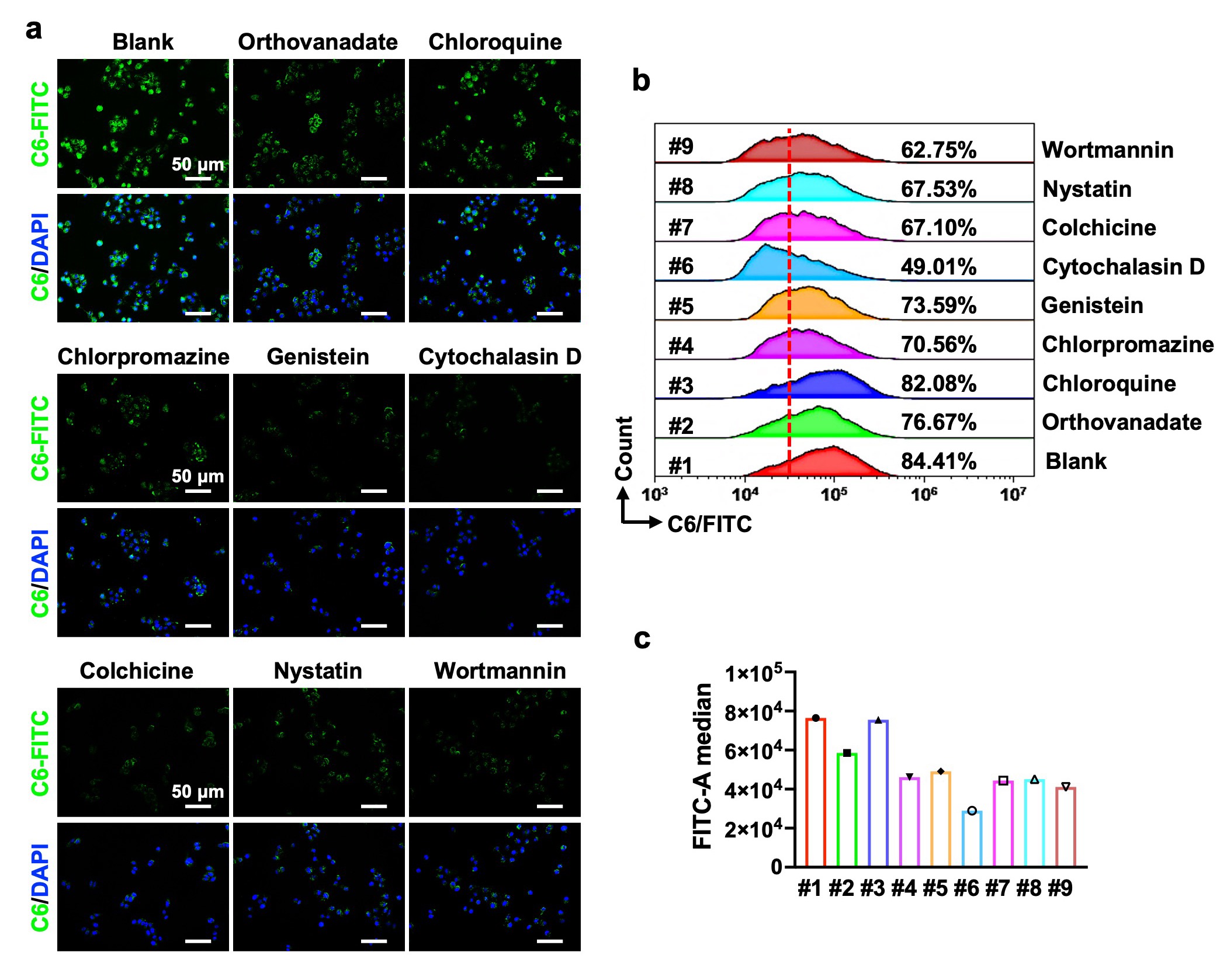


**Figure S13. Internalization mechanism of EA2-modified PSL. a.** Representative fluorescence images of internalization mechanism study. Scale bars, 50 μm. **b-c.** Flow cytometry and mean fluorescence intensity analysis were conducted to assess the inhibition effects of different endocytic inhibitors. KYSE-150 cells were incubated with various endocytic inhibitors, including genistein (caveolin-mediated endocytosis inhibitor, 200 μM), chlorpromazine (clathrin-mediated endocytosis inhibitor, 20 μM), colchicine (cytoskeleton-mediated endocytosis inhibitor, 50 μM), nystatin (caveolin-mediated endocytosis inhibitor, 5 μM), wortmannin (micropinocytosis inhibitor, 20 μM), sodium orthovanadate (dynein inhibitor, 10 μM), chloroquine (endosomal acidification inhibitor, 100 μM), and cytochalasin D (phagocytosis inhibitor, 20 μM) for 30 min, followed by incubation with EA2-PSL-C6 for 2h, and then cells were collected for confocal imaging or flow cytometry.

**
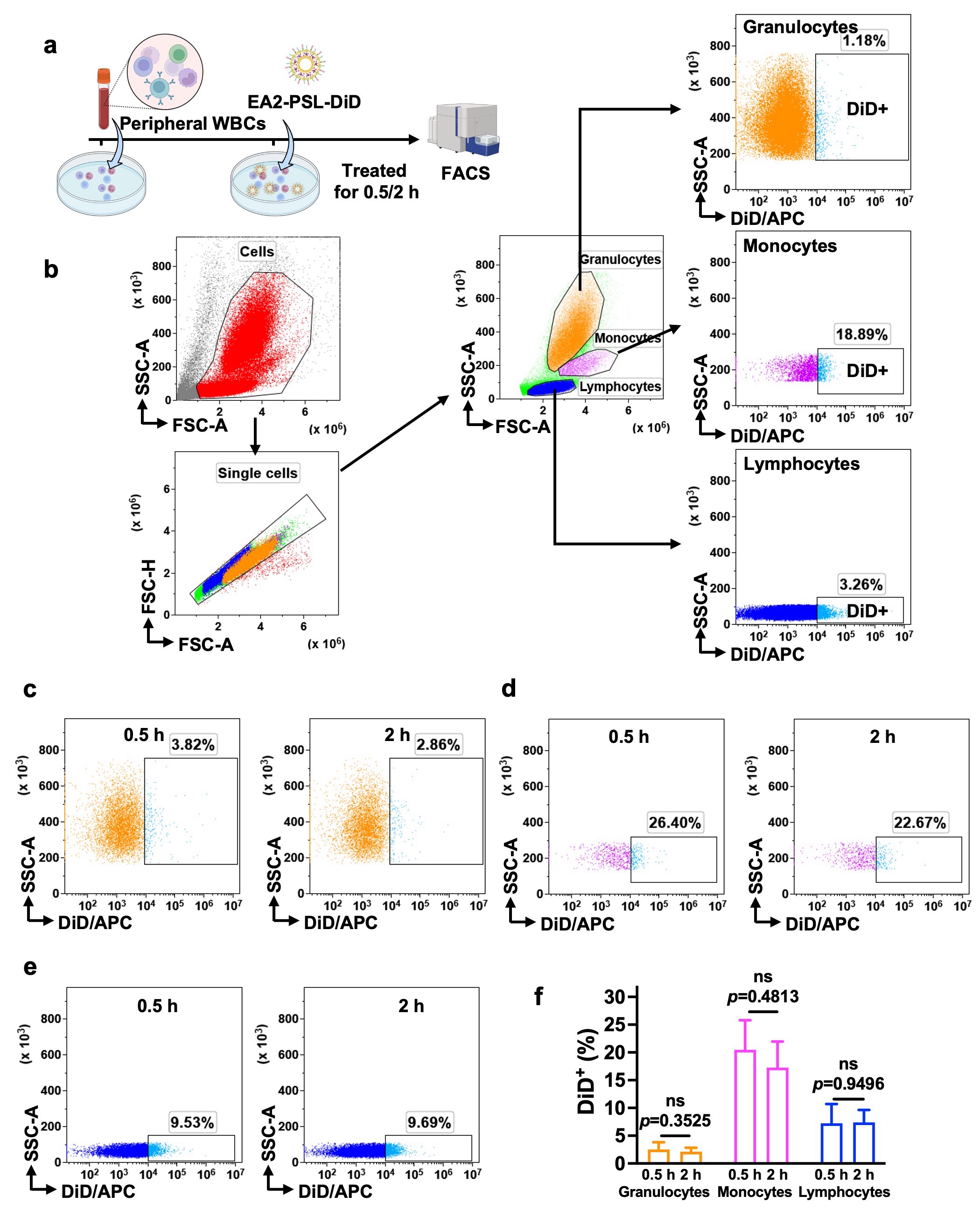
**

**Figure S14. Flow cytometry assay investigating the biodistribution of EA2-modified PSL in blood cells. a.** Schematic diagram of experiments. **b.** Flow gating strategy for analyzing blood cell flow cytometry data. **c-d.** The uptake of DiD-loaded PSLs in granulocytes (c), monocytes (d), and lymphocytes (e). **f.** Percentage of DiD-loaded PSLs bound to blood cells. Healthy human white blood cells were incubated with EA2-modified DiD-loaded PSLs for 0.5 and 2 h at 37 °C. The uptake of nanoparticles in WBCs was detected by flow cytometry. All data expressed as mean ± SD (n=3), statistical significance between different groups was obtained by one-way ANOVA using the Tukey’s post-test (**f**).

**
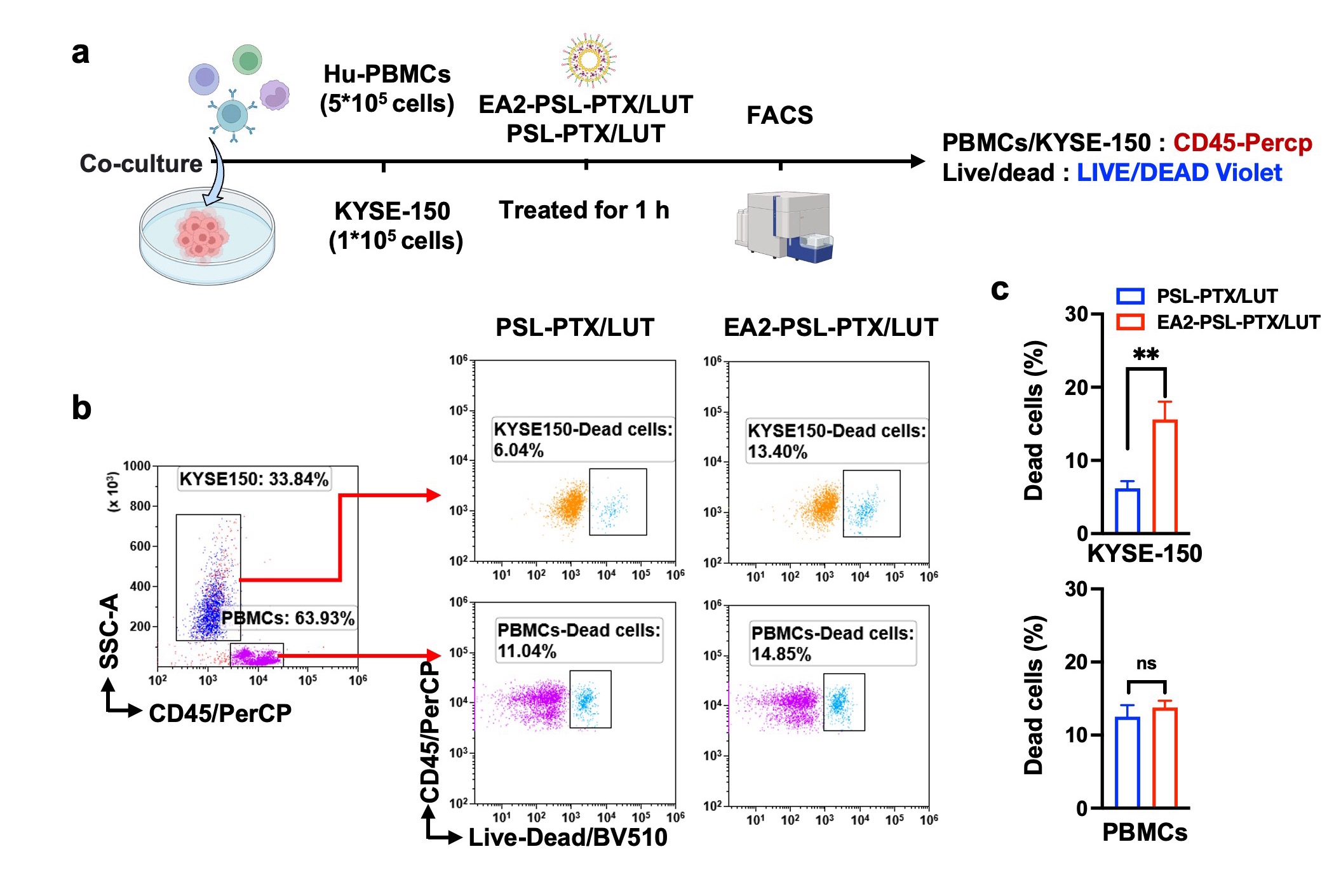
**

**Figure S15. Cytotoxicity of EA2-modified PSL.** **a.** Schematic diagram of cytotoxicity experiments in the coculture model of PBMCs with KYSE-150 cells. **b-c**. Flow cytometry analysis of the cytotoxicity in KYSE-150 cells or PBMCs after incubation with PSL-PTX/LUT or EA2-modified PSL- PTX/LUT for 1 h. PBMCs were labelled with PerCP anti-human CD45 antibody, and a live/dead stain was used to identify dead cells. All data expressed as mean ± SD (n=3), statistical significance between different groups was obtained by an unpaired two-tailed Student’s t test (**c**), ^**^ *p*<0.01, indicating statistical significance between the compared groups.

**
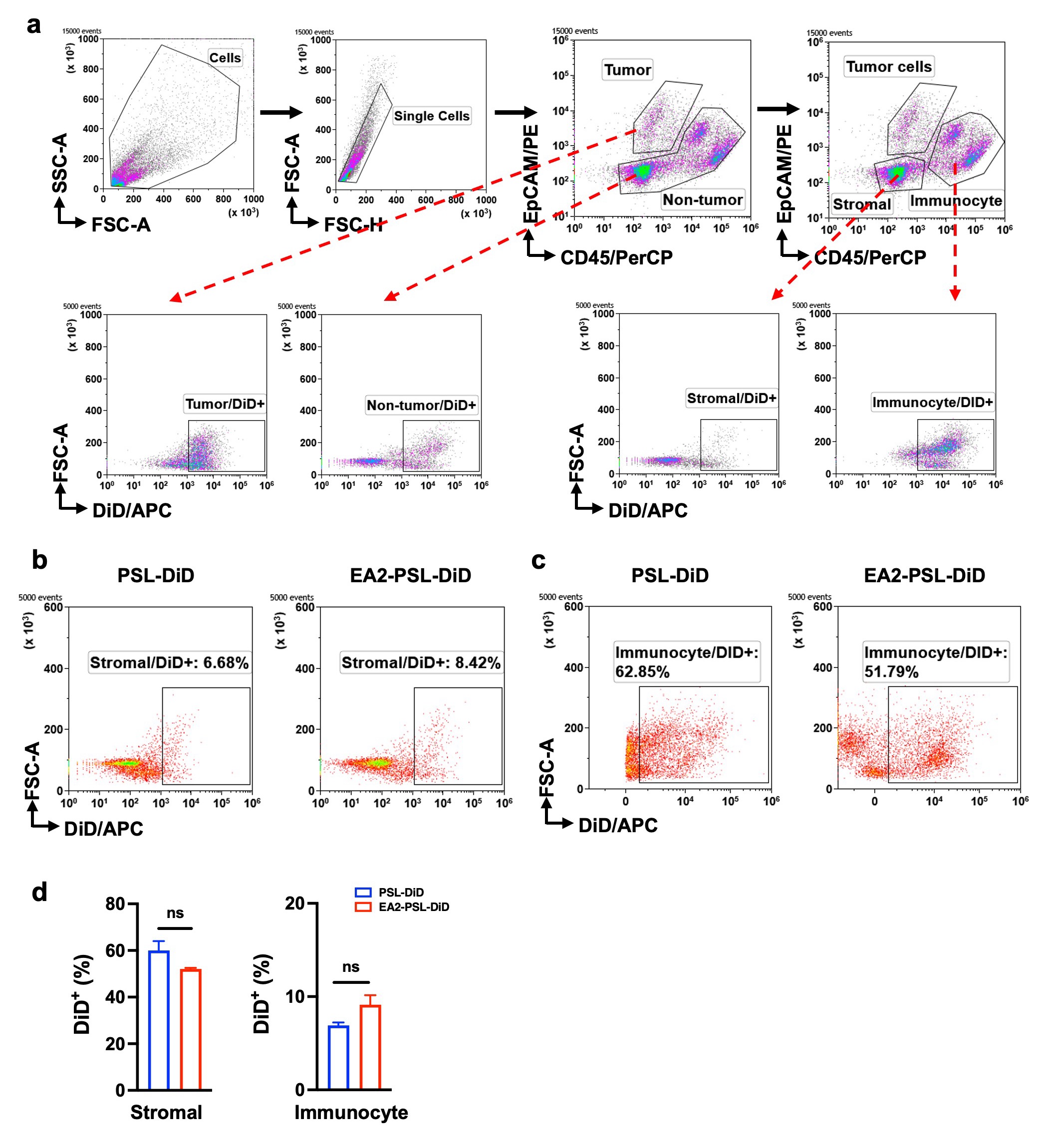
**

**Figure S16. Flow cytometry assay investigating the biodistribution of nanoparticles in tumors.** KYSE-150 tumor-bearing mice were intravenously injected with DiD-loaded PSL and EA2-PSL (at equivalent amount of DiD, 1 μg). After 24-h intravenous injection, tumors were harvested for flow cytometry assay. **a.** Flow gating strategy for analyzing the biodistribution of nanoparticles in different cells. **b-d**. The positive DiD^+^ portion of different nanoparticles in stromal cells (CD45^-^/EpCAM^-^), and immunocytes (CD45^+^/EpCAM^-^) were determined. All data expressed as mean ± SD (n=3), statistical significance between different groups was obtained by an unpaired two-tailed Student’s t test (**d**).

**
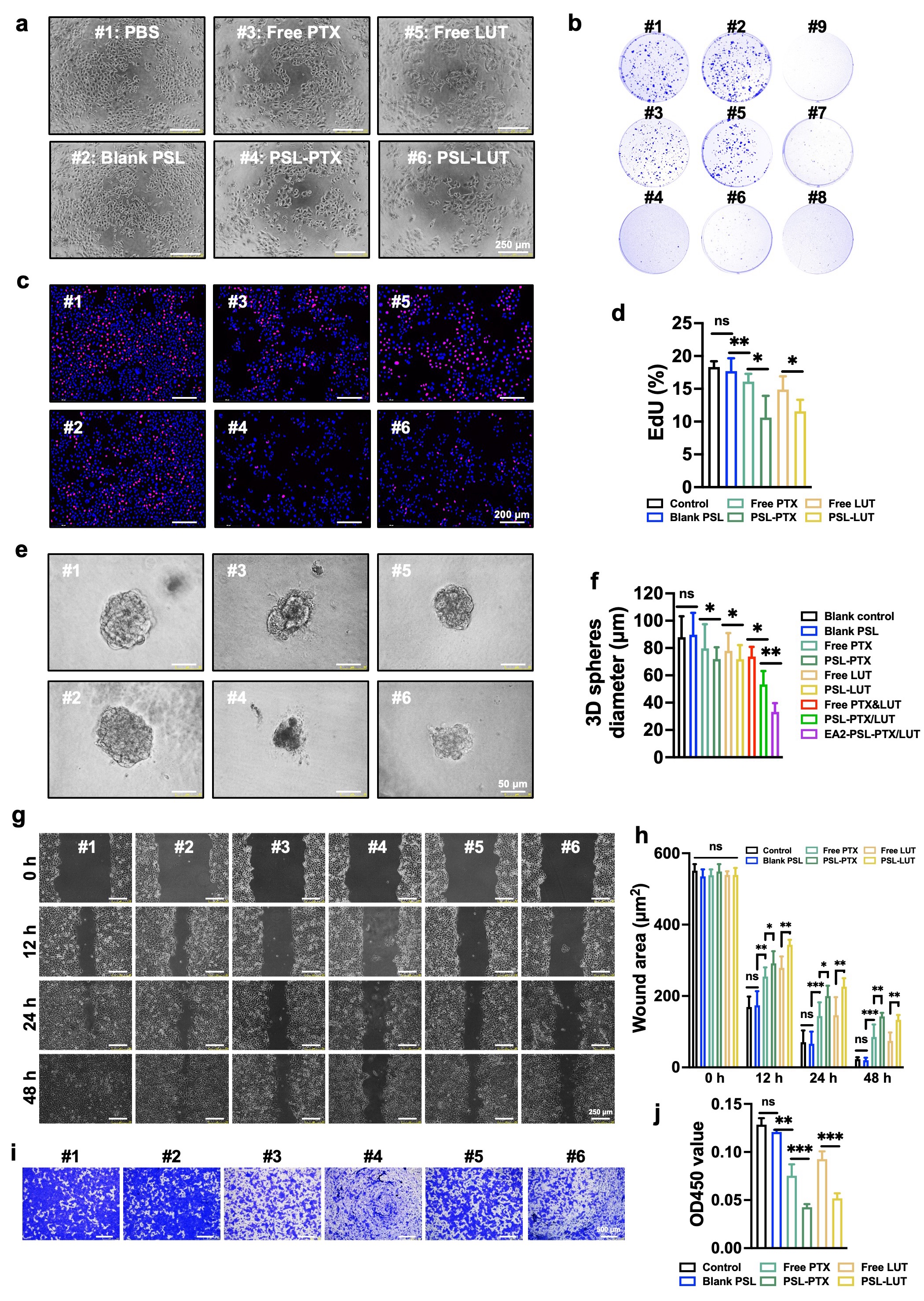
**

**Figure S17. *In vitro* cancer suppression eﬃciency of various treatments**. **a**. Representative morphological changes in PBS (#1), blank PSL (#2), PTX (#3), PTX-loaded PSL (#4), LUT (#5), LUT-loaded PSL (#6) groups at equimolar doses of PTX (7.5 nM) and LUT (15 μM). Scale bars, 250 μm. **b**. Colony formation assay was employed to access the clone-forming ability of KYSE-150 cells. **c-d**. Suppression effect of various treatments on proliferation of KYSE-150 cells by EdU assay. Scales bar, 200 μm. **e-f**. 3D tumor spheroid images showing the anticancer effects. Scale bars, 50 μm. **g-h**. Suppression effects of various treatments on migration abilities of KYSE-150 cells using wound-healing assay. Scale bars, 250 μm. **i-j**. Inhibition effects of various treatments on invasion abilities of KYSE-150 cells using a Transwell assay. Scale bars, 500 μm. All data expressed as mean ± SD (n=3), statistical significance between different groups was obtained by one-way ANOVA using the Tukey’s post-test (**d, f, h, j**). ^*^ *p*<0.05, ^**^ *p*<0.01, ^***^ *p*<0.001, indicating statistical significance between the compared groups.


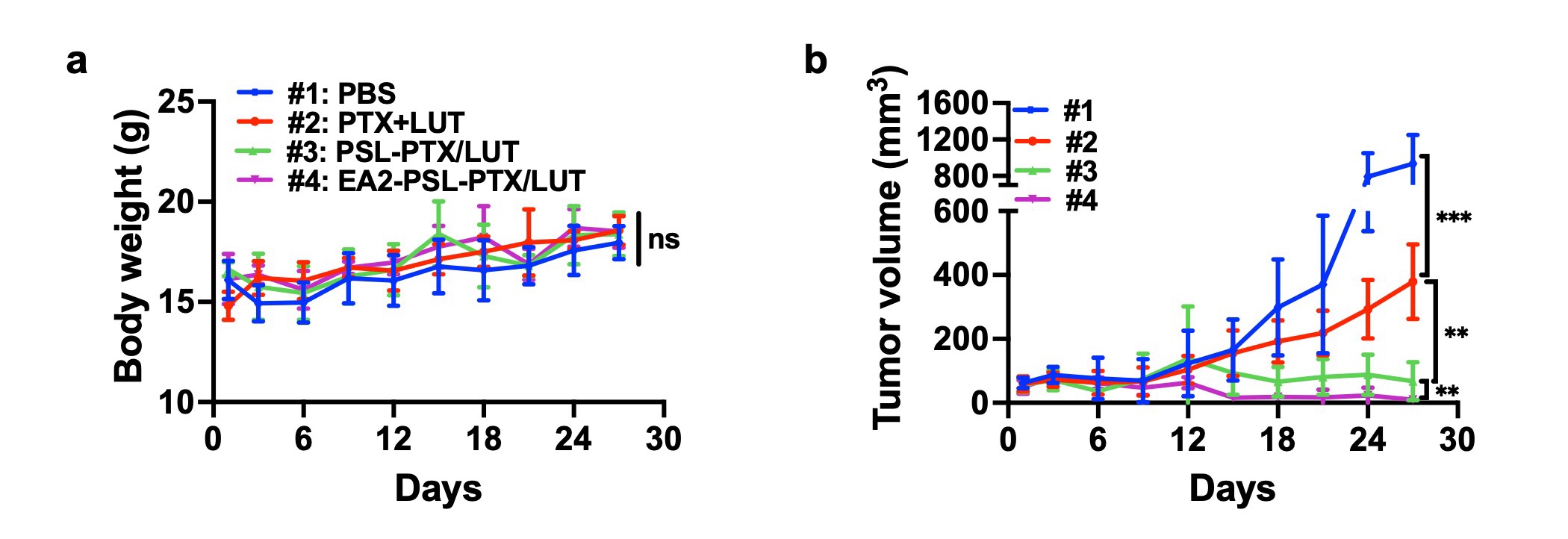


**Figure S18**. Body weight changes (**a**) and average tumor growth curves (**b**) of BALB/c nude mice bearing KYSE-150 tumors after injection with different formulations. All data expressed as mean ± SD (n=4), statistical significance between different groups was obtained by one-way ANOVA using the Tukey’s post-test (**a, b**), ^**^ *p*<0.01, ^***^ *p*<0.001, indicating statistical significance between the compared groups.


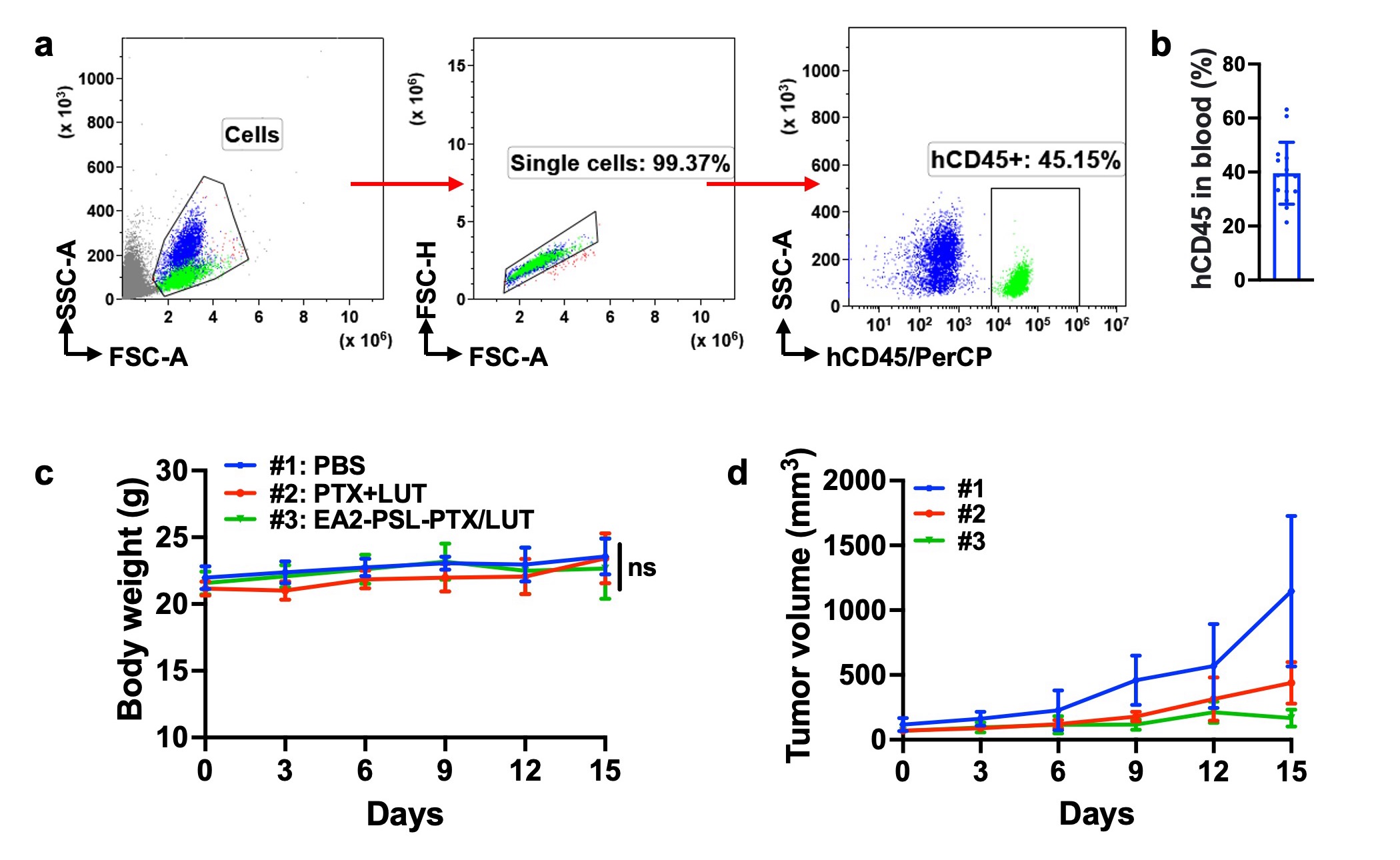


**Figure S19**. **a.** Flow gating strategy for analyzing percentages of human CD45^+^ cells in mice peripheral blood. **b.** Mean percentages of human CD45^+^ cells. **c-d.** Body weight changes (c) and average tumor growth curves (d) of PBMCs-engrafted mice bearing KYSE-150 tumors after injection with different formulations.


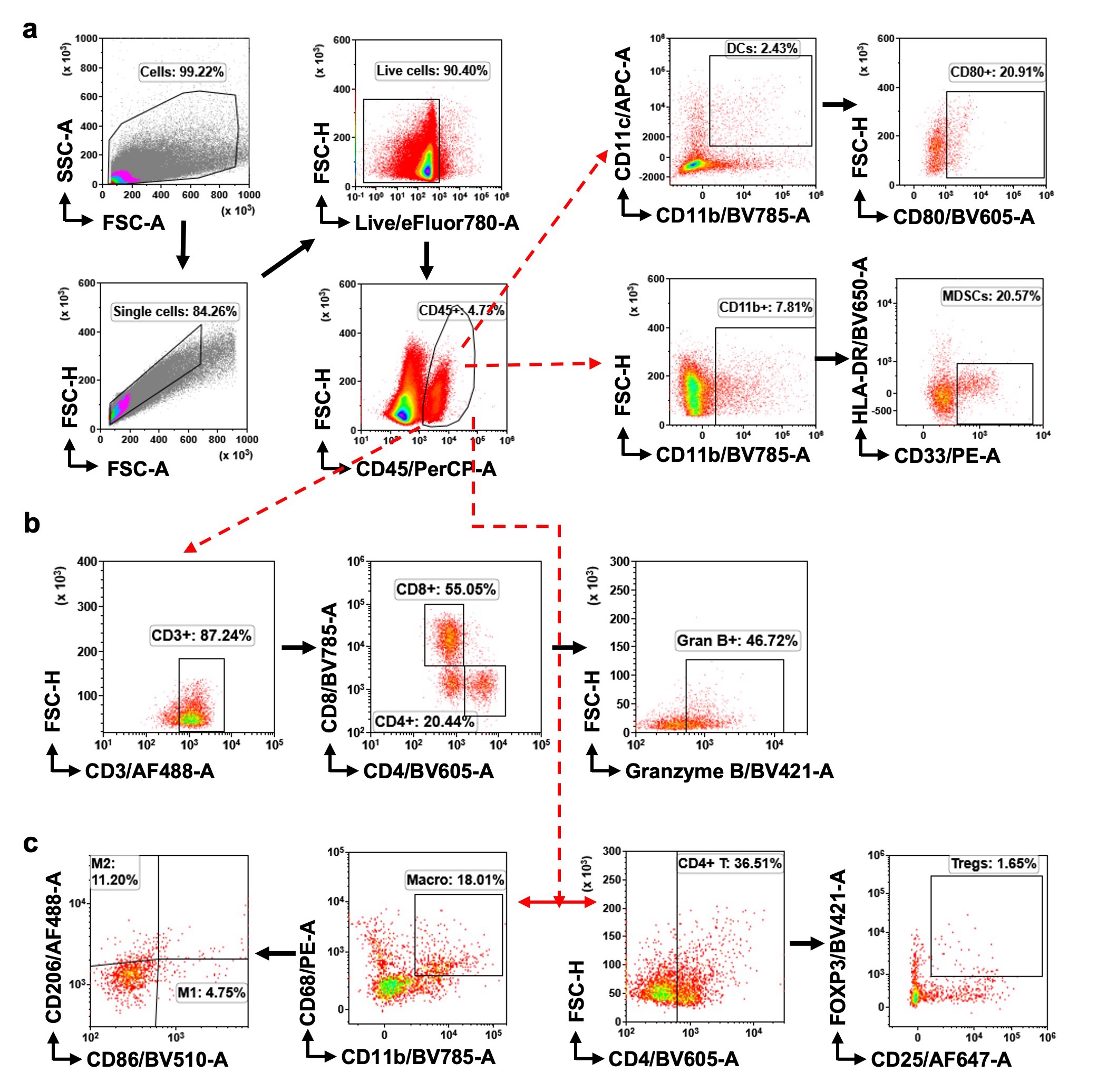


**Figure S20**. **Flow gating strategy for analyzing various immune cells in tumors. a.**

Immune cells (CD45^+^), MDSCs (CD11b^+^/CD33^+^/HLA-DR^-^), DCs (CD11b^+^/CD11c^+^), and matured DCs (CD80^+^) in tumors. **b.** CD8^+^ T cells (CD3^+^/CD8^+^), CD4^+^ T cells (CD3^+^/CD4^+^), and Granzyme B^+^ in CD8^+^ T cells. **c**. Tregs (CD4^+^/CD25^+^/FOXP3^+^), TAMs (CD11b^+^/CD68^+^), M1-type TAMs (CD86^+^/CD206^-^), and M2-type TAMs (CD206^+^/CD86^-^) in tumors.


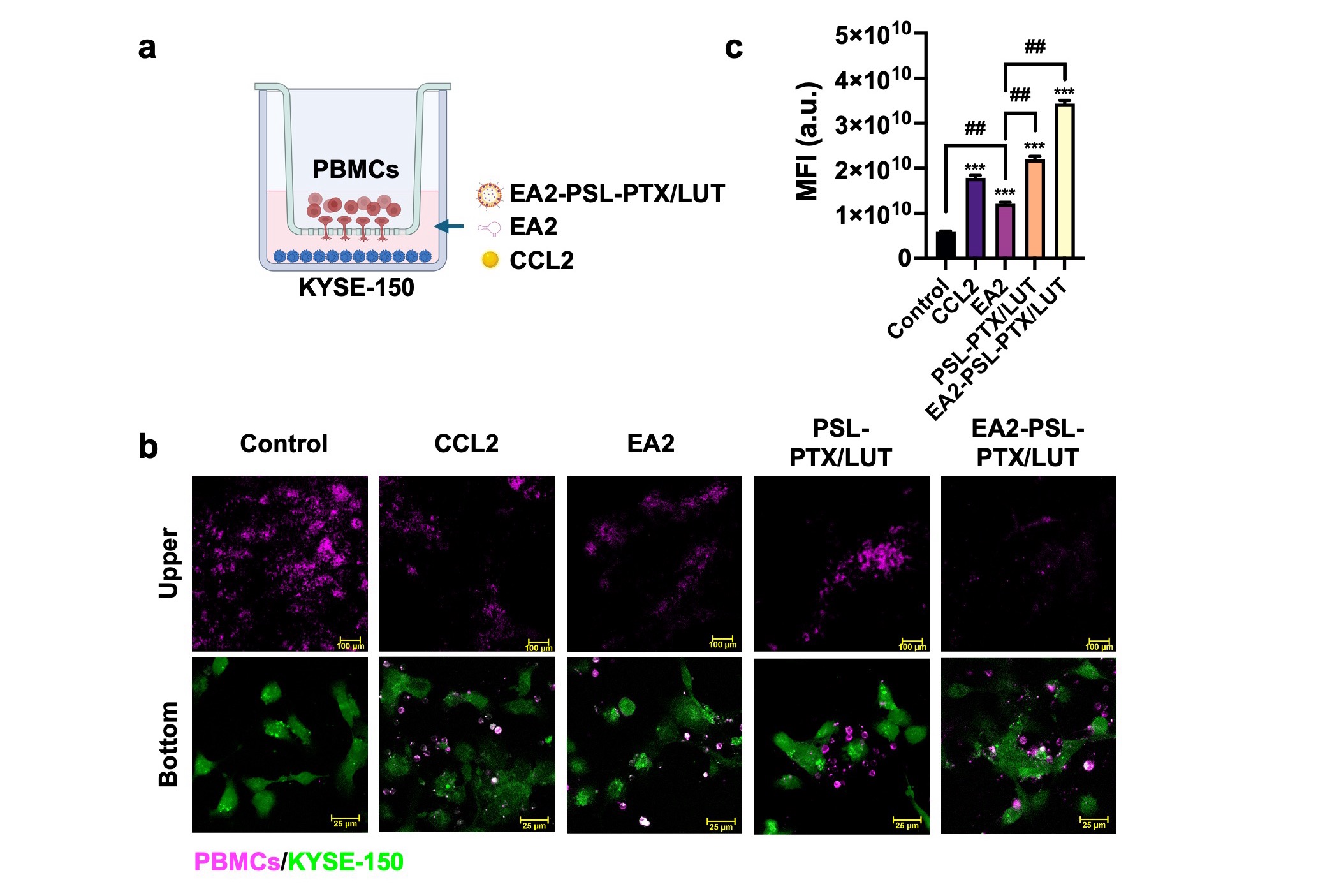


**Figure S21**. **Immune cell recruitment ability of EA2-PSL-PTX/LUT.** **a.** Illustration of an *in vitro* co-culture model. **b.** Chemotactic penetration of PBMCs by different agents (20 ng/mL CCL-2, 250 nM EA2, PSL-PTX/LUT, and EA2-PSL-PTX/LUT) in the upper and lower chamber. **c.** Fluorescence intensities of migrated PBMCs in lower chamber. All data expressed as mean ± SD (n=3), statistical significance between different groups was obtained by one-way ANOVA using the Tukey’s post-test (**c**). ^***^ *p*<0.001, indicating statistical significance as compared to the control group. ^##^ *p*<0.01, indicating statistical significance between the compared groups.

**3 Supporting Tables**

**Table S1**. Sequences of aptamer candidates.

| *Name* | *Random-region sequence (5’-3’)* |
| --- | --- |
| Random | 5’-AGCCTAAGCCTGTCCAGGAATCG-32N-ATGGCTTAGTGGCACGATTAGGTC-3’ |
| EA2 | 5’-AGCCTAAGCCTGTCCAGGAATCGTGCAGCCATAGCCTAAGCCTGTCCAGGAATCGATGGCTTAGTGGCACGATTAGGTC-3’ |
| Sgc8 | 5’-ATCTAACTGCTGCGCCGCCGGGAAAATACTGTACGGTTAGA-3’ |
| EGFR | 5’-Gcc uuA GuA AcG uGc uuu GAu Guc GAu ucG AcA GGA GGc-3’ |
| AS1411 | 5’-GGTGGTGGTGGTTGTGGTGGTGGTGG-3’ |

**Table S2**. siRNA senses for CTNNA1 RNA interference.

| *Name* | *Context sequence (5’-3’)* |
| --- | --- |
| si-negative control | sense: 5’-UUCUCCGAACGUGUCACGUTT-3’ |
|  | antisense: 5’-ACGUGACACGUUCGGAGAATT-3’ |
| si-CTNNA1-1 | sense: 5’-CCCUCUGUCCUCAGGUUAUUATT-3’ |
|  | antisense: 5’-UAAUAACCUGAGGACAGAGGGTT-3’ |
| si-CTNNA1-2 | sense: 5’-GCCCAUGGAUGAGAAUGAGUUTT-3’ |
|  | antisense: 5’-AACUCAUUCUCAUCCAUGGGCTT-3’ |
| si-CTNNA1-3 | sense: 5’-GCAGAUGUGCAUGAUUAUGAUTT-3’ |
|  | antisense: 5’-AUCAUAAUCAUGCACAUCUGCTT-3’ |

**Table S3**. Interaction of CTNNA1 and EA2

| **Type** | **CTNNA1** | **EA2** | **Energy** | **Distance** | **Type** | **CTNNA1** | **EA2** | **Energy** | **Distance** |
| --- | --- | --- | --- | --- | --- | --- | --- | --- | --- |
| DH | Lys78 | C46 | -6.42 | 3.74 | DH | Ser580 | G64 | -1.06 | 4.06 |
| D | Ile79 | C46 | -0.18 | 4.09 | DH | Asn581 | G64 | 7.14 | 3.9 |
| D | Glu82 | C46 | -0.03 | 4.41 | D | Pro585 | G64 | 1.18 | 3.82 |
| DH | Ile189 | G44 | 10.22 | 3.83 | DH | Pro585 | T65 | -1.23 | 3.73 |
| D | Ile189 | T45 | -0.28 | 4.28 | D | Thr588 | G66 | -0.1 | 4.31 |
| D | Met190 | T45 | -0.09 | 4.28 | **DIH** | **Arg872** | **A52** | **-9.12** | **3.44** |
| D | Ala192 | G44 | -0.04 | 4.19 | **DI** | **Arg872** | **T53** | **-14.16** | **3.69** |
| DH | Lys193 | G44 | 0.34 | 3.96 | **DI** | **Arg872** | **G55** | **-13** | **3.61** |
| DH | Lys193 | T45 | -2.5 | 3.95 | DI | Lys874 | A33 | -0.91 | 4.16 |
| D | Arg496 | C29 | 11.66 | 3.85 | D | Lys874 | T57 | -0.1 | 4.26 |
| D | Asp500 | T32 | -0.04 | 4.23 | **DH** | **Gln875** | **G40** | **-1.2** | **3.73** |
| D | Asp504 | T32 | 0.02 | 4.14 | **D** | **Gln875** | **C41** | **22.3** | **3.82** |
| D | Arg546 | G66 | -0.03 | 4.21 | **D** | **Gln875** | **C42** | **0** | **4.44** |
| DH | His554 | C30 | -0.59 | 3.71 | **D** | **Gln875** | **A51** | **-0.03** | **4.03** |
| D | Thr557 | C30 | -0.01 | 4.2 | **DH** | **Gln875** | **A56** | **-0.59** | **4.06** |
| DH | Thr557 | A31 | -1.68 | 4.07 | **D** | **Asp876** | **C41** | **0.03** | **4.02** |
| **D** | **Asp561** | **A31** | **-0.03** | **4.23** | **DH** | **Asp876** | **C42** | **-1.06** | **4.08** |
| **D** | **Asp561** | **T32** | **0.14** | **4.18** | **DH** | **Asp876** | **T57** | **-1.29** | **3.92** |
| **D** | **Asp561** | **A33** | **0.86** | **3.71** | **D** | **Asp876** | **G58** | **-0.15** | **4.25** |
| D | Pro565 | G34 | -0.08 | 3.78 | DH | Lys881 | T32 | 0.29 | 4.15 |
| D | Lys577 | A63 | 0.5 | 4.12 | D | Lys883 | T32 | 0.15 | 4.36 |

Type represents the type of interaction, where I stands for ionic interaction, H for hydrogen bond, D for van der Waals force, A for pi-pi or pi-H stacking effect. Energy represents the interaction energy, with unit kcal/mol.

**Table S4**. The entrapment efficiency (EE) of PSL-PTX/LUT were evaluated at varying molar ratios of DOPE to CHEMS.

| *Formulation* | *DOPE to CHEMS molar ratio* | *DOPE (mmoL)* | *CHEMS (mmoL)* | *TPGS (mmoL)* | *LUT/PTX (mmoL)* | *EE of LUT %* | *EE of PTX %* |
| --- | --- | --- | --- | --- | --- | --- | --- |
| 1 | 55:45 | 5.5 | 4.5 | 0.25 | 0.427/0.043 | 88.08 ± 4.69 | 93.74 ± 1.57 |
| 2 | 60:40 | 6.0 | 4.0 |  |  | 91.78 ± 3.18 | 95.36 ± 1.17 |
| 3 | 65:35 | 6.5 | 3.5 |  |  | 94.51 ± 3.71 | 95.52 ± 1.14 |

**Table S5**. EE characteristics of PSL-PTX/LUT were evaluated at different drug-to-lipid molar ratio.

| *Formulation* | *Drug-to-lipid molar ratio* | *LUT/PTX (mmoL)* | *DOPE (mmoL)* | *CHEMS (mmoL)* | *TPGS (mmoL)* | *EE of LUT %* | *EE of PTX %* |
| --- | --- | --- | --- | --- | --- | --- | --- |
| 1 | 32:1 | 0.320/0.032 | 6 | 4 | 0.25 | 91.29 ± 4.56 | 91.35 ± 1.12 |
| 2 | 24:1 | 0.427/0.043 |  |  |  | 91.78 ± 3.18 | 95.36 ± 1.17 |
| 3 | 16:1 | 0.640/0.064 |  |  |  | 86.36 ± 6.31 | 96.00 ± 2.28 |
| 4 | 12:1 | 0.854/0.085 |  |  |  | 52.27 ± 6.73 | 68.63 ± 0.97 |

**Table S6**. EE characteristics of PSL-PTX/LUT were evaluated at different levels of TPGS addition.

| *Formulation* | *TPGS (mmoL)* | *DOPE (mmoL)* | *CHEMS (mmoL)* | *LUT/PTX (mmoL)* | *EE of LUT %* | *EE of PTX %* |
| --- | --- | --- | --- | --- | --- | --- |
| 1 | 0.15 | 6 | 4 | 0.427/0.043 | 46.82 ± 5.12 | 61.21 ± 6.66 |
| 2 | 0.20 |  |  |  | 80.17± 1.57 | 78.71 ± 2.89 |
| 3 | 0.25 |  |  |  | 91.78 ± 3.18 | 95.36 ± 1.17 |
| 4 | 0.30 |  |  |  | 90.63± 2.23 | 94.47 ± 1.53 |

**4 References**

[1] T.-C. Chou, T. A. Shapiro, J. Fu, J. H. Chou, G. S. Ulrich-Merzenich, *Synergy* **2019**, *9*, 100049.

[2] J. R. Guarin, J. P. Fatherree, M. J. Oudin, *Matrix Biology* **2022**, *112*, 20.

[3] F. Xie, J. Qiu, C. Sun, L. Feng, Y. Jun, C. Luo, X. Guo, B. Zhang, Y. Zhou, Y. Wang, L. Zhang, Q. Wang, *Adv Sci* **2024**, 2309084.
